# Supplementary material for: Ligand-Based Design of Selective Peptidomimetic uPA and TMPRSS2 Inhibitors with Arg Bioisosteres
Source: Int J Mol Sci. 2024 Jan 23;25(3):1375. doi: 10.3390/ijms25031375 (PMC10855164; doi:10.3390/ijms25031375)
Supplement: Supplementary file 1 [file ijms-25-01375-s001.zip › ijms-2832275-supplementary.pdf]

Supporting information

Ligand-Based Design of Selective Peptidomimetic uPA and TMPRSS2 Inhibitors with Arg Bioisosteres

Patrick Müller, Collin Zimmer, Ariane Frey, Gideon Holzmann, Annabelle Carolin Weldert and Tanja Schirmeister*

Institute of Pharmaceutical and Biomedical Sciences, Johannes Gutenberg University Mainz, Staudinger Weg 5, D-55128 Mainz, Germany; muelpat@uni-mainz.de (P.M.); cozimmer@uni-mainz.de (C.Z.); arfrey@uni-mainz.de (A.F.); gholzman@students.uni-mainz.de (G.H.); anwelder@uni-mainz.de (A.C.W.)

* Correspondence: schirmei@uni-mainz.de; Tel.: +49-6131-39-25742

Content

**Protein Similarity Calculations**	2
**Fluorometric inhibition assays**	3–4
**Protein expression and purification**	4
**Absorption spectra**	5–6
**Calculation of physicochemical properties**	7
**Stability studies**	8–10
**Parallel Artificial Membrane Permeation Assay**	10–11
**Synthesis**	11–35
**NMR-spectra and chromatograms**	36–59
**References**	60

Protein Similarity Calculations

The similarity of the investigated serine proteases was compared using MOE 2022.02. Their biological assemblies were loaded using their pdb accession codes (uPA: 7VM4, TMPRSS2: 7MEQ, tPA: 1BDA, Factor Xa: 1XKB, Thrombin: 1D6W, Matriptase 1: 2GV6), aligned, and superimposed. For similarity calculations, only the binding sites were selected.

binding site alignment similarity

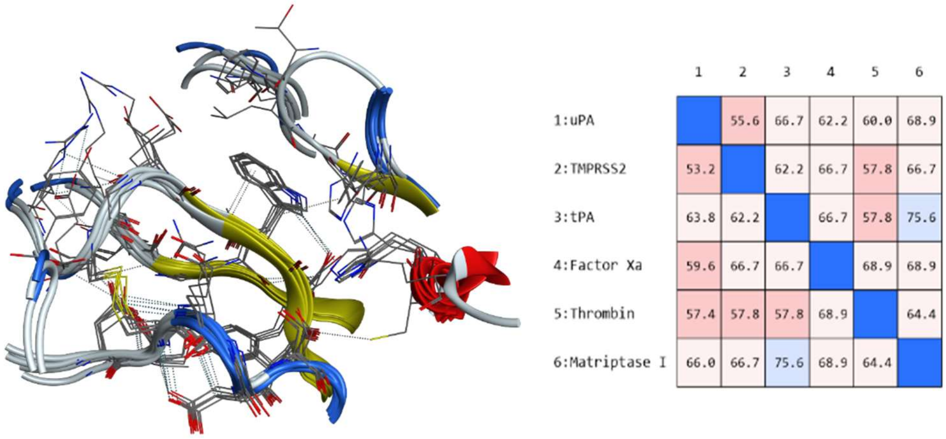

	1	2	3	4	5	6
1:uPA		55.6	66.7	62.2	60.0	68.9
2:TMPRSS2	53.2		62.2	66.7	57.8	66.7
3:tPA	63.8	62.2		66.7	57.8	75.6
4:Factor Xa	59.6	66.7	66.7		68.9	68.9
5:Thrombin	57.4	57.8	57.8	68.9		64.4
6:Matriptase I	66.0	66.7	75.6	68.9	64.4	

Figure S1: (left) visual binding site alignment of all six serine proteases, (right) matrix for calculated sequence similarities of the binding sites.

Fluorometric inhibition assays

The inhibitory activities of the compounds against the proteases were determined by enzyme inhibition assays with fluorogenic or colorimetry-based substrates. The fluorescence, caused by cleaved AMC from the fluorogenic substrates (uPA, matriptase, TMPRSS2, thrombin), was measured in white flat-bottom 96-well plates from Greiner Bio-One using a Tecan Infinite F200 Pro plate reader. The absorption, caused by cleaved *p*NA from colorimetric substrates (factor Xa and tPA), was measured in transparent flat-bottom 96-well plates from Greiner Bio-One using a Tecan Spark 10M plate reader. All measurements with the main-target proteases (uPA and TMRPSS2) were performed as triplicates and with the off-target proteases (matriptase, factor Xa, tPA, thrombin) as duplicates. The substrates and the compounds were prepared as stock solutions in DMSO. Each well contained a total volume of 200 µL, consisting of 185 µL buffer, 5 µL inhibitor in DMSO or pure DMSO as negative control, 5 µL substrate in DMSO and 5 µL enzyme solution in buffer. Dilution series were prepared for the determination of the inhibition constants. The fluorescence signal and absorbance were measured every 30 s for 10 min at 25 or 37 °C with the corresponding excitation/emission (λ_{ex} = 380 nm / λ_{em} = 460 nm) and absorbance (λ_{abs} = 405 nm) wavelengths, depending on the targeted protease. IC_{50} values for the reversible inhibitors were calculated with Graphpad Prism 9 by fitting the remaining enzymatic activity to the four parameter IC_{50} equation with Y [%] as the residual enzyme activity , Y_{max} as the maximum value of the dose response curve at inhibitor concentrations [I] = 0 µM, Y_{min} as the minimum value at high inhibitor concentrations and *s* as the hill coefficient.[1] The fluorescence progress curves and sigmoidal dose-response curve for inhibition of uPa by compound **38** is shown exemplary in Figure **S2**.

$$y = \frac{y_{\max} - y_{\min}}{1 + \left(\frac{[\mathrm{I}]}{\mathrm{IC}_{50}}\right)^{s}} + y_{\min} \qquad 1$$

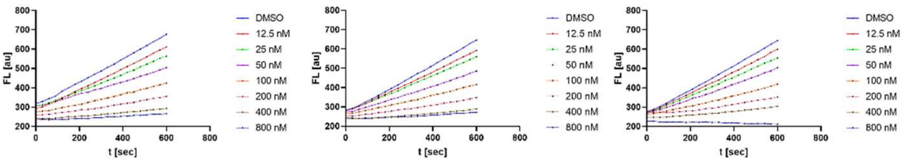

38

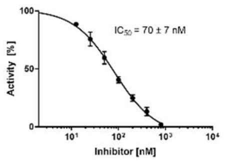

Figure S2: (left) Fluorescence progress curves for inhibition of uPa by cpd. **38**. (right) Plot showing the respective IC_{50} value from sigmoidal fit.

Due to the dependence of the IC_{50} value on the substrate affinity and concentration, the K_i values were calculated with the Cheng-Prusoff equation, using the final substrate concentration [*S*] and the Michaelis-Menten constant K_M, (2) for appropriate comparison of the inhibitory activities to the other enzymes and inhibitors.[1]

$$K_{\mathrm{i}} = \frac{\mathrm{IC}_{50}}{1 + \frac{[S]}{K_{\mathrm{M}}}} \qquad 2$$

K_M values were determined by fitting to the Michaelis-Menten equation using Graphpad Prism 9 with *v* [Δ*F*/min] as the substrate hydrolysis rate, v_{max} as the maximum slope of the dose-response curve, and the substrate [*S*] concentration.[1] The different substrates were serially diluted in DMSO. The measurement was done in analogy to the determination of the inhibitory activity, but with 5 µL pure DMSO instead of inhibitor solution. The fluorescence progress curves and Michaelis-Menten curve of the specific substrate of uPA is shown exemplarily in Figure **S3**.

$$v = \frac{v_{\max} \cdot [\mathrm{S}]}{K_{\mathrm{M}} + [\mathrm{S}]} \qquad 3$$

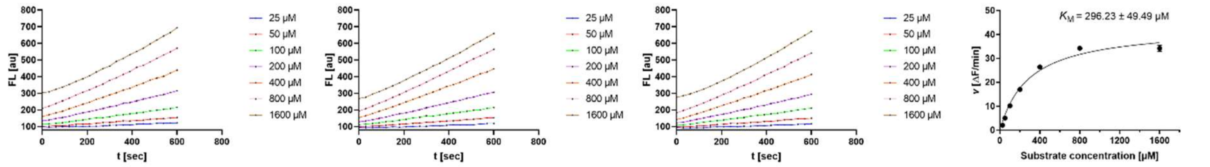

Figure S3: (left) Fluorescence progress curves of cleavage of Z-Gly-Gly-Arg-AMC by uPA. (right) Michaelis-Menten curve showing the respective K_M value.

Buffers and substrates

The following buffers and substrates were used for the respective assays: **uPA** (50 mM Tris HCl pH = 7.9, 150 mM NaCl, 10 mM $CaCl_2$, 0.005% TX-100, 240 µM Z-Gly-Gly-Arg-AMC (K_M: 296.23 ± 49.49 µM), 37 °C, 9 U/mL uPA)[2]; **TMPRSS2** (25 mM Tris HCl pH = 8.0, 150 mM NaCl, 5 mM $CaCl_2$, 0.001% TX-100, 100 µM Boc-Gln-Ala-Arg-AMC (K_M: 68.63 ± 6.64 µM), 25 °C, 3 nM TMPRSS2)[3]; **matriptase** (50 mM Tris HCl pH = 8.0, 150 mM NaCl, 5 mM $CaCl_2$, 0.0001% TX-100, 100 µM Boc-Leu-Arg-Arg-AMC (K_M: 36.13 ± 5.78 µM, 25 °C, 6 nM matriptase)[4]; **tPA** (50 mM Tris HCl pH = 8.3, 250 µM *N*-methylsulfonyl-*D*-Phe-Gly-Arg-*p*NA (K_M: 148.89 ± 29.72 µM), 37 °C, 0.4 ng/µL tPA)[5]; **thrombin** (50 mM Tris HCl pH = 8.0, 100 mM NaCl, 5 mM $CaCl_2$, 0.01% Tween-20, 200 µM Z-Gly-Gly-Arg-AMC (K_M: 49.70 ± 7.15 µM), 25 °C, 10 nM thrombin)[6]; **factor Xa** (50 mM Tris HCl pH = 7.5, 150 mM NaCl, 10 mM $CaCl_2$, 0.005% Brij-35, 100 µM Z-*D*-Arg-Gly-Arg-*p*NA (K_M: 249.12 ± 21.59 µM), 37 °C, 0.1 ng/µL factor Xa)[7].

Human uPa and tPA was purchased from Sigma Aldrich, TMPRSS2 from Cusabio, factor Xa and thrombin from Bio-techne. Matriptase was expressed under the conditions described below.

Protein expression and purification of matriptase 1

Recombinant human matriptase 1 was expressed as described previously with slight adaptations.[8,9] In short, the pQE-30 vector containing the zymogen of the catalytic domain of matriptase (uniport: Q9Y5Y6 aa 596–855) with an *N*-terminal hexa-histidine tag, was transformed into competent *Escherichia coli* (*E. coli*) BL21 Gold (DE3) cells (Agilent Technologies, Santa Clara, CA, USA). The transformed bacteria were grown at 37 °C and 160 rpm in LB medium (10 L) containing 100 µg/mL ampicillin. After reaching an optical density (OD_{600}) of ~0.8 overexpression was induced by adding 1 mM isopropyl-β-D-thiogalactopyranosid (IPTG) and the cells were incubated over night at 20 °C. Cells were harvested by centrifugation (9000 rpm at 4 °C for 15 min), resuspended in 250 mL cold lysis buffer (50 mM TRIS·HCl pH 8.0, 10% (v/v) glycerol, 300 mM NaCl, 0.1% (v/v) $Triton_{X-100}$, RNase, DNase, lysozyme, 1 mM dithiothreitol (DTT)) and lysed by sonication (Sonoplus HD 2200; Bandelin, Berlin, Germany). Inclusion bodies were isolated by centrifugation (20000 rpm at 4 °C for 1 h) and resuspended in 500 mL solubilization buffer (50 mM TRIS·HCl pH 8.0, 5% (v/v) glycerol, 6 M urea, 20 mM imidazole). After stirring the suspension overnight at 4 °C the mixture was centrifuged (20000 rpm at 4 °C for 1 h) again to remove insoluble impurities. Matriptase 1 was purified by immobilized metal affinity chromatography (IMAC) using a HisTrap HP 5 ml column (Cytiva Europe GmbH, Freiburg im Breisgau, Germany). The protein was washed with 5 column volumes (CV) wash buffer (50 mM TRIS·HCl pH 8.0, 6 M urea, 20 mM imidazole), and elution was achieved with a linear gradient of elution buffer (50 mM TRIS·HCl pH 8.0, 6 M urea, 200 mM imidazole). Matriptase 1 containing fractions were pooled and refolded by two-step dialysis against 2 L of dialysis buffer 1 (50 mM TRIS·HCl pH 9.0, 1 mM *β*-ME, 3 M urea) and 2 L of dialysis buffer 2 (50 mM TRIS·HCl pH 9.0, 1 mM β-ME) at 4 °C for > 8 h each. For further purification, anion exchange chromatography (AEX) on a HiTrap 5 mL column (Cytiva Europe GmbH) was utilized. Dialysis buffer 2 was used as AEX wash buffer (5 CV) and Matriptase was eluted with a linear gradient of dialysis buffer 2 and AEX elution buffer (50 mM TRIS·HCl pH 9.0, 1 mM β-ME, 1 M NaCl). Fractions containing matriptase, were pooled, flash-frozen in liquid nitrogen and stored at −80°C.

Absorption spectra.

Method: Measurement setup for UV spectroscopy consisted of UV-transparent measurement plate (Greiner UV-Star®, 655801), Tecan Spark 10M® well plate reader, 200 µL sample volume (5% DMSO/Tris buffer pH 7.4), λ = 250–400 nm.

14a

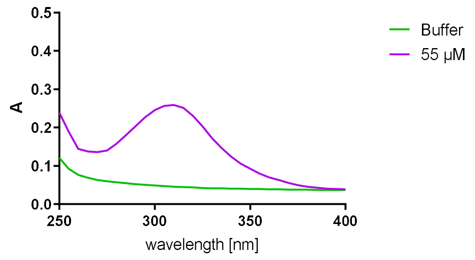

14a

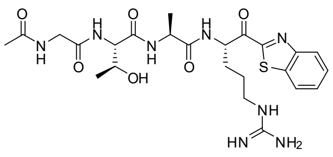

λ_{max} = 310 nm

33

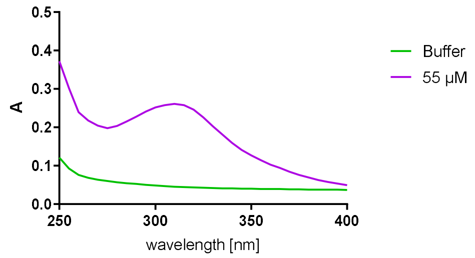

33

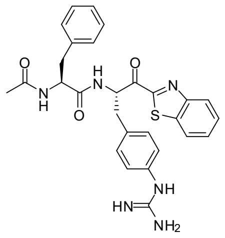

λ_{max} = 310 nm

14f

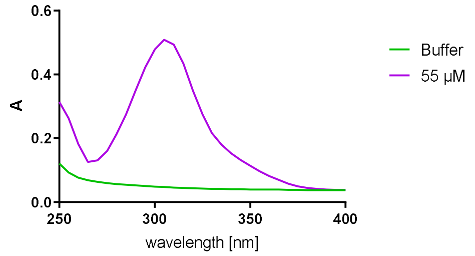

14f

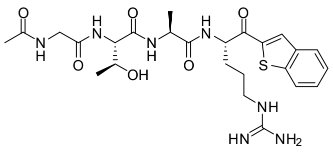

λ_{max} = 305 nm

14b

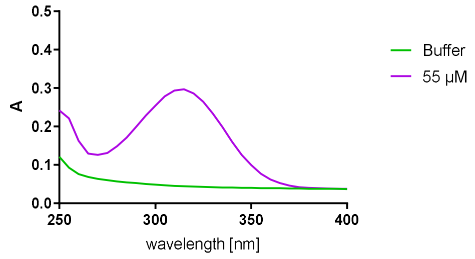

14b

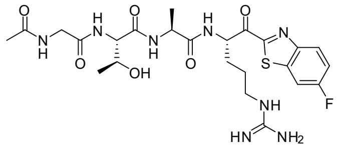

λ_{max} = 315 nm

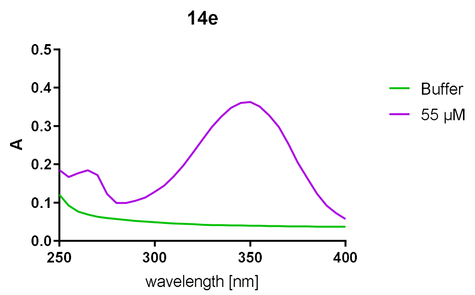

14e

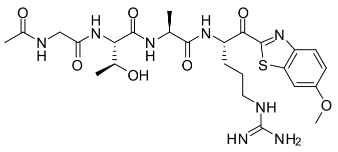

λ_{max} = 350 nm

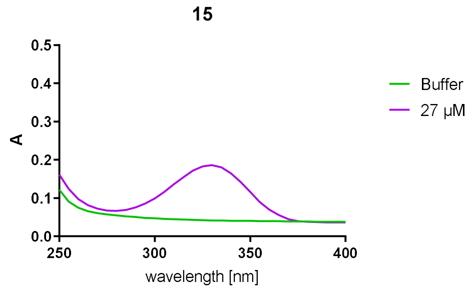

15

λ_{max} = 330 nm

Figure S4: Absorption spectra of a representative set of inhibitors with regards to the warhead λ_{max}. Recorded from 55 µM (27 µM for **15**) solutions in 5% DMSO/Tris buffer pH 7.4.

The absorption spectra show the expected absorption maximum > 250 nm that corresponds to the annealed heteroarenes. The range ≤ 250 nm was not evaluated due to the presence of DMSO, but the absorption maxima of the isolated aromatic systems (Phe, aryl-guanidines) are expected to be detected there.

The spectra change with identity or substitution pattern of the annealed system: benzothiophene has a lower-wavelength λ_{max} = 305 nm compared to benzothiazole (λ_{max} = 310 nm), while cyclohexyl thiazole shows λ_{max} = 330 nm. Halogen substitution of the benzothiazole has a weak bathochromic effect [H (λ_{max} = 310 nm) < F (λ_{max} = 315 nm) < Cl = Br (λ_{max} = 320 nm)], while methoxy substitution has a distinct bathochromic effect (λ_{max} = 350 nm).

Computation of physicochemical parameters.

Method: pK_a was calculated using MarvinJS 23.11.0 (https://playground.calculators.cxn.io/). Only the pK_a of the protonated guanidine moiety is reported, since it is the only relevant pH-responsive group in an aqueous medium. logP was calculated using molinspiration v2022.08 (https://www.molinspiration.com/cgi/properties), $logD_{7.4}$ was computed using MarvinJS 23.2.0 (https://plugins.calculators.cxn.io/logd/).

Table S1: List of compounds evaluated for some computed physicochemical properties (pK_A, logP, $logD_{7.4}$) sorted from most hydrophilic to most hydrophobic of the set.

compound	cpK_a (guanidine)	clogP	$clogD_{7.4}$
**MTX**	-	−2.0	−5.7
**15**	11.3	−2.2	−4.5
**14a**	11.7	−2.0	−4.5
**14e**	11.3	−1.9	−4.5
**14b**	11.2	−1.8	−4.5
**20**	11.7	−1.5	−4.2
**14f**	11.8	−0.9	−4.2
**14c**	11.2	−1.3	−4.0
**14d**	11.7	−1.2	−3.8
**38**	11.8	−0.8	−3.4
**45**	10.0	−0.7	−3.1
**29**	10.0	−0.6	−3.1
**39**	12.1	0.4	−2.9
**30**	10.0	0.6	−2.3
**31**	10.0	1.7	−0.9
**32**	10.0	2.7	−0.2
**33**	10.0	2.4	1.2
**PRO**	-	3.0	0.8

Stability studies.

Method: 10–20 mM stock solutions of inhibitors in DMSO were diluted into ACN/water or solutions in ACN/water were prepared directly from solid and then analyzed by LC/MS (“stock”). Alternatively, stock solutions were diluted into DMSO/buffer for PAMPA, incubated either for 7 h at room temperature or 17 h at 37 °C and then analyzed by LC/MS (“room temperature” or “37 °C”). Samples at room temperature were concentrated by lyophilization to ca. 10x the original concentration prior to analysis. The LC/MS method is the same as described in the PAMPA section as “measurement setup for LC/MS”. The chromatograms depicted in the results section are always in the following order: “stock”, then “room temperature”, then “37 °C”.

45

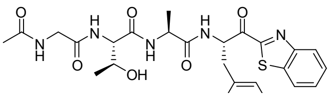

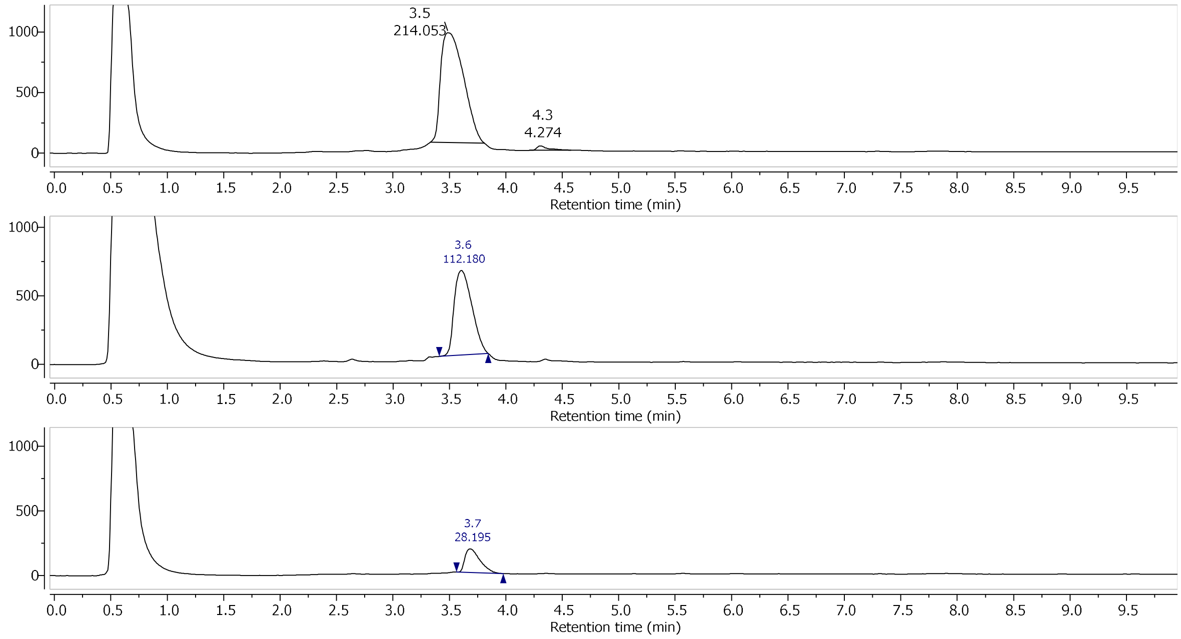

14e

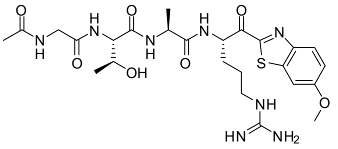

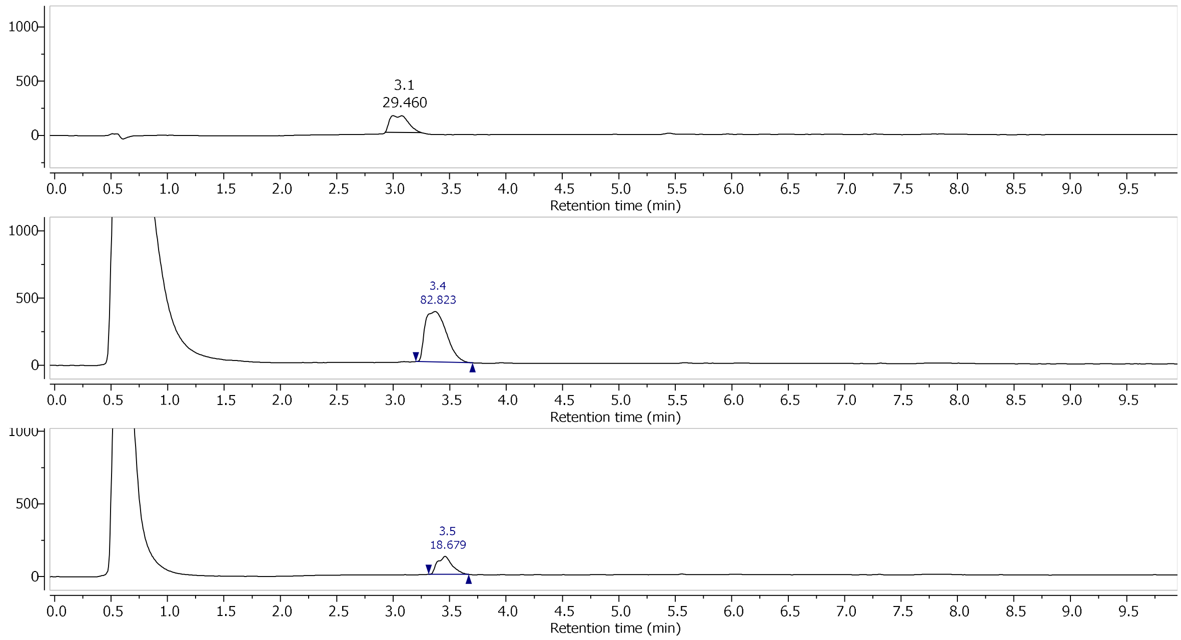
3.1
29.460
3.4
82.823
3.5
18.679
Retention time (min)

14b

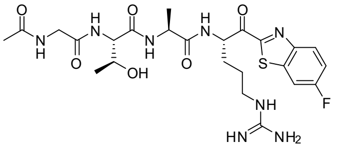

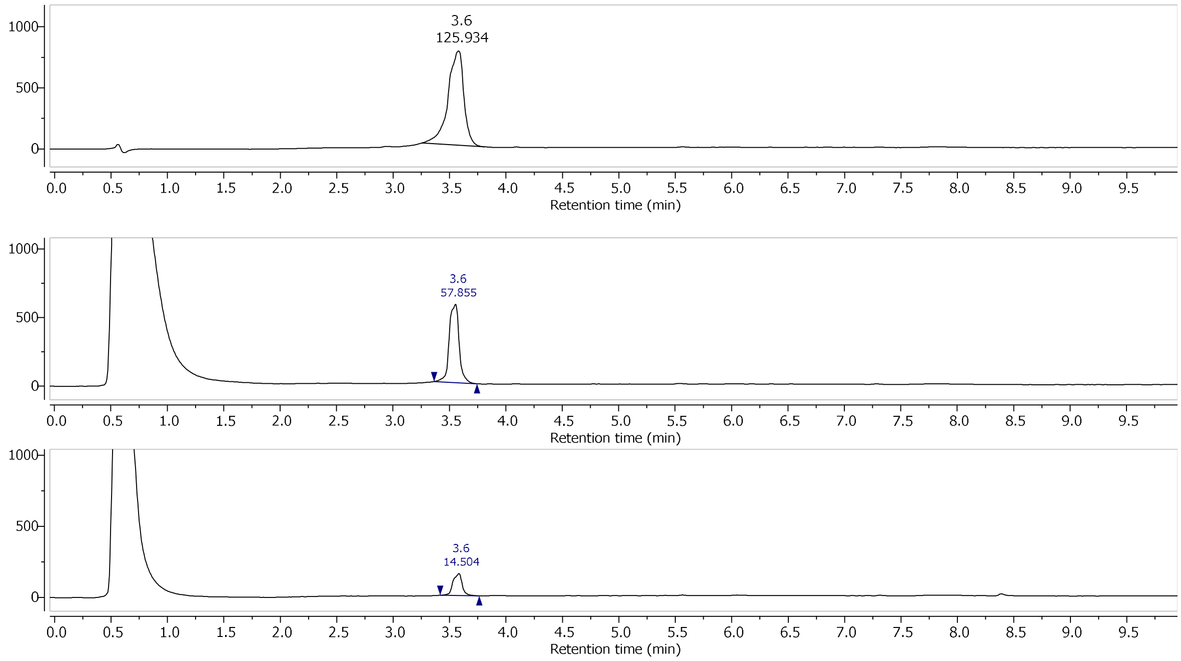
3.6
125.934
3.6
57.855
3.6
14.504
Retention time (min)

29

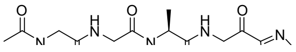

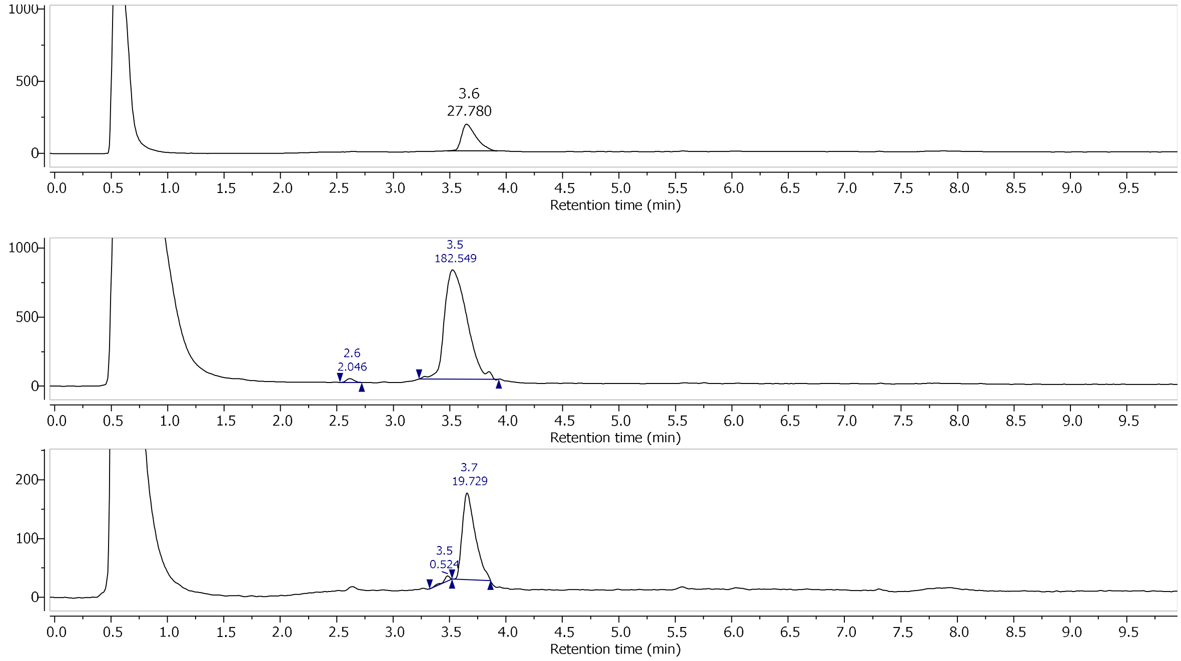

Figure S5: Stability analyses of a representative set of inhibitors. The depictions consist of compound structure and number, then analysis at t_0, then analysis after 7 h at room temperature, then analysis after 12 h at 37 °C.

Parallel Artificial Membrane Permeation Assay

The general principle reported in ref. [10] was used.

Compound solutions were diluted from 10–20 mM stock solutions in DMSO to 100–400 µM in a buffered (TRIS 50 mM pH 7.4) aqueous solution with 5% final content of DMSO (“donor solution”). A similarly prepared solution (buffer + 5% DMSO) was used as “acceptor solutions”. Incubation setup consisted of donor (top) plate (Sigma Aldrich, MAIPNTR10), 5 µL artificial membrane (1 % (w/v) L-α-phosphatidylcholine, Sigma Aldrich P3556, in *n*-dodecane, Sigma Aldrich 8205430100), acceptor (bottom) plate (Greiner, 655074). 150 µL of donor solution was applied onto the artificial membrane which was applied first to the donor plate. This upper compartment was sealed (Greiner, 676070, Viewseal sealer). 400 µL of acceptor solution was applied to the acceptor plate. Incubation setup was assembled and left for 7 h at room temperature. Reference solutions were prepared by simply mixing the indicated volumes of donor and acceptor solutions at the start of the incubation period and analyzed later with the acceptor solutions. The experiment was performed in duplicates.

Measurement setup for spectroscopy consisted of UV-transparent measurement plate (Greiner UV-Star®, 655801), Tecan Spark 10M® well plate reader, 200 µL sample volume, λ = 200–650 nm. Analysis of UV data was performed with AUC function in GraphPad Prism using the indicated wavelength range. Acceptor and reference spectra were baseline-corrected to buffer spectrum at a wavelength range without absorption (λ = 450–500 nm).

Measurement setup for LC/MS consisted of Agilent 1100 series HPLC system coupled to an Agilent 1100 series LC/MSD Trap with electron spray ionization (ESI). An Agilent Poroshell 120 EC-C18, 150x2.10 mm, 4 µm column was used. A linear gradient was used for elution with a ternary pump using [water/ACN/water + 0.1% formic acid] changing ratios from 80/10/10 to 0/90/10 over the course of 10 min (0.7 mL/min). Injection volume was 100 µL. Areas of peaks in the chromatogram (detection-λ = 210 nm) were used to calculate the AUCs. Retention time and mass spectrum recorded in positive ionization mode were used to assign species. Analysis was performed using Mestrenova 12.0.2.

Calculations of effective permeability P_e were performed using the following equation with V_D and V_A as volumes of donor and acceptor solutions (0.15 cm^3 and 0.4 cm^3, respectively), AUC_{acc} and AUC_{ref} as the area of the measured and baseline-corrected spectrum of acceptor and reference solutions after incubation, A as the porosity-corrected filter area (0.3019 cm^2*0.7 = 0.2113 cm^2) and t as the incubation time given in seconds.

$$P_e = -\frac{V_D * V_A * \ln\left(1 - \frac{AUC_{acc}}{AUC_{ref}}\right)}{(V_D + V_A) * A * t} \quad 4$$

Lit. values for propranolol (Lit-P_e(propranolol) = 8.3–13.9 *10^{-6} cm/s [11,12], measured P_e = 9.0 ± 0.2*10^{-6} cm/s) and methotrexate (Lit-P_e(methotrexate) = 0.0 *10^{-6} cm/s [13], measured P_e = 0*10^{-6} cm/s) were replicated ensuring reliability of data for novel compounds.

Synthesis

General Methods and Materials:

All reagents and solvents were purchased commercially and used as provided by the supplier without further purification. Solvents for synthesis, extraction, and chromatography were of analytical grade. Moisture-sensitive reactions were carried out under argon atmosphere as indicated, and anhydrous solvents were used as provided by the commercial supplier. Reaction progress was monitored by thin-layer chromatography using Alugram Xtra F254 silica plates from Macherey-Nagel and/or LC-MS (Agilent 1100 series HPLC system and an Agilent Poroshell 120 EC-C18, 150 x 2.10 mm, 4 μm column coupled to an Agilent 1100 series LC/MSD Trap with electron spray ionization (ESI)). The identities and purities of compounds were determined by the same LC-MS system with a gradient of acetonitrile and water (+0.1% formic acid). Signals were detected at 210/254 nm with quantitation by AUC and masses were determined in positive ionization mode (ESI). HPLC purification was performed with the Agilent 1290 II Infinity Preparative LC System using an InfinityLab Pursuit XRs C18, 30 x 250mm, 5 µm, preparative LC column and a gradient method (10% ACN (0.1% formic acid) to 100% ACN (0.1% formic acid)). Flash chromatography was performed with the Biotage Isolera™ One system using prepacked columns from Biotage. Silica gel (0.040 – 0.063 mm) from Macherey-Nagel was used for column chromatography. Optical rotations $[\alpha]_D^{20}$ were measured on an P3000 polarimeter from Krüss at 20 °C and are reported in $ml \cdot dm^{-1} \cdot g^{-1}$ with the concentration *c* being g/100 ml. Fourier-transformed ATR-corrected IR spectra were measured on an Avatar 330 single crystal spectrometer from ThermoNicolet. Melting points (uncorrected) were measured with an MPM-H3 using semi-open capillaries. NMR spectra were recorded as stated individually on Bruker Fourier 300 MHz, Bruker Avance DSX 400 MHz and Bruker Avance III 600 MHz. Chemical shifts are indicated in parts per million (ppm), with the solvent resonance ($CDCl_3$ or DMSO-d_6 from Deutero GmbH) as internal standard. The purity of all compounds tested in biological assays was ≥95% as determined by LC-MS.

Solid-Phase Peptide Synthesis (SPPS)[2]

A Resin loading. Solid-phase peptide synthesis was conducted in a fritted 12 mL polypropylene syringe. 1 g of 2-chlorotritylchloride resin (2-CTC resin, 1.2 mmol loading capacity) was pre-swelled in 8 mL DCM for 15 min and drained. The first amino acid (3.6 mmol, 3 eq. relative to resin loading capacity) was added in 1.8 M *N*-methylmorpholine (NMM)/DCM (6 mL) and the mixture was swiveled for 12 h. After draining the solution, the resin was washed with DMF (3 × 6 mL for 1 min) and DCM (3 × 6 mL for 1 min).

B Resin capping. The remaining free 2-CTC resin linkers were capped with MeOH in a solution of 9 mL of DCM/MeOH/DIPEA (9:2:1). The resin was swiveled for 1 h, drained, and washed with DCM (3 × 6 mL).

C Fmoc deprotection. The resin was treated with 20% piperidine in DMF solution (2 × 6 mL for 10 min) and subsequently washed with DMF (3 × 6 mL).

D Peptide coupling with HATU. A coupling cocktail was prepared including the specific Fmoc-protected amino acid (3.6 mmol, 3 eq.), HATU (3.6 mmol, 3 eq.), and DIEA (10.8 mmol, 9 eq.) in DMF (4.8 mL). The solution was added to the resin and swiveled for 3 h with exception of Fmoc-Arg(Pbf)-OH which was coupled overnight. After the reaction, the resin was drained and washed with DMF (3 × 6 mL for 1 min) and DCM (3 × 6 mL for 1 min).

E Acetylation of the *N*-terminus. The free amino terminus was acetylated using 8 mL of Ac_2O, DIEA and DCM (3:2:3). The reaction was shaken overnight and subsequently drained and washed with DMF (3 × 6 mL for 1 min) and DCM (3 × 6 mL for 1 min).

F Resin cleavage with preservation of side chain protecting groups. After the last coupling or acetylation step, the resin was washed with DMF (3 × 6 mL for 1 min) and DCM (3 × 6 mL for 1 min). Then a cleavage cocktail containing AcOH/trifluoroethanol/DCM (1:1:4, 6 mL) was added and swiveled for 1.5 h. The resin was drained and washed with DCM (5 mL). The combined eluates were concentrated under reduced pressure to yield the crude linear peptide. The purification of the crude product was performed by flash chromatography on a reversed-phase silica column (POLYPREP 60-50 C_{18}).

3, (*S*)-2-((2*S*,3*S*)-2-(2-Acetamidoacetamido)-3-(*tert*-butoxy)butanamido)propanoic acid

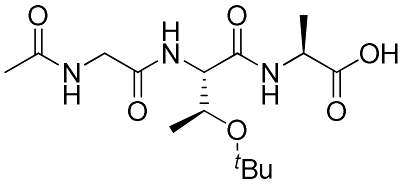

The title compound was prepared according to general procedures A, B, C, D, and F on 1.2 mmol scale. The product was purified by reversed phase flash chromatography (H_2O/MeCN, gradient) and obtained as a colorless solid after lyophilization (314 mg, 0.90 mmol, 75%) ^{1}H NMR (300 MHz, $CDCl_3$): δ/ppm = 8.19 (t, J = 5.8 Hz, 1H), 7.95 (d, J = 7.0 Hz, 1H), 7.53 (d, J = 8.5 Hz, 1H), 4.26 – 4.16 (m, 2H), 3.92 (qd, J = 6.2, 3.9 Hz, 2H), 3.74 (dd, J = 7.2, 5.8 Hz, 2H), 1.86 (s, 3H), 1.28 (d, J = 7.2 Hz, 3H), 1.12 (s, 9H), 1.01 (d, J = 6.2 Hz, 3H), ^{13}C NMR (75 MHz, CDCl3): δ/ppm = 173.8, 169.9, 169.3, 168.9, 73.8, 67.2, 57.3, 47.7, 42.3, 28.1, 22.4, 19.3, 17.5, FT-IR: ν/cm^{-1} = 668, 701, 1081, 1121, 1159, 1192, 1214, 1371, 1518, 1636, mp: 87 – 95 °C. $[\alpha]_D^{20}$ = +12 (*c* 1.00, $CHCl_3$), MS (ESI) *m/z* calculated for $[C_{15}H_{28}N_3O_6]^+$ ($[M+H]^+$): 346.2, found 368.1 ($[M+Na]^+$).

21, (*S*)-1-((*S*)-2-Acetamido-4-oxo-4-(tritylamino)butanoyl)pyrrolidine-2-carboxylic acid

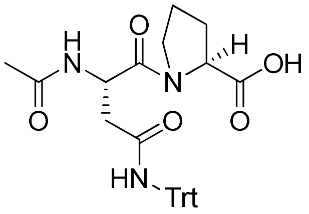

The title compound was prepared according to general procedures A, B, C, D, E and F on 1.2 mmol scale. The product was purified by reversed phase flash chromatography (H_2O/MeCN, gradient) and obtained as a colorless solid after lyophilization (326 mg, 0.63 mmol, 52%) ^{1}H NMR (300 MHz, $CDCl_3$): δ/ppm = 7.71 – 7.41 (m, 2H), 7.22 – 7.16 (m, 15H), 5.02 – 4.92 (m, 1H), 4.33 – 4.20 (m, 1H), 3.70 – 3.42 (m, 3H), 2.77 – 2.58 (m, 2H), 1.99 – 1.75 (m, 7H), ^{13}C NMR (75 MHz, CDCl3): δ/ppm = 170.5, 160.5, 154.9, 144.3, 128.9, 127.9, 127.0, 123.8, 70.9, 59.9, 58.1, 48.6, 47.6, 39.1, 31.1, 28.7, 24.9, 23.0, FT-IR: v/cm^{-1} = 689, 903, 1198, 1437, 1521, 1628, 1726, 2240, 3047, 3303, mp: 184 – 188 °C, $[\alpha]_D^{20}$ = −16 (*c* 1.00, $CHCl_3$), MS (ESI) *m/z* calculated for $[C_{30}H_{32}N_3O_5]^+$ ($[M+H]^+$): 513.5, found 536.1 ($[M+Na]^+$).

22, (*S*)-2-((*S*)-2-Acetamido-4-oxo-4-(tritylamino)butanamido)-3-phenylpropanoic acid

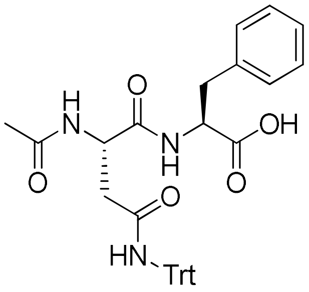

The title compound was prepared according to general procedures A, B, C, D, E and F on 1.2 mmol scale. The product was purified by reversed phase flash chromatography (H_2O/MeCN, gradient) and obtained as a colorless solid after lyophilization (514 mg, 0.91 mmol, 76%) ^{1}H NMR (300 MHz, DMSO-d_6): δ/ppm = 8.51 – 8.37 (m, 1H), 8.22 – 8.08 (m, 1H), 7.89 – 7.79 (m, 1H), 4.69 – 4.57 (m, 1H), 4.50 – 4.39 (m, 1H), 3.10 – 3.01 (m, 1H), 2.96 – 2.84 (m, 1H), 2.65 – 2.54 (m, 1H), 2.03 (s, 1H), 1.85 (s, 3H), ^{13}C NMR (75 MHz, DMSO-d_6): δ/ppm = 172.6, 171.2, 169.2, 168.7, 144.8, 137.3, 129.2, 129.1, 128.5, 128.2, 127.4, 126.5, 126.3, 69.3, 53.4, 49.8, 36.5, 22.5, FT-IR: v/cm^{-1} = 697, 748, 903, 1040, 1181, 1439, 1484, 1639, 3041, 3274, mp: 226 – 228 °C, $[\alpha]_D^{20}$ = −14 (*c* 1.00, DMSO), MS (ESI) *m/z* calculated for $[C_{34}H_{34}N_3O_5]^+$ ($[M+H]^+$): 563.6, found 586.1 ($[M+Na]^+$).

23, (*S*)-2-((*S*)-2-Acetamido-4-oxo-4-(tritylamino)butanamido)-3-cyclohexylpropanoic acid

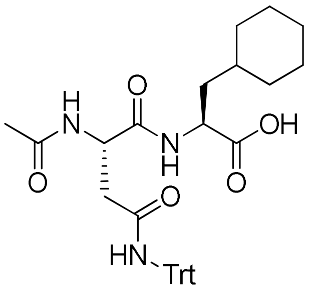

The title compound was prepared according to general procedures A, B, C, D, E and F on 1.2 mmol scale. The product was purified by reversed phase flash chromatography (H_2O/MeCN, gradient) and obtained as a colorless solid after lyophilization (467 mg, 0.82 mmol, 68%) ^{1}H NMR (300 MHz, $CDCl_3$): δ/ppm = 7.61 – 7.44 (m, 2H), 7.28

– 7.14 (m, 15H), 4.90 – 4.67 (m, 2H), 4.40 – 4.21 (m, 1H), 2.93 – 2.54 (m, 2H), 1.87 (s, 3H), 1.71 – 1.04 (m, 12H), 0.85 (q, *J* = 11.3 Hz, 2H), ^{13}C NMR (75 MHz, CDCl3): *δ*/ppm = 175.4, 171.7, 171.5, 171.3, 171.1, 144.3, 144.1, 128.8, 128.0, 127.1, 70.9, 50.9, 50.1, 38.6, 37.7, 34.1, 33.4, 32.4, 26.4, 26.1, 26.0, 23.0, FT-IR: *v*/cm^{-1} = 699, 736, 914, 1204, 1435, 1510, 1656, 1722, 2365, 2926, mp: 180 – 182 °C, $[\alpha]_D^{20}$ = −15 (*c* 1.00, $CHCl_3$), MS (ESI) *m/z* calculated for $[C_{34}H_{40}N_3O_5]^+$ ($[M+H]^+$): 569.6, found 592.1 ($[M+Na]^+$).

Synthesis of P1-precursor molecules

5, *Tert*-Butyl (*S*)-(1-(methoxy(methyl)amino)-1-oxo-5-(3-((2,2,4,6,7-pentamethyl-2,3-dihydrobenzofuran-5-yl)sulfonyl)guanidino)pentan-2-yl)carbamate

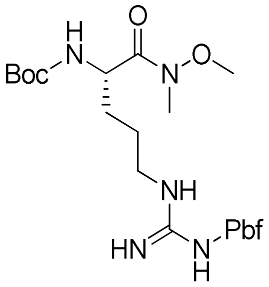

(Adapted from St-Georges *et al.*)[14]

Boc-Arg(Pbf)-OH **4** (4.00 g, 7.59 mmol, 1 eq) was dissolved in DCM (30 mL) under argon atmosphere. At 0 °C DIEA (10.58 mL, 60.76 mmol, 8 eq) and TBTU (2.92 g, 9.11 mmol, 1.2 eq) were added. The reaction mixture was stirred for 30 min at 0 °C, *N*,*O*-dimethyl hydroxylamine · HCl (4.44 g, 45.54 mmol, 6 eq) was added and stirred for 12 h. The solution was diluted with DCM (15 mL) and washed with saturated $NaHCO_3$ solution (3x 20 mL) and saturated NaCl solution (3x 20 mL). The organic solution was dried over anhydrous Na_2SO_4 and evaporated under reduced pressure. The crude product was purified by column chromatography (EA) to yield the desired product as a colorless solid (3.89 g, 6.83 mmol, 90%). ^{1}H NMR (300 MHz, CDCl3): *δ*/ppm = 6.38 (s, 1H), 5.49 (d, *J* = 8.8 Hz, 1H), 4.61 (s, 1H), 3.73 (s, 3H), 3.18 (s, 3H), 2.95 (s, 3H), 2.56 (s, 3H), 2.50 (s, 3H), 2.08 (s, 3H), 1.74 – 1.52 (m, 4H), 1.45 (s, 6H), 1.41 (s, 9H), ^{13}C NMR (75 MHz, CDCl3): *δ*/ppm = 156.4, 156.0, 138.8, 132.7, 124.8, 117.7, 86.6, 80.3, 61.7, 43.3, 41.0, 30.9, 28.7, 28.4, 24.9, 19.4, 18.0, 12.5, mp: 76 – 84 °C. $[\alpha]_D^{20}$ = −23 (*c* 1.00, $CHCl_3$). FT-IR: *v*/cm^{-1} = 668, 756, 1106, 1165, 1456, 1556, 1652, 2362, 2974, 2981. MS (ESI) *m/z* calculated for $[C_{26}H_{44}N_5O_7S]^+$ ($[M+H]^+$): 570.3, found 570.3.

12a, *Tert*-Butyl (*S*/*R*)-(1-(benzo[*d*]thiazol-2-yl)-1-oxo-5-(3-((2,2,4,6,7-pentamethyl-2,3-dihydrobenzofuran-5-yl)sulfonyl)guanidino)pentan-2-yl)carbamate

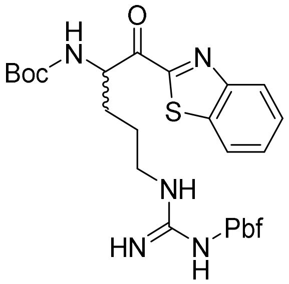

(Adapted from Costanzo *et al.*)[15]

Benzothiazole **10a** (4.83 mL, 44.67 mmol, 10.2 eq) was dissolved in dry THF (80 mL) under argon atmosphere and cooled to −78 °C. *n*-BuLi (2.5 M in hexanes, 17.52 mL, 43.80 mmol, 10 eq) was added dropwise and the reaction stirred for 1.5 h at the same temperature. Afterwards, **5** (2.50 g, 4.38 mmol, 1 eq) was dissolved in dry THF (30 mL), added slowly to the reaction mixture and stirred for two hours at −78 °C. The reaction mixture was

quenched with saturated aqueous NH_4Cl solution (20 mL). The organic phase was separated and washed three times each with saturated $NaHCO_3$ solution (25 mL) and saturated NaCl solution (25 mL). The organic phase was dried over anhydrous $NaSO_4$ and concentrated under reduced pressure. The crude product was purified by column chromatography (CH/EA, 1:1 to 0:1) to yield the product as a colorless solid (1.11 g, 3.06 mmol, 70%). ^{1}H NMR (300 MHz, $CDCl_3$): δ/ppm = 8.23 – 8.15 (m, 1H), 8.00 – 7.91 (m, 1H), 7.60 – 7.49 (m, 2H), 6.31 (s, 1H), 6.21 (s, 2H), 5.65 (d, J = 8.6 Hz, 1H), 5.61 – 5.49 (m, 1H), 3.57 – 3.18 (m, 2H), 2.92 (s, 3H), 2.54 (s, 3H), 2.49 (s, 3H), 2.06 (s, 3H), 1.72 (s, 4H), 1.45 (s, 6H), 1.41 (s, 9H), ^{13}C NMR (75 MHz, $CDCl_3$): δ/ppm = 158.8, 156.2, 153.5, 138.5, 137.3, 133.0, 132.5, 128.3, 127.4, 126.0, 124.7, 122.5, 117.5, 86.4, 80.6, 43.3, 40.8, 28.7, 28.4, 25.5, 19.3, 18.0, 12.5, FT-IR: ν/cm^{-1} = 668, 756, 1102, 1164, 1216, 1369, 1483, 1552, 1622, 1694, mp: 105 – 115 °C. $[\alpha]_D^{20}$ = +6 (c 1.00, $CHCl_3$, MS (ESI) m/z calculated for $[C_{31}H_{42}N_5O_6S_2]^+$ ($[M+H]^+$): 644.3, found 644.3.

12f, *Tert*-Butyl (*S*/*R*)-(1-(benzo[*b*]thiophene-2-yl)-1-oxo-5-(3-((2,2,4,6,7-pentamethyl-2,3-dihydrobenzofuran-5-yl)sulfonyl)guanidino)pentan-2-yl)carbamate

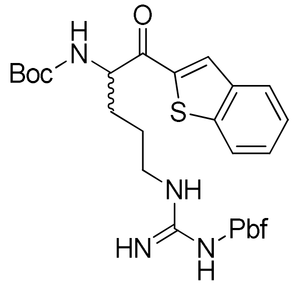

(Adapted from Costanzo *et al.*)[15]

1-Benzothiophene **10f** (0.71 g, 5.30 mmol, 10 eq) was dissolved in dry THF (20 mL) under argon atmosphere and cooled to −78 °C. *n*-BuLi (2.5 M in hexanes, 1.70 mL, 4.24 mmol, 8 eq) was added dropwise and the reaction stirred for 2 h letting the temperature rise to rt. Afterwards, **5** (0.30 g, 0.53 mmol, 1 eq) was dissolved in dry THF (10 mL), added slowly to the again cooled reaction mixture at −78 °C and stirred for 2.5 h. The reaction mixture was quenched with saturated NH_4Cl solution (5 mL). The organic phase was separated and washed three times each with saturated $NaHCO_3$ solution (10 mL) and saturated NaCl solution (10 mL). The organic phase was dried over anhydrous $NaSO_4$ and concentrated under reduced pressure. The crude product was purified by column chromatography (CH/EA, 1:1 to 1:3) to yield the product as a yellow solid (0.27 g, 0.42 mmol, 80%). ^{1}H NMR (300 MHz, DMSO-d_6): δ/ppm = 8.41 (s, 1H), 8.31 (s, 1H), 8.08–7.99 (m, 2H), 7.58–7.44 (m, 3H), 4.92 (s, 1H), 3.12–3.02 (m, 2H), 2.90 (s, 2H), 2.45 (s, 3H), 2.39 (s, 3H), 1.96 (s, 3H), 1.79–1.48 (m, 5H), 1.42–1.32 (m, 15H), ^{13}C NMR (75 MHz, DMSO-d_6): δ/ppm = 194.3, 157.4, 156.1, 155.5, 141.4, 141.2, 139.1, 137.2, 134.1, 131.4, 130.5, 127.8, 126.4, 125.3, 124.3, 123.1, 116.2, 86.2, 79.2, 78.4, 55.8, 40.4, 28.3, 28.1, 18.9, 17.5, 12.2, FT-IR: ν/cm^{-1} = 667, 763, 816, 1024, 1097, 1148, 1252, 1561, 2966, 3334, mp: 105 – 126 °C. $[\alpha]_D^{20}$ = +7 (c 1.00, DMSO), MS (ESI) m/z calculated for $[C_{32}H_{42}N_4O_6S_2]^+$ ($[M+H]^+$): 643.3, found 643.2.

9, 2-Amino-4,5,6,7-tetrahydrobenzothiazole

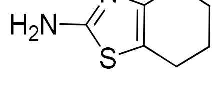

(Adapted from Furlan *et al.*)[16]

Cyclohexane **7** (3 g, 30.6 mmol, 1 eq), thiourea **8** (4.65 g, 61.2 mmol, 2 eq) and iodine (7.74 g, 30.6 mmol, 1 eq) were stirred at 130 °C under argon atmosphere for 12 h. To the cooled mixture was added water (50 mL) and stirred for 30 min. The aqueous phase was washed three times with ether (50 mL) and neutralized with $NaHCO_3$ (pH = 7). The precipitate was filtered and solved in hot saturated Na_2CO_3 solution. The cooled mixture was extracted with DCM (30 mL) dried over Na_2SO_4 and evaporated under reduced pressure to yield the product as

a yellowish solid (2.5 g, 16.2 mmol, 53%). ^{1}H NMR (300 MHz, DMSO-d_6): δ/ppm = δ 6.56 (s, 2H), 2.51 – 2.41 (m, 2H), 2.39 – 2.30 (m, 2H), 1.76 – 1.63 (m, 4H), ^{13}C NMR (75 MHz, DMSO-d_6): δ/ppm = 165.3, 144.7, 114.4, 26.2, 23.2, 22.7, 22.5, FT-IR: v/cm^{-1} = 692, 892, 1018, 1062, 1112, 1236, 1309, 1365, 1524, 1634, mp: 86 – 90 °C, MS (ESI) *m/z* calculated for $[C_7H_{13}N_2S]^+$ ([M+H]$^+$): 155.0., found 155.9.

General procedure for desamination of the 2-aminobenzoheterocycle derivatives

(Adapted from Capaldo *et al.*)[17]

The respective 2-aminobenzoheterocycle **6b**-**e**, **9** (1 eq) was dissolved in dry THF (25 mL) under argon atmosphere. Isopentyl nitrite (2.2 eq) was added dropwise under light protection and the reaction stirred for 2 – 5 h under reflux. Afterwards, ice-water was added (40 mL) and the aqueous solution was extracted three times with EA (30 mL). The organic phase was dried over anhydrous $NaSO_4$ and concentrated under reduced pressure.

10e, 6-Methoxybenzo[*d*]thiazole

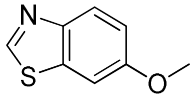

The crude product was purified by column chromatography (CH/EA, 20:1) to yield the product as a yellow solid (1.98 g, 11.96 mmol, 72%). ^{1}H NMR (300 MHz, $CDCl_3$): δ/ppm = 8.84 (s, 1H), 8.01 (dd, J = 9.0, 0.5 Hz, 1H), 7.38 (d, J = 2.5 Hz, 1H), 7.12 (dd, J = 9.0, 2.5 Hz, 1H), 3.87 (s, 3H), ^{13}C NMR (75 MHz, $CDCl_3$): δ/ppm = 158.2, 151.6, 147.5, 135.1, 124.0, 116.1, 104.1, 55.9, FT-IR: v/cm^{-1} = 653, 822, 900, 1018, 1049, 1193, 1246, 1476, 1608, 2952, mp: 72 – 75 °C, MS (ESI) *m/z* calculated for $[C_8H_7NOS]^+$ ([M+H]$^+$): 166.0, found 165.9.

10b, 6-Fluorobenzo[*d*]thiazole

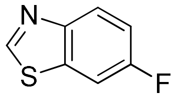

The crude product was purified by column chromatography (CH/EA, 20:1) to yield the product as a yellow solid (1.99 g, 12.96 mmol, 66%). ^{1}H NMR (300 MHz, $CDCl_3$): δ/ppm = 8.96 (s, 1H), 8.08 (dd, J = 9.0, 4.8 Hz, 1H), 7.62 (dd, J = 8.1, 2.5 Hz, 1H), 7.26 (td, J = 8.9, 2.5 Hz, 1H), ^{13}C NMR (75 MHz, $CDCl_3$): δ/ppm = 162.5, 159.3, 149.8, 134.9, 124.6, 115.2, 108.1, FT-IR: v/cm^{-1} = 779, 807, 838, 971, 1218, 1260, 1451, 1482, 1569, 3081, mp: 57 – 61 °C, MS (ESI) *m/z* calculated for $[C_7H_4FNS]^+$ ([M+H]$^+$): 154.0, found 153.9.

10c, 6-Chlorobenzo[*d*]thiazole

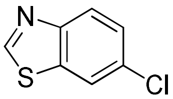

The crude product was purified by column chromatography (CH/EA, 20:1) to yield the product as a yellow solid (1.26 g, 7.43 mmol, 46%). ^{1}H NMR (300 MHz, $CDCl_3$): δ/ppm = 9.00 (s, 1H), 8.05 (dd, J = 8.7, 0.5 Hz, 1H), 7.93 (dd, J = 2.1, 0.5 Hz, 1H), 7.48 (dd, J = 8.7, 2.1 Hz, 1H), ^{13}C NMR (75 MHz, $CDCl_3$): δ/ppm = 154.5, 151.6, 135.0, 131.9, 127.3, 124.4, 121.6, FT-IR: v/cm^{-1} = 757, 802, 855, 889, 1046, 1108, 1392, 1426, 1474, 3042, mp: 42 – 47 °C, MS (ESI) *m/z* calculated for $[C_7H_4ClNS]^+$ ([M+H]$^+$): 170.0, found 169.9.

10d, 6-Bromobenzo[*d*]thiazole

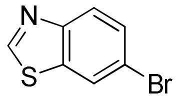

The crude product was purified by column chromatography (CH/EA, 30:1) to yield the product as a yellow solid (2.14 g, 9.99 mmol, 76%). ^{1}H NMR (300 MHz, $CDCl_3$): δ/ppm = 9.01 (s, 1H), 8.10 (dd, *J* = 1.9, 0.5 Hz, 1H), 8.00 (dd, *J* = 8.7, 0.5 Hz, 1H), 7.63 (dd, *J* = 8.7, 1.9 Hz, 1H), ^{13}C NMR (75 MHz, $CDCl_3$): δ/ppm = 154.6, 151.8, 135.5, 130.0, 124.7, 124.6, 119.7, FT-IR: v/cm^{-1} = 740, 799, 858, 886, 1077, 1294, 1386, 1459, 1547, 3039, mp: 50 – 55 °C, MS (ESI) *m/z* calculated for $[C_7H_4BrNS]^+$ ($[M+H]^+$): 213.9, found 213.8.

11, 4,5,6,7-Tetrahydrobenzothiazole

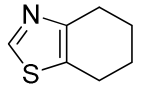

The crude product was purified by column chromatography (CH/EA, 7:1) to yield the product as a yellow oil (1.08 g, 9.99 mmol, 76%). ^{1}H NMR (300 MHz, $CDCl_3$): δ/ppm = 8.60 (s, 1H), 2.87 – 2.74 (m, 4H), 1.90 – 1.80 (m, 4H), ^{13}C NMR (75 MHz, $CDCl_3$): δ/ppm = 150.8, 149.6, 128.8, 26.7, 23.5, 22.9, FT-IR: v/cm^{-1} = 712, 807, 859, 891, 1138, 1209, 1379, 1413, 1458, 1591, MS (ESI) *m/z* calculated for $[C_7H_9NS]^+$ ($[M+H]^+$): 140.05, found 139.9.

12e, *Tert*-butyl (*S*/*R*)-(1-(6-methoxybenzo[d]thiazol-2-yl)-1-oxo-5-(3-((2,2,4,5,7-pentamethyl-2,3-dihydrobenzofuran-6-yl)sulfonyl)guanidino)pentan-2-yl)carbamate

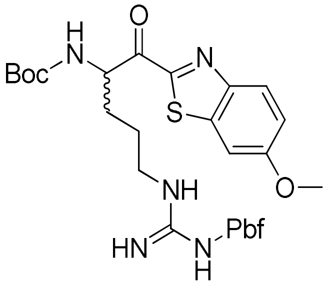

(Adapted from Costanzo *et al.*)[15]

6-methoxybenzo[*d*]thiazole **10e** (1.94 g, 11.72 mmol, 10.2 eq) was dissolved in dry THF (40 mL) under argon atmosphere and cooled to −78 °C. *n*-BuLi (2.5 M in hexanes, 4.60 mL, 11.50 mmol, 10 eq) was added dropwise and the reaction stirred for 2 h at the same temperature. Afterwards, **5** (0.66 g, 1.15 mmol, 1 eq) was dissolved in dry THF (20 mL), added slowly to the reaction mixture at −78 °C and stirred for 2 h. The reaction was quenched with saturated NH_4Cl solution (15 mL). The organic phase was separated and washed three times each with saturated $NaHCO_3$ solution (20 mL) and saturated NaCl solution (20 mL). The organic phase was dried over anhydrous $NaSO_4$ and concentrated under reduced pressure. The crude product was purified by column chromatography ($CHCl_3$/MeOH, 100:1) to yield the product as a brown oil (0.68 g, 1.01 mmol, 88%). ^{1}H NMR (300 MHz, $CDCl_3$): δ/ppm = 8.04 (d, *J* = 9.1 Hz, 1H), 7.34 (d, *J* = 2.5 Hz, 1H), 7.15 (dd, *J* = 9.1, 2.5 Hz, 1H), 6.52 (s, 2H), 5.68 (d, *J* = 8.6 Hz, 1H), 5.52 (s, 1H), 3.90 (s, 3H), 3.53 (s, 1H), 3.35–3.23 (m, 1H), 2.93 (s, 2H), 2.54 (s, 3H), 2.49 (s, 3H), 2.06 (s, 3H), 1.83–1.58 (m, 5H), 1.45 (s, 6H), 1.41 (s, 9H), ^{13}C NMR (75 MHz, $CDCl_3$): δ/ppm = 193.0, 160.3, 159.3, 156.5, 156.0, 152.4, 148.2, 139.6, 139.0, 133.0, 131.9, 126.8, 124.9, 118.2, 117.8, 103.7, 86.7, 80.6, 56.0, 55.1, 43.3, 40.9, 31.6, 28.7, 28.5, 25.4, 19.4, 18.0, 12.6, FT-IR: v/cm^{-1} = 723, 903, 1091, 1164, 1260, 1485, 1555, 1682, 2974, 3334, $[\alpha]_D^{20}$ = +9 (*c* 1.00, $CHCl_3$), MS (ESI) *m/z* calculated for $[C_{32}H_{44}N_5O_7S_2]^+$ ($[M+H]^+$): 674.3, found 674.2.

12b, *Tert*-butyl (*S*/*R*)-(1-(6-fluorobenzo[d]thiazol-2-yl)-1-oxo-5-(3-((2,2,4,5,7-pentamethyl-2,3-dihydrobenzofuran-6-yl)sulfonyl)guanidino)pentan-2-yl)carbamate

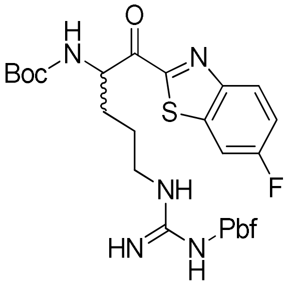

(Adapted from Costanzo *et al.*)[15]

6-Fluorobenzo[*d*]thiazole **10b** (1.99 g, 12.96 mmol, 10.2 eq) was dissolved in dry THF (40 mL) under argon atmosphere and cooled to −78 °C. *n*-BuLi (2.5 M in hexanes, 5.10 mL, 12.70 mmol, 10 eq) was added dropwise and the reaction stirred for 1 h under the same temperature. Afterwards, **5** (0.72 g, 1.27 mmol, 1 eq) was dissolved in dry THF (20 mL), added slowly to the reaction mixture at −78 °C and stirred for 2 hours. The reaction was quenched with saturated NH_4Cl solution (15 mL). The organic phase was separated and washed three times each with saturated $NaHCO_3$ solution (20 mL) and saturated NaCl solution (20 mL). The organic phase was dried over anhydrous $NaSO_4$ and concentrated under reduced pressure. The crude product was purified by column chromatography ($CHCl_3$/MeOH, 90:1) to yield the product as a yellow oil (0.46 g, 0.69 mmol, 54%). ^{1}H NMR (300 MHz, $CDCl_3$): δ/ppm = 8.16 (dd, *J* = 9.1 Hz, 4.8 Hz, 1H), 7.62 (dd, *J* = 7.9 Hz, 2.5 Hz, 1H), 7.34–7.27 (m, 1H), 6.58 (s, 2H), 5.67 (d, *J* = 8.5 Hz, 1H), 5.52 (s, 1H), 3.50 (s, 1H), 3.35–3.23 (m, 1H), 2.93 (s, 2H), 2.53 (s, 3H), 2.48 (s, 3H), 2.06 (s, 3H), 1.80–1.62 (m, 4H), 1.46 (s, 6H), 1.41 (s, 9H), ^{13}C NMR (75 MHz, $CDCl_3$): δ/ppm = 193.0, 164.0, 160.7, 159.4, 156.4, 155.9, 150.3, 138.7, 138.6, 133.0, 131.1, 127.4, 125.0, 117.8, 116.9, 108.4, 86.8, 80.7, 55.3, 43.3, 40.9, 28.7, 28.5, 25.4, 19.5, 18.1, 12.6, FT-IR: ν/cm^{-1} = 732, 909, 1091, 1162, 1260, 1488, 1561, 1693, 2971, 3334, $[\alpha]_D^{20}$ = +7 (*c* 1.00, $CHCl_3$), MS (ESI) *m/z* calculated for $[C_{31}H_{41}FN_5O_6S_2]^+$ ($[M+H]^+$): 662.2, found 662.2.

12c, *Tert*-butyl (*S*/*R*)-(1-(6-chlorobenzo[d]thiazol-2-yl)-1-oxo-5-(3-((2,2,4,5,7-pentamethyl-2,3-dihydrobenzofuran-6-yl)sulfonyl)guanidino)pentan-2-yl)carbamate

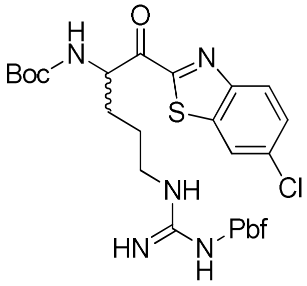

(Adapted from Costanzo *et al.*)[15]

6-Chlorobenzo[*d*]thiazole **10c** (1.23 g, 7.25 mmol, 10 eq) was dissolved in dry THF (25 mL) under argon atmosphere and cooled to −78 °C. *n*-BuLi (2.5 M in hexanes, 2.9 mL, 7.10 mmol, 10 eq) was added dropwise and the reaction stirred for 1 h under the same temperature. Afterwards, **5** (0.41 g, 0.71 mmol, 1 eq) was dissolved in dry THF (5 mL), added slowly to the reaction mixture at −78 °C and stirred for 2 hours. The reaction was quenched with saturated NH_4Cl solution (5 mL). The organic phase was separated and washed three times each with saturated $NaHCO_3$ solution (10 mL) and saturated NaCl solution (10 mL). The organic phase was dried over anhydrous $NaSO_4$ and concentrated under reduced pressure. The crude product was purified by column chromatography ($CHCl_3$/MeOH, 90:1) to yield the product as a yellow oil (0.27 g, 0.40 mmol, 55%). ^{1}H NMR (300 MHz, $CDCl_3$): δ/ppm = 8.10 (d, *J* = 8.8 Hz, 1H), 7.93 (d, *J* = 2.0 Hz, 1H), 7.50 (dd, *J* = 8.8, 2.1 Hz, 1H), 6.47 (s, 2H), 5.65 (d, *J* = 8.5 Hz, 1H), 5.51 (s, 1H), 3.47 (s, 1H), 3.34–3.22 (m, 1H), 2.93 (s, 2H), 2.53 (s, 3H), 2.47 (s, 3H), 2.06 (s, 3H), 1.81–1.61 (m, 4H), 1.46 (s, 6H), 1.41 (s, 9H), ^{13}C NMR (75 MHz, $CDCl_3$): δ/ppm = 219.5, 193.2, 159.3,

156.3, 156.1, 152.1, 138.9, 138.4, 134.7, 132.8, 131.8,128.5, 126.8, 124.9, 122.0, 117.8, 86.7, 80.7, 55.5, 43.3, 40.8, 28.7, 28.5, 25.5, 19.4, 18.0, 12.6, FT-IR: *v*/cm^{-1} = 726, 911, 1097, 1162, 1252, 1482, 1552, 1696, 2974, 3337, , $[\alpha]_D^{20}$ = +5 (*c* 1.00, $CHCl_3$), MS (ESI) *m/z* calculated for $[C_{31}H_{41}ClN_5O_6S_2]^+$ ($[M+H]^+$): 678.2, found 678.2.

12d, *Tert*-butyl (*S*/*R*)-(1-(6-bromobenzo[d]thiazol-2-yl)-1-oxo-5-(3-((2,2,4,5,7-pentamethyl-2,3-dihydrobenzofuran-6-yl)sulfonyl)guanidino)pentan-2-yl)carbamate

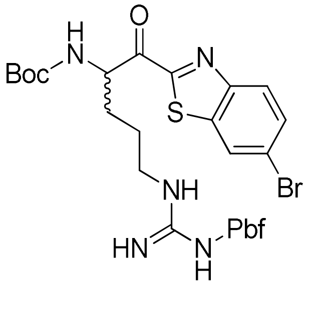

(Adapted from Costanzo *et al.*)[15]

6-Bromobenzo[*d*]thiazole **10d** (2.11 g, 9.9 mmol, 10.1 eq) was dissolved in dry THF (30 mL) under argon atmosphere and cooled to −78 °C. *n*-BuLi (2.5 m in hexanes, 3.9 mL, 9.70 mmol, 10 eq) was added dropwise and the reaction stirred for 1.5 h under the same temperature. Afterwards, **5** (0.55 g, 0.97 mmol, 1 eq) was dissolved in dry THF (10 mL), added slowly to the reaction mixture at −78 °C and stirred for 2 hours. The reaction was quenched with saturated NH_4Cl solution (5 mL). The organic phase was separated and washed three times each with saturated $NaHCO_3$ solution (10 mL) and saturated NaCl solution (10 mL). The organic phase was dried over anhydrous $NaSO_4$ and concentrated under reduced pressure. The crude product was purified by column chromatography ($CHCl_3$/MeOH, 70:1) to yield the product as a yellow oil (0.51 g, 0.51 mmol, 73%). ^{1}H NMR (300 MHz, $CDCl_3$): *δ*/ppm = 8.23–7.88 (m, 3H), 7.58–7.49 (m, 1H), 6.44 (s, 2H), 5.67 (t, *J* = 8.8 Hz, 1H), 5.50 (s, 1H), 3.42 (s, 1H), 3.32–3.20 (m, 1H), 2.92 (s, 2H), 2.52 (s, 3H), 2.46 (d, *J* = 2.1 Hz, 3H), 2.05 (s, 3H), 1.82–1.58 (m, 4H), 1.45 (s, 6H), 1.40 (s, 9H), ^{13}C NMR (75 MHz, $CDCl_3$): *δ*/ppm = 217.9, 193.4, 159.1, 156.2, 153.5, 152.3, 138.8, 137.3, 132.6, 131.1, 128.3, 127.4, 127.0, 126.0, 125.0, 124.8, 122.5, 117.7, 86.6, 80.6, 55.6, 43.3, 40.8, 28.7, 28.4, 25.5, 19.4, 18.1, 12.6, FT-IR: *v*/cm^{-1} = 723, 807, 886, 1097, 1246, 1398, 1459, 1558, 1690, 3042, $[\alpha]_D^{20}$ = +9 (*c* 1.00, $CHCl_3$), MS (ESI) *m/z* calculated for $[C_{31}H_{41}BrN_5O_6S_2]^+$ ($[M+H]^+$): 722.2, found 722.1.

13, *Tert*-butyl (*S*/*R*)-(1-oxo-5-(3-((2,2,4,5,7-pentamethyl-2,3-dihydrobenzofuran-6-yl)sulfonyl)guanidino)-1-(4,5,6,7-tetrahydrobenzo[d]thiazol-2-yl)pentan-2-yl)carbamate

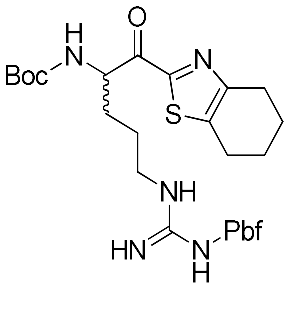

(Adapted from Costanzo *et al.*)[15]

4,5,6,7-Tetrahydrobenzothiazole **11** (0.75 g, 5.40 mmol, 10.1 eq) was dissolved in dry THF (18 mL) under argon atmosphere and cooled to −78 °C. TMEDA (0.79 mL, 5.28 mmol, 10 eq) and *n*-BuLi (2.5 m in hexanes, 2.1 mL, 5.28 mmol, 10 eq) were added dropwise and the reaction stirred for 1.5 h under the same temperature. Afterwards, **5** (0.30 g, 0.52 mmol, 1 eq) was dissolved in dry THF (8 mL), added slowly to the reaction mixture at −78 °C and stirred for 2 hours. The reaction was quenched with saturated NH_4Cl solution (5 mL). The organic phase was separated and washed three times each with saturated $NaHCO_3$ solution (10 mL) and saturated NaCl

solution (10 mL). The organic phase was dried over anhydrous $NaSO_4$ and concentrated under reduced pressure. The crude product was purified by column chromatography ($CHCl_3$/MeOH, 40:1) to yield the product as a colorless oil (0.17 g, 0.26 mmol, 50%). ^{1}H NMR (300 MHz, $CDCl_3$): δ/ppm = 7.28 (d, J = 7.4 Hz, 1H), 5.05 (s, 1H), 3.02 (d, J = 5.9 Hz, 2H), 2.95 (s, 2H), 2.87 (s, 2H), 2.78 (d, J = 6.1 Hz, 2H), 2.46 (s, 3H), 2.41 (s, 3H), 1.99 (s, 3H), 1.84 – 1.47 (m, 8H), 1.40 (s, 6H), 1.35 (s, 9H), ^{13}C NMR (75 MHz, $CDCl_3$): δ/ppm = 192.4, 160.5, 157.4, 156.0, 155.4, 153.2, 138.6, 137.2, 134.1, 131.4, 124.2, 116.2, 86.2, 78.2, 55.2, 42.4, 28.1, 26.4, 23.6, 22.4, 22.1, 18.9, 17.5, 12.2, FT-IR: v/cm^{-1} = 623, 812, 1118, 1164, 1249, 1366, 1425, 1540, 1677, 1719, $[\alpha]_D^{20}$ = +5 (c 1.00, $CHCl_3$), MS (ESI) m/z calculated for $[C_{31}H_{46}N_5O_6S_2]^+$ ($[M+H]^+$): 648.2, found 648.2.

17, (*S*)-6-(2,3-Bis((benzyloxy)carbonyl)guanidino)-2-((*tert*-butoxycarbonyl)amino)hexanoic acid

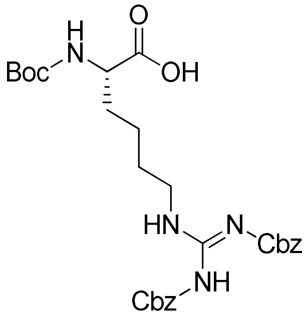

Boc-Lys-OH **16** (2.00 g, 8.12 mmol, 1 eq) and N,N'-bis-(carbobenzoxy)-1H-pyrazole-1-carboxamidine (3.38 , 8.93 mmol, 1.1 eq) were dissolved in DMF (58 mL) and Et_3N (1.69 mL, 12.18 mmol, 1.5 eq) was added under argon atmosphere. The reaction stirred for 72 h and got quenched by addition of NH_4Cl (20 mL). The mixture was extracted with EA (50 mL), dried over $MgSO_4$ and the organic solvents were removed under reduced pressure. The crude product was purified by column chromatography (CH/EA, 2:1 + 0.1% TFA) to yield the product as a colorless oil (4.94 g, 8.12 mmol, quant.). ^{1}H NMR (300 MHz, CDCl3): δ/ppm = 7.40 – 7.28 (m, 10H), 5.18 (d, J = 16.0 Hz, 4H), 4.27 (s, 1H), 3.47 (s, 2H), 1.92 – 1.56 (m, 6H), 1.43 (s, 9H), ^{13}C NMR (75 MHz, CDCl3): δ/ppm = 175.6, 155.2, 153.7, 135.7, 134.2, 129.1, 128.9, 128.7, 128.6, 128.4, 128.3, 69.0, 68.3, 53.1, 41.8, 31.8, 28.4, 22.4, FT-IR: v/cm^{-1} = 698, 752, 1052, 1140, 1160, 1204, 1248, 132, 1640, 1729, $[\alpha]_D^{20}$ = +16 (c 1.00, $CHCl_3$), MS (ESI) m/z calculated for $[C_{28}H_{37}N_4O_8]^+$ ($[M+H]^+$): 557.2, found 557.2.

18, (*S*)-(1-(Methoxy(methyl)amino)-1-oxo-6-(2,3-bis((benzyloxy)carbonyl)guanidino)-2-((tert-butoxycarbonyl)amino)carbamate

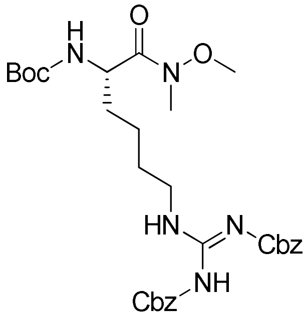

17 (4.94 g, 8.12 mmol, 1 eq), TBTU (3.64 g, 11.36 mmol, 1.4 eq) and N,O-dimethyl hydroxylamine · HCl (1.58 g, 16.24 mmol, 2 eq) were dissolved in THF (30 mL) under argon atmosphere and cooled to 0 °C. DIEA (8.48 mL, 48.72 mmol, 6 eq) was added and the reaction stirred overnight. The mixture was extracted with EA (40 mL) and washed with saturated NaCl solution, dried over $MgSO_4$ and the organic solvents were removed under reduced pressure. The crude product was purified by column chromatography (CH/EA, 1:1) to yield the product as a colorless oil (3.38 g, 5.68 mmol, 70%). ^{1}H NMR (300 MHz, $CDCl_3$): δ/ppm = 7.42 – 7.24 (m, 10H), 5.19 – 5.10 (m, 4H), 4.65 (s, 1H), 3.74 (s, 3H), 3.49 – 3.32 (m, 2H), 3.18 (s, 3H), 1.77 – 1.48 (m, 6H), 1.43 (s, 9H), ^{13}C NMR (75 MHz,

CDCl3): δ/ppm = 163.8, 156.0, 155.6, 153.9, 136.9, 134.7, 128.9, 128.8, 128.6, 128.5, 128.2, 128.0, 79.1, 68.2, 67.2, 61.7, 50.2, 41.0, 32.7, 28.7, 28.4, 22.7, FT-IR: ν/cm^{-1} = 1049, 1137, 1167, 1206, 1249, 1323, 1365, 1383, 1425, 1638, $[\alpha]_D^{20}$ = +14 (*c* 1.00, $CHCl_3$), MS (ESI) *m/z* calculated for $[C_{31}H_{42}N_5O_8]^+$ ($[M+H]^+$): 600.3, found 600.3.

19, (*S*/*R*)-1-Oxo-6-(2,3-bis((benzyloxy)carbonyl)guanidino)-2-((*tert*-butoxycarbonyl) amino)-2-benzo[d]thiazol

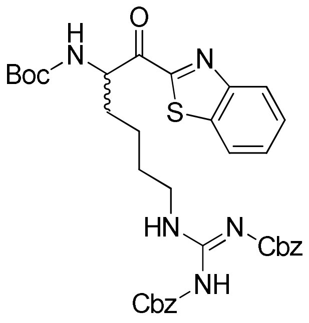

Benzothiazole **10a** (6.20 mL, 57.26 mmol, 10.2 eq) and TEMED (8.47 mL, 56.14 mmol, 10 eq) was dissolved in dry THF (90 mL) under argon atmosphere and cooled to −78 °C. *n*-BuLi (2.5 M in hexanes, 22,45 mL, 56.14 mmol, 10 eq) was added dropwise and the reaction stirred for 1.5 h at the same temperature. Afterwards, **18** (3.36 g, 5.61 mmol, 1 eq) was dissolved in dry THF (10 mL) and was added slowly to the reaction mixture and stirred for two hours at −78 °C. The reaction mixture was quenched with saturated NH_4Cl solution (15 mL). The organic phase was separated and washed three times each with saturated $NaHCO_3$ solution (20 mL) and saturated NaCl solution (20 mL). The organic phase was dried over anhydrous $NaSO_4$ and concentrated under reduced pressure. The crude product was purified by column chromatography (CH/EA, 7:1 to 3:1) to yield the product as an orange oil (2.52 g, 3.75 mmol, 67%). ^{1}H NMR (300 MHz, CDCl3): δ/ppm = 8.36 – 8.27 (m, 1H), 8.24 – 8.15 (m, 1H), 8.00 – 7.93 (m, 1H), 7.60 – 7.49 (m, 2H), 7.41 – 7.24 (m, 10H), 5.44 – 5.33 (m, 1H), 5.13 (d, *J* = 14.6 Hz, 4H), 1.80 – 1.51 (m, 6H), 1.44 (s, 9H), ^{13}C NMR (75 MHz, CDCl3): δ/ppm = 163.6, 156.0, 153.9, 153.6, 137.3, 136.8, 134.7, 128.9, 128.8, 128.6, 128.5, 128.2, 128.1, 128.0, 127.2, 126.0, 122.4, 80.0, 68.2, 67.2, 56.3, 40.9, 32.8, 28.6, 28.4, 22.9, FT-IR: ν/cm^{-1} = 754, 1050, 1139, 1163, 1215, 1256, 1319, 1382, 1638, 1695, $[\alpha]_D^{20}$ = +3 (*c* 1.00, $CHCl_3$), MS (ESI) *m/z* calculated for $[C_{35}H_{40}N_5O_7S]^+$ ($[M+H]^+$): 674.3, found 674.2.

25, (*S*)-2-((*Tert*-butoxycarbonyl)amino)-3-(4-aminophenyl)propanoic acid

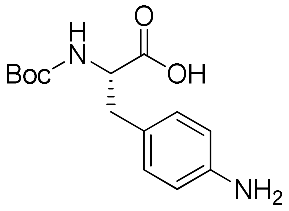

(Adapted from Kwon *et al.*)[18]

24 (1.50 g, 4.84 mmol, 1 eq) was dissolved in methanol (40 mL) and Pd/C (5%, 0.075 g) was added and the reaction stirred for 3 h under H_2-atmosphere (3 bar). The mixture was then filtered over Celite® and the solvent was evaporated under reduced pressure. The crude product was used without further purification for the next step.

MS (ESI) *m/z* calculated for $[C_{14}H_{21}N_2O_4]^+$ ($[M+H]^+$): 281.1, found 303.1 ($[M+Na]^+$).

26, (*S*)-3-(4-(2,3-Bis((benzyloxy)carbonyl)guanidino)phenyl)-2-((*tert*-butoxycarbonyl) amino)propanoic acid

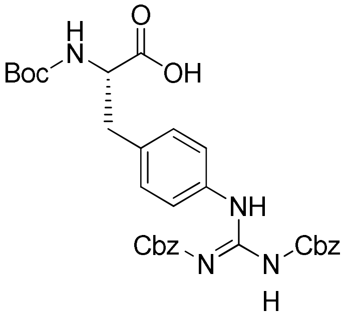

(Adapted from Kwon *et al.*)[18]

25 (1.35 g, 4.84 mmol, 1 eq), *N,N´*-bis-(carbobenzoxy)-1*H*-pyrazole-1-carboxamidine (2.19 g, 5.80 mmol, 1.2 eq) were dissolved in DMF (30 mL) and Et_3N (1.00 mL, 7.25 mmol, 1.5 eq) was added under argon atmosphere. The reaction stirred for 6 h and got quenched by addition of NH_4Cl (10 mL). The mixture was extracted with EA (30 mL), dried over $MgSO_4$ and the organic solvents were removed under reduced pressure. The crude product was purified by column chromatography (CH/EA, 4:1 to 2:1 + 0.1% TFA) to yield the product as a colorless solid (2.86 g, 4.84 mmol, quant.). ^{1}H NMR (300 MHz, CDCl3): δ/ppm = 7.46 – 7.27 (m, 10H), 7.14 (d, *J* = 7.9 Hz, 4H), 5.28 – 5.10 (m, 4H), 4.53 (s, 1H), 3.13 – 2.98 (m, 2H), 1.41 (s, 9H), ^{13}C NMR (75 MHz, CDCl3): δ/ppm = 175.0, 161.9, 156.1, 154.5, 149.3, 135.2, 135.1, 130.7, 129.4, 129.3, 129.1, 129.0, 128.7, 123.9, 69.0, 56.0, 38.8, 28.8, FT-IR: ν/cm^{-1} = 752, 911, 1003, 1172, 1378, 1456, 1496, 1532, 1726, 1792, mp: 117 – 122 °C, $[\alpha]_D^{20}$ = +42 (*c* 1.00, $CHCl_3$), MS (ESI) *m/z* calculated for $[C_{31}H_{35}N_4O_8]^+$ ($[M+H]^+$): 591.2, found 591.0.

27, (*S*) -(1-(Methoxy(methyl)amino)-1-oxo -3-(4-(2,3-bis((benzyloxy)carbonyl) guanidino)phenyl)-2-((*tert*-butoxycarbonyl)amino)carbamate

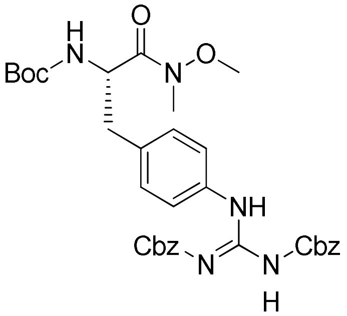

(Adapted from Kwon *et al.*)[18]

26 (3.00 g, 5.08 mmol, 1 eq), TBTU (2.28 g, 7.12 mmol, 1.4 eq) and *N,O*-dimethyl hydroxylamine · HCl (0.99 g, 10.16 mmol, 2 eq) were dissolved in THF (15 mL) and DIEA (1.16 mL, 6.66 mmol, 6 eq) was added under argon atmosphere. The reaction stirred for 12 h and the mixture was extracted with EA (45 mL), washed with saturated NaCl solution (20 mL), dried over $MgSO_4$ and the was removed under reduced pressure. The crude product was purified by column chromatography (CH/EA, 2:1) to yield the product as a colorless oil (1.64 g, 2.85 mmol, 51%.). ^{1}H NMR (300 MHz, CDCl3): δ/ppm = 11.91 (s, 1H), 10.25 (s, 1H), 7.54 – 7.45 (m, 2H), 7.44 – 7.28 (m, 10H), 7.19 – 7.10 (m, 2H), 5.24 (s, 2H), 5.14 (s, 2H), 4.91 (s, 1H), 3.67 (s, 3H), 3.15 (s, 3H), 3.02 (dd, *J* = 13.7, 6.0 Hz, 1H), 2.85 (dd, *J* = 13.8, 7.0 Hz, 1H), 1.39 (s, 9H), ^{13}C NMR (75 MHz, CDCl3): δ/ppm = 176.8, 172.9, 169.5, 160.3, 155.3, 154.1, 153.7, 130.1, 128.9, 128.6, 128.2, 128.1, 122.6, 68.7, 67.59, 61.7, 51.6, 38.3, 32.2, 28.5, FT-IR: ν/cm^{-1} = 754, 1055, 1220, 1265, 1294, 1367, 1383, 1422, 1608, 1639, $[\alpha]_D^{20}$ = +21 (*c* 1.00, $CHCl_3$), MS (ESI) *m/z* calculated for $[C_{33}H_{40}N_5O_8]^+$ ($[M+H]^+$): 634.3, found 634.0.

28, (*S*/*R*) -1-Oxo -3-(4-(2,3-bis((benzyloxy)carbonyl)guanidino)phenyl)-2-((*tert*-butoxycarbonyl)amino)2-benzo[*d*]thiazol

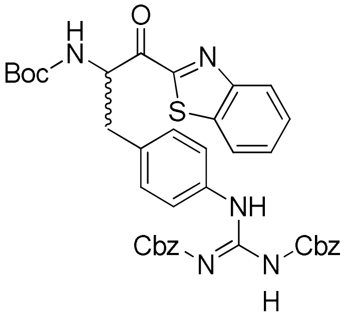

(Adapted from Kwon *et al.*)[18]

Benzothiazole **10a** (2.86 mL g, 26.42 mmol, 10.2 eq) and TEMED (3.91 mL, 25.90 mmol, 10 eq) were dissolved in dry THF (35 mL) under argon atmosphere and was cooled to −78 °C. *n*-BuLi (2.5 M in hexanes, 10.36 mL, .25.90 mmol, 10 eq) was added dropwise and the reaction stirred for 1.5 h at the same temperature. Afterwards, **27** (1.64 g, 2.59 mmol, 1 eq) was dissolved in dry THF (15 mL) and was added slowly to the reaction mixture and stirred for two hours at −78 °C. The reaction mixture was quenched with saturated NH_4Cl solution (25 mL). The organic phase was separated and washed three times each with saturated $NaHCO_3$ solution (25 mL) and saturated NaCl solution (25 mL). The organic phase was dried over anhydrous $NaSO_4$ and concentrated under reduced pressure. The crude product was purified by column chromatography (CH/EA, 9:1 to 2:1) to yield the product as a brown solid (1.18 g, 1.66 mmol, 64%). ^{1}H NMR (300 MHz, CDCl3): δ/ppm = 8.24 (dd, *J* = 7.4, 1.7 Hz, 1H), 8.02 – 7.96 (m, 1H), 7.58 (pd, *J* = 7.2, 1.5 Hz, 2H), 7.46 (d, *J* = 8.4 Hz, 2H), 7.41 – 7.28 (m, 10H), 7.09 (d, *J* = 8.0 Hz, 2H), 5.86 (d, *J* = 7.2 Hz, 1H), 5.24 (s, 2H), 5.13 (s, 2H), 3.43 (dd, *J* = 14.1, 5.1 Hz, 1H), 3.25 – 3.15 (m, 1H), 1.41 (s, 9H), ^{13}C NMR (75 MHz, CDCl3): δ/ppm = 183.7, 164.0, 155.2, 154.6, 154.0, 153.7, 153.5, 137.4, 127.3, 129.1, 128.9, 128.6, 128.2, 126.0, 122.6, 68.8, 67.8, 57.7, 38.5, 28.4, FT-IR: ν/cm^{-1} = 733, 755, 1055, 1218, 1294, 1367, 1422, 1607, 1632, 1694, mp: 175 – 178 °C, $[\alpha]_D^{20}$ = +6 (*c* 1.00, $CHCl_3$), MS (ESI) *m/z* calculated for $[C_{38}H_{38}N_5O_7S]^+$ ($[M+H]^+$): 708.2, found 708.3.

41, (*S*)-2-((*Tert*-butoxycarbonyl)amino)-3-(3-aminophenyl)propanoic acid

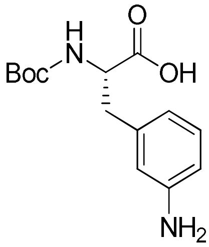

(Adapted from Kwon *et al.*)[18]

40 (1.00 g, 3.22 mmol, 1 eq) was dissolved in methanol (26 mL) and Pd/C (5%, 0.05 g) was added and the reaction stirred for 3 h under H_2-atmosphere (3 bar). The mixture was then filtered over Celite® and the solvent was evaporated under reduced pressure. The crude product was used without further purification for the next step.

MS (ESI) *m/z* calculated for $[C_{14}H_{21}N_2O_4]^+$ ($[M+H]^+$): 281.1, found 181.0 ($[M+H–Boc]^+$).

42, (*S*)-3-(3-(2,3-Bis((benzyloxy)carbonyl)guanidino)phenyl)-2-((tert-butoxycarbonyl) amino)propanoic acid

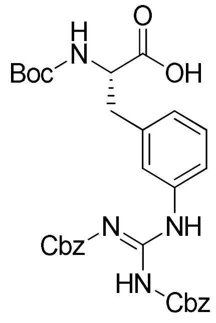

(Adapted from Kwon *et al.*)[18]

41 (0.90 g, 3.22 mmol, 1 eq), *N,N´*-bis-(carbobenzoxy)-1*H*-pyrazole-1-carboxamidine (1.46 g, 3.86 mmol, 1.2 eq) were dissolved in DMF (30 mL) and Et_3N (0.67 mL, 4.83 mmol, 1.5 eq) was added under argon atmosphere. The reaction stirred for 6 h and got quenched by addition of NH_4Cl (10 mL). The mixture was extracted with EA (30 mL), dried over $MgSO_4$ and the organic solvents were removed under reduced pressure. The crude product was purified by column chromatography (CH/EA, 3:1 to 1:1 + 0.1% TFA) to yield the product as a colorless oil (1.70 g, 2.89 mmol, 90%). ^{1}H NMR (300 MHz, CDCl3): δ/ppm = 7.49 – 7.08 (m, 14H), 5.35 – 5.26 (m, 2H), 5.22 – 5.10 (m, 2H), 4.59 – 4.37 (m, 1H), 3.10 (s, 2H), 1.50 – 1.29 (m, 9H), ^{13}C NMR (75 MHz, CDCl3): δ/ppm = 173.9, 173.0, 160.6, 160.1, 153.8, 146.8, 130.3, 129.5, 129.0, 128.7, 128.6, 117.3, 113.5, 80.4, 70.3, 69.9, 54.7, 38.6, 28.4, FT-IR: ν/cm^{-1} = 731, 900, 1051, 1164, 1209, 1318, 1504, 1608, 1701, 1748, $[\alpha]_D^{20}$ = +20 (*c* 1.00, $CHCl_3$), MS (ESI) *m/z* calculated for $[C_{31}H_{35}N_4O_8]^+$ ($[M+H]^+$): 591.2, found 591.1.

43, (*S*) -(1-(Methoxy(methyl)amino)-1-oxo -3-(3-(2,3-bis((benzyloxy)carbonyl) guanidino)phenyl)-2-((*tert*-butoxycarbonyl)amino)carbamate

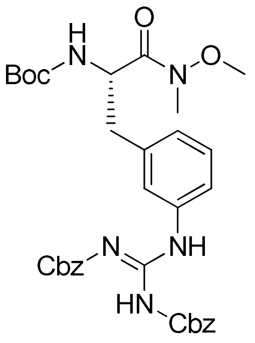

42 (2.26 g, 3.83 mmol, 1 eq), TBTU (1.47 g, 4.60 mmol, 1.2 eq) and *N,O*-dimethyl hydroxylamine · HCl (2.24 g, 23.00 mmol, 6 eq) were dissolved in THF/DMF (4:1, 20 mL) and DIEA (2.67 mL, 15.32 mmol, 4 eq) was added under argon atmosphere. The reaction was stirred for 12 h and the mixture was extracted with EA (40 mL), washed with saturated NaCl solution (20 mL), dried over $MgSO_4$ and the was removed under reduced pressure. The crude product was purified by column chromatography (CH/EA, 2:1) to yield the product as a colorless oil (1.28 g, 2.02 mmol, 53%.). ^{1}H NMR (300 MHz, CDCl3): δ/ppm = 7.55 – 7.20 (m, 14H), 6.97 (d, *J* = 7.6 Hz, 1H), 5.27 – 5.11 (m, 4H), 4.91 (s, 1H), 3.63 (s, 3H), 3.13 (s, 3H), 3.05 (dd, *J* = 13.6, 5.7 Hz, 1H), 2.85 (dd, *J* = 13.7, 7.6 Hz, 1H), 1.39 (s, 9H), ^{13}C NMR (75 MHz, CDCl3): δ/ppm = 172.2, 163.8, 155.2, 153.6, 137.8, 136.4, 134.6, 129.0, 128.8, 128.6, 128.5, 128.2, 128.0, 126.5, 123.4, 121.2, 79.7, 68.6, 67.5, 61.6, 51.6, 38.8, 32.1, 28.4, FT-IR: ν/cm^{-1} = 695, 731, 905, 1049 1116, 1161, 1231, 1422, 1600, 1729, $[\alpha]_D^{20}$ = +26 (*c* 1.00, $CHCl_3$), MS (ESI) *m/z* calculated for $[C_{33}H_{40}N_5O_8]^+$ ($[M+H]^+$): 634.3, found 634.2.

44, (*S*/*R*) -1-Oxo -3-(3-(2,3-bis((benzyloxy)carbonyl)guanidino)phenyl)-2-((*tert*-butoxycarbonyl)amino)2-benzo[*d*]thiazol

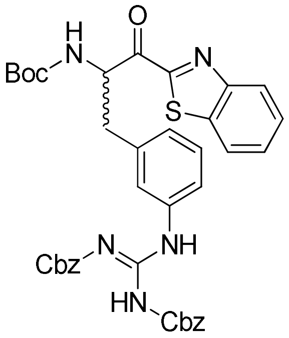

(Adapted from Kwon *et al.*)[18]

Benzothiazole **10a** (2.22 mL g, 20.60 mmol, 10.2 eq) and TEMED (3.04 mL, 20.20 mmol, 10 eq) were dissolved in dry THF (30 mL) under argonatmosphere and was cooled to −78 °C. *n*-BuLi (2.5 M in hexanes, 8.08 mL, .20.20 mmol, 10 eq) was added dropwise and the reaction stirred for 1.5 h at the same temperature. Afterwards, **43** (1.28 g, 2.02 mmol, 1 eq) was dissolved in dry THF (10 mL) and was added slowly to the reaction mixture and stirred for two hours at −78 °C. The reaction mixture was quenched with saturated NH_4Cl solution (20 mL). The organic phase was separated and washed three times each with saturated $NaHCO_3$ solution (20 mL) and saturated NaCl solution (20 mL). The organic phase was dried over anhydrous $NaSO_4$ and concentrated under reduced pressure. The crude product was purified by column chromatography (CH/EA, 7:1 to 3:1) to yield the product as a brown solid (1.20 g, 1.69 mmol, 84%). ^{1}H NMR (300 MHz, CDCl3): δ/ppm = 8.23 (d, *J* = 7.7 Hz, 1H), 8.02 – 7.93 (m, 1H), 7.63 – 7.51 (m, 3H), 7.44 – 7.17 (m, 12H), 6.95 (d, *J* = 7.6 Hz, 1H), 5.92 – 5.79 (m, 1H), 5.38 – 5.06 (m, 5H), 3.50 – 3.05 (m, 2H), 1.40 (s, 9H) ppm, ^{13}C NMR (75 MHz, CDCl3): δ/ppm = 192.9, 163.8, 155.2, 153.9, 153.5, 137.3, 137.2, 136.1, 129.4, 129.0, 128.8, 128.7, 128.5, 128.1, 127.3, 126.9, 126.0, 123.6, 122.5, 121.6, 86.3, 80.1, 68.8, 67.8, 57.5, 52.8, 38.6, 28.3 ppm, FT-IR: ν/cm^{-1} = 726, 1046, 1167, 1240, 1366, 1422, 1484, 1639, 1687, 2974, mp: 164 – 166 °C, $[\alpha]_D^{20}$ = +5 (*c* 1.00, $CHCl_3$), MS (ESI) *m/z* calculated for $[C_{38}H_{38}N_5O_7S]^+$ ($[M+H]^+$): 708.2, found 708.1.

w

34, (*S*)-2-((*Tert*-butoxycarbonyl)amino)-3-(4-aminocyclohexyl)propanoic acid

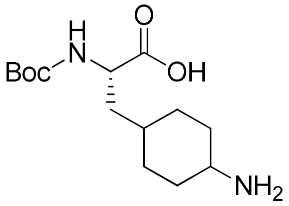

(Adapted from Kwon *et al.*)[18]

24 (1.50 g, 4.84 mmol, 1 eq) was dissolved in AcOH/MeOH (1:1, 30 mL) and PtO_2 (0.06 g, 0.48 mmol, 0.1 eq) was added. The reaction stirred for 24 h under H_2 atmosphere (3 bar) at rt. The mixture was filtered over Celite® and the solution was removed under reduced pressure. The crude product was used without further purification for the next step.

MS (ESI) *m/z* calculated for $[C_{14}H_{26}N_2O_4]^+$ ($[M+H]^+$): 286.2, found 326.0 ($[M+K]^+$).

35, (*S*)-3-(4-(2,3-Bis((benzyloxy)carbonyl)guanidino)cyclohexyl)-2-((tert-butoxycarbonyl)amino)propanoic acid

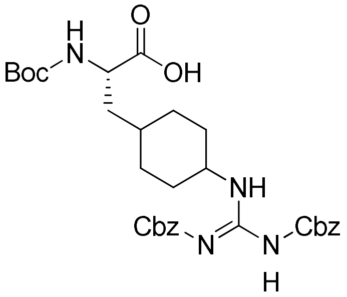

(Adapted from Kwon *et al.*)[18]

34 (1.19 g, 4.16 mmol, 1 eq), *N,N′* bis-(carbobenzoxy)-1-*H*-pyrazol-1-carboxamidin (2.01 g, 5.32 mmol, 1.1 eq) were dissolved in DMF (30 mL) and Et_3N (0.86 mL, 6.25 mmol, 1.5 eq) was added under argon atmosphere. The reaction stirred for 6 h and got quenched by addition of NH_4Cl (10 mL). The mixture was extracted with EA (30 mL), dried over $MgSO_4$ and the organic solvents were removed under reduced pressure. The crude product was purified by column chromatography (DCM/MeOH, 100:1 to 11:1) to yield the product as a colorless solid (2.48 g, 4.16 mmol, quant.). ^{1}H NMR (300 MHz, CDCl3): δ/ppm = 7.41 – 7.33 (m, 10H), 5.21 (d, *J* = 8.3 Hz, 2H), 5.02 (s, 2H), 4.10 – 3.95 (m, 1H), 3.88 (d, *J* = 7.5 Hz, 1H), 1.90 – 1.41 (m, 6H), 1.34 (d, *J* = 17.7 Hz, 9H), 1.03 (ddd, *J* = 50.6, 24.1, 12.0 Hz, 5H) ppm, ^{13}C NMR (75 MHz, CDCl3): δ/ppm = 163.1, 155.5, 155.1, 154.3, 152.8, 135.3, 128.5, 128.4, 128.2, 122.2, 77, 67.8, 66.4, 56.8, 51.9, 38.4, 31.6, 30.2, 30.1, 29.4, 28.3, 28.2 ppm, FT-IR: ν/cm^{-1} = 696, 733, 1053, 1124, 1162, 1206, 1295, 1367, 1420, 1635, mp: 98 – 112 °C, $[\alpha]_D^{20}$ = +18 (*c* 1.00, $CHCl_3$), MS (ESI) *m/z* calculated for $[C_{31}H_{41}N_4O_8]^+$ ($[M+H]^+$): 597.3, found 597.3.

36, (*S*) -(1-(Methoxy(methyl)amino)-1-oxo -3-(4-(2,3 bis((benzyloxy)carbonyl) guanidino)cyclohexyl)-2-((tert-butoxycarbonyl)amino)carbamate

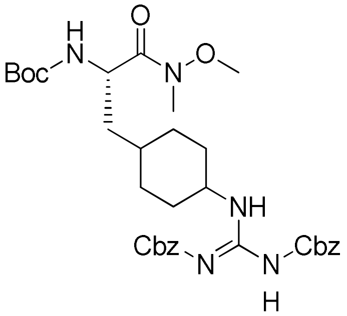

(Adapted from Kwon *et al.*)[18]

35 (0.66 g, 1.11 mmol, 1 eq), TBTU (0.49 g, 1.55 mmol, 1.4 eq) and *N,O*-dimethyl hydroxylamine · HCl (0.21 g, 2.22 mmol, 2 eq) were dissolved in THF (7 mL) and DIEA (1.16 mL, 6.66 mmol, 6 eq) was added under argon atmosphere. The reaction stirred for 12 h and and the mixture was extracted with EA (20 mL), washed with saturated NaCl solution (10 mL), dried over $MgSO_4$ and the was removed under reduced pressure. The crude product was purified by column chromatography (CH/EA, 2:1) to yield the product as a colorless solid (1.64 g, 2.85 mmol, 51%.). ^{1}H NMR (300 MHz, CDCl3): δ/ppm = 7.49 (d, *J* = 8.5 Hz, 1H), 7.43 – 7.28 (m, 10H), 7.13 (d, *J* = 8.5 Hz, 1H), 5.24 – 5.05 (m, 4H), 4.71 (d, *J* = 9.2 Hz, 1H), 3.77 (d, *J* = 5.8 Hz, 2H), 3.67 (s, 1H), 3.24 – 3.13 (m, 3H), 3.02 (dd, *J* = 13.7, 6.0 Hz, 1H), 2.87 (d, *J* = 7.1 Hz, 1H), 2.07 – 1.51 (m, 7H), 1.41 (d, *J* = 14.9 Hz, 9H), 1.28 – 1.03 (m, 4H) ppm, ^{13}C NMR (75 MHz, CDCl3): δ/ppm = 156.4, 155.1, 154.0, 149.6, 136.9, 128.8, 128.5, 128.2, 79.8, 67.4, 61.7, 52.7, 32.7, 32.3, 32.3, 29.5, 28.5 ppm, FT-IR: ν/cm^{-1} = 1053,1170, 1227, 1295, 1337, 1366, 1383, 1428, 1639, 1711, mp: 70 – 77 °C, $[\alpha]_D^{20}$ = –20 (*c* 1.00, $CHCl_3$), MS (ESI) *m/z* calculated for $[C_{33}H_{46}N_5O_8]^+$ ($[M+H]^+$): 640.3, found 640.3.

37, (*S*/*R*) -1-Oxo -3-(4-(2,3-bis((benzyloxy)carbonyl)guanidino)cyclohexyl)-2-((tert-butoxycarbonyl)amino)2-benzo[*d*]thiazol

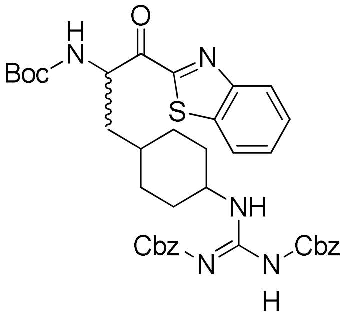

(Adapted from Kwon *et al.*)[18]

Benzothiazole **10a** (0.88 mL g, 8,02 mmol, 10.2 eq) and TEMED (1.19 mL, 7.86 mmol, 10 eq) were dissolved in dry THF (13 mL) under argonatmosphere and was cooled to −78 °C. *n*-BuLi (2.5 M in hexanes, 3.15 mL, .7.86 mmol, 10 eq) was added dropwise and the reaction stirred for 40 min at the same temperature. Afterwards, **36** (0.50 g, 0.78 mmol, 1 eq) was dissolved in dry THF (5 mL) and was added slowly to the reaction mixture and stirred for two hours at −78 °C. The reaction mixture was quenched with saturated NH_4Cl solution (20 mL). The organic phase was separated and washed three times each with saturated $NaHCO_3$ solution (20 mL) and saturated NaCl solution (20 mL). The organic phase was dried over anhydrous $NaSO_4$ and concentrated under reduced pressure. The crude product was purified by column chromatography (CH/EA, 9:1 to 4:1) to yield the product as a yellow solid (0.42 g, 0.59 mmol, 76%). ^{1}H NMR (300 MHz, CDCl3): δ/ppm = 8.26 – 8.18 (m, 1H), 8.02 – 7.95 (m, 1H), 7.57 (dddd, *J* = 8.8, 6.7, 4.0, 2.1 Hz, 2H), 7.42 – 7.27 (m, 10H), 5.26 (d, *J* = 17.0 Hz, 2H), 5.20 – 5.11 (m, 3H), 3.43 (dd, *J* = 14.0, 5.1 Hz, 1H), 3.21 (dd, *J* = 14.1, 6.9 Hz, 1H), 2.30 – 1.48 (m, 8H), 1.43 (d, *J* = 9.0 Hz, 9H), 1.32 – 1.12 (m, 3H), ^{13}C NMR (75 MHz, CDCl3): δ/ppm = 193.0, 164.0, 160.3, 156.3, 154.9, 149.7, 136.7, 136.6, 128.9, 128.6, 128.1, 127.3, 126.0, 122.6, 80.1, 68.6, 67.6, 50.4, 33.7, 30.5, 28.4, 28.3, FT-IR: ν/cm^{-1} = 733, 755, 1055, 1218, 1294, 1367, 1422, 1607, 1632, 1694, mp: 82 – 93 °C, $[\alpha]_D^{20}$ = +6 (*c* 1.00, $CHCl_3$), MS (ESI) *m/z* calculated for $[C_{38}H_{43}N_5O_7S]^+$ ($[M+H]^+$): 714.2, found 714.0.

General procedure for the amide bond formation between the H_2N-P1-warhead moiety and the peptide residue

The respective Boc-NH-P1-warhead moieties **12a**–**f**, **13**, **19, 27, 36, 43** were deprotected by using a deprotection mixture of 4 M HCl in dioxane (4 ml) or 16% TFA in DCM (4 mL) and stirred for 30 min at rt. The solvent was removed under reduced pressure to yield the deprotected amines as hydrochloride or trifluoroacetate salts.

The carboxylic acid of different peptide backbones **3**, **21**–**23** (1.2 eq) was dissolved in DCM and cooled to 0 °C with an ice-water bath. DIEA (6 eq) and HATU (1.2 eq) were added and stirring was continued for 30 min at 0 °C. Then, the respective deprotected amine (1 eq) as a solution in DMF were added, stirred for 30 min at 0 °C and 16 h at room temperature. DCM and water were added, and the aqueous phase was extracted three times with DCM. The combined organic phases were dried over Na_2SO_4, and the solvent was removed under reduced pressure. The crude product was purified with reversed-phase flash chromatography performed with the Biotage Isolera™ and used without further analysis for the next step.

Synthesis of uPA targeting compounds

14a, (2*S*,3*S*)-2-(2-Acetamidoacetamido)-*N*-((*S*)-1-(((*S*)-1-(benzo[*d*]thiazol-2-yl)-5-guanidino-1-oxopentan-2-yl)amino)-1-oxopropan-2-yl)-3-hydroxybutanamide

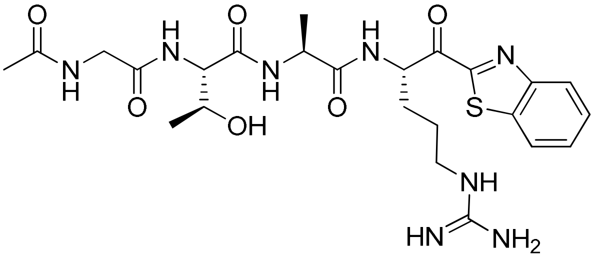

After the coupling of the respective peptide backbone **3** with the P1-warhead moiety **12a**, the deprotection of the Pbf- and O^{t}Bu-protecting group was performed in a TFA/DCM solution (50%, 2 mL) and stirred for 2 h at room temperature. The solution was evaporated under reduced pressure and purified by preparative HPLC (12.8 mg, 0.017 mmol, 10%). ^{1}H NMR (400 MHz, DMSO-d_6): δ/ppm = 8.57 – 8.39 (m, 1H), 8.31 – 8.24 (m, 2H), 8.23 – 8.16 (m, 1H), 8.08 – 7.96 (m, 1H), 7.83 – 7.72 (m, 1H), 7.71 – 7.63 (m, 2H), 7.60 – 7.54 (m, 1H), 5.54 – 5.41 (m, 1H), 5.00 (d, *J* = 35.3 Hz, 1H), 4.36 (ddd, *J* = 10.0, 7.2, 2.7 Hz, 1H), 4.17 (tdd, *J* = 12.1, 8.0, 4.2 Hz, 1H), 3.97 (ddt, *J* = 16.9, 11.2, 5.2 Hz, 1H), 3.83 – 3.69 (m, 2H), 3.14 (p, *J* = 6.6 Hz, 2H), 1.97 (td, *J* = 13.7, 11.4, 6.7 Hz, 2H), 1.90 – 1.82 (m, 2H), 1.79 – 1.69 (m, 0H), 1.65 – 1.54 (m, 2H), 1.27 – 1.17 (m, 3H), 1.06 – 0.98 (m, 3H). ^{13}C NMR (400 MHz, DMSO-d_6): δ/ppm = 193.1, 172.6, 169.9, 169.7, 169.5, 164.5, 156.8, 153.0, 136.5, 128.3, 127.6, 125.3, 123.3, 66.7, 58.2, 54.3, 47.9, 42.2, 42.2, 27.8, 25.2, 22.4, 19.7, 18, mp: 68 – 75 °C, $[\alpha]_D^{20}$ = +34 (*c* 1.00, DMSO), FT-IR: ν/cm^{-1} = 660, 668, 756, 1136, 1215, 1673, 1684, 2922, 2964, 3327, MS (ESI) *m/z* calculated for $[C_{24}H_{35}N_8O_6S]^+$ ($[M+H]^+$): 563.2, found 563.2. Purity: 97%.

14f, (2*S*,3*S*)-2-(2-Acetamidoacetamido)-*N*-((*S*)-1-(((*S*)-1-(benzo[b]thiophen-2-yl)-5-guanidino-1-oxopentan-2-yl)amino)-1-oxopropan-2-yl)-3-hydroxybutanamide

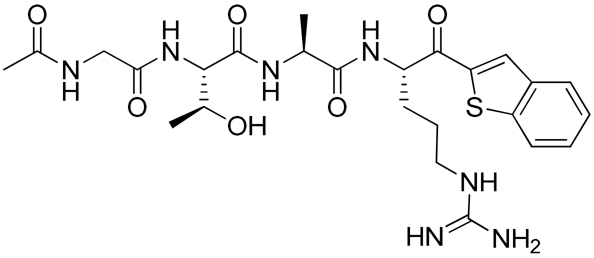

After the coupling of the respective peptide backbone **3** with the P1-warhead moiety **12f**, the deprotection of the Pbf- and O^{t}Bu-protecting group was performed in a TFA/DCM solution (50%, 2 mL) and stirred for 2 h at room temperature. The solution was evaporated under reduced pressure and purified by preparative HPLC (16.8 mg, 0.03 mmol, 8%). ^{1}H NMR (600 MHz, DMSO-d_6): δ/ppm = 8.53 (d, *J* = 7.0 Hz, 1H), 8.46 (d, *J* = 4.7 Hz, 1H), 8.43 – 8.35 (m, 1H), 8.33 – 8.25 (m, 1H), 8.19 (dt, *J* = 8.6, 6.1 Hz, 1H), 8.11 – 7.97 (m, 3H), 7.81 (dd, *J* = 17.4, 8.0 Hz, 1H), 7.58 – 7.48 (m, 2H), 5.27 (dddd, *J* = 32.2, 9.1, 7.5, 4.8 Hz, 1H), 5.00 (dd, *J* = 29.7, 5.0 Hz, 1H), 4.35 – 4.26 (m, 1H), 4.22 – 4.11 (m, 1H), 4.04 – 3.91 (m, 1H), 3.81 – 3.69 (m, 2H), 3.17 – 3.08 (m, 2H), 1.86 (dd, *J* = 7.3, 6.1 Hz, 4H), 1.71 – 1.50 (m, 3H), 1.24 – 1.10 (m, 4H), 1.02 (d, *J* = 6.3 Hz, 2H) ppm, ^{13}C NMR (151 MHz, DMSO-d_6): δ/ppm = 193.3, 172.4, 169.9, 169.5, 156.6, 141.6, 141.1, 141.0, 139.1, 131.2, 130.9, 128.0, 126.5, 125.4, 123.1, 66.7, 58.7, 54.1, 48.4, 42.2, 40.3, 28.3, 25.0, 22.5, 19.7, 18.2 ppm, FT-IR: ν/cm^{-1} = 716, 757, 929, 1028, 1129, 1202, 1368, 1512, 1644, 3291, mp: 74 – 80 °C, $[\alpha]_D^{20}$ = +23 (*c* 1.00, DMSO), MS (ESI) *m/z* calculated for $[C_{25}H_{36}N_7O_6S]^+$ ($[M+H]^+$): 562.2, found 562.3. Purity: 99%.

14e, (2*S*,3*S*)-2-(2-Acetamidoacetamido)-*N*-((*S*)-1-(((*S*)-5-guanidino-1-(6-methoxybenzo [d]thiazol-2-yl)-1-oxopentan-2-yl)amino)-1-oxopropan-2-yl)-3-hydroxybutanamide

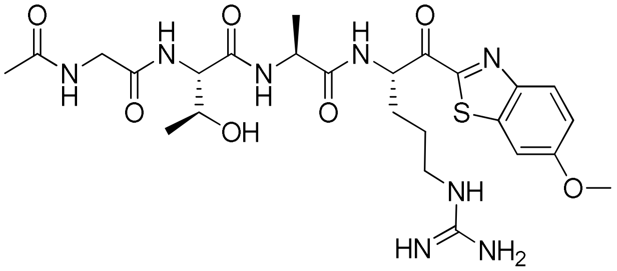

After the coupling of the respective peptide backbone **3** with the P1-warhead moiety **12e**, the deprotection of the Pbf- and O^{t}Bu-protecting group was performed in a TFA/DCM solution (50%, 2 mL) and stirred for 2 h at room temperature. The solution was evaporated under reduced pressure and purified by preparative HPLC (11.8 mg, 0.02 mmol, 5%). ^{1}H NMR (600 MHz, DMSO-*d*$_6$): *δ*/ppm = 8.39–8.n18 (m, 2H), 8.53 – 8.51 (m, 1H), 8.42 – 8.36 (m, 1H), 8.25 – 8.18 (m, 1H), 8.16 – 8.12 (m, 1H), 8.09 – 7.97 (m, 1H), 7.89 (d, *J* = 8.1 Hz, 1H), 7.84 – 7.75 (m, 2H), 7.56 – 7.51 (m, 1H), 7.29 – 7.26 (m, 1H), 5.52 – 5.40 (m, 1H), 5.07 – 4.94 (m, 1H), 4.40 – 4.31 (m, 1H), 4.21 – 4.13 (m, 1H), 4.03 – 3.91 (m, 1H), 3.89 (s, 3H), 3.81 – 3.68 (m, 2H), 3.16 – 3.09 (m, 2H), 1.98 – 1.90 (m, 1H), 1.87 – 1.82 (m, 3H), 1.78 – 1.53 (m, 3H), 1.25 – 1.18 (m, 3H), 1.05 – 0.99 (m, 3H) ppm, ^{13}C NMR (151 MHz, DMSO-*d*$_6$): *δ*/ppm = 192.8, 172.5, 172.3, 169.8, 169.6, 169.4, 169.3, 161.7, 159.7, 158.3, 156.6, 147.5, 138.8, 126.2, 118.2, 104.7, 66.7, 58.6, 58.2, 56.0, 54.1, 53.8, 48.2, 47.7, 42.1, 40.3, 27.9, 25.1, 22.5, 19.7, 18.4, 18.0 ppm, FT-IR: *ν*/cm^{-1} = 827, 886, 929, 1024, 1206, 1366, 1488, 1532, 1636, 3289, mp: 92 – 96 °C, $[\alpha]_D^{20}$ = +24 (*c* 1.00, DMSO), MS (ESI) *m/z* calculated for $[C_{25}H_{37}N_8O_7S]^+$ ($[M+H]^+$): 593.2, found 593.2. Purity: 99%.

14b, (2*S*,3*S*)-2-(2-Acetamidoacetamido)-*N*-((*S*)-1-(((*S*)-5-guanidino-1-(6-fluorobenzo [d]thiazol-2-yl)-1-oxopentan-2-yl)amino)-1-oxopropan-2-yl)-3-hydroxybutanamide

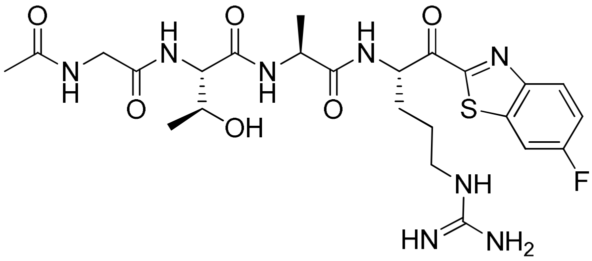

After the coupling of the respective peptide backbone **3** with the P1-warhead moiety **12b**, the deprotection of the Pbf- and O^{t}Bu-protecting group was performed in a TFA/DCM solution (50%, 2 mL) and stirred for 2 h at room temperature. The solution was evaporated under reduced pressure and purified by preparative HPLC (11.6 mg, 0.02 mmol, 4%). ^{1}H NMR (600 MHz, DMSO-*d*$_6$): *δ*/ppm = 8.57 (d, *J* = 7.2 Hz, 1H), 8.41 (d, *J* = 6.3 Hz, 1H), 8.33 – 8.23 (m, 3H), 8.22 – 8.13 (m, 2H), 7.98 (d, *J* = 6.8 Hz, 1H), 7.60 – 7.55 (m, 1H), 7.39 – 7.33 (m, 1H), 5.40 – 5.32 (m, 1H), 4.50 – 4.22 (m, 3H), 4.21 – 4.01 (m, 1H), 3.74 – 3.63 (m, 2H), 3.15 – 3.09 (m, 2H), 1.97 – 1.91 (m, 1H), 1.82 – 1.50 (m, 3H), 1.23 – 1.11 (m, 6H) ppm, ^{13}C NMR (151 MHz, DMSO-*d*$_6$): *δ*/ppm = 192.6, 172.4, 170.2, 170.1, 169.9, 169.8, 169.7, 169.4, 169.3, 168.9, 168.7, 164.8, 162.2, 160.5, 156.5, 149.9, 137.9, 127.1, 116.8, 109.4, 72.6, 57.2, 54.5, 48.3, 42.0, 40.4, 27.2, 25.2, 22.5, 18.1, 17.9, 17.6, 17.5, 17.4 ppm, FT-IR: *ν*/cm^{-1} = 890, 931, 1022, 1184, 1249, 1370, 1482, 1532, 1654, 3295, mp: 89 – 95 °C, $[\alpha]_D^{20}$ = +37 (*c* 1.00, DMSO), MS (ESI) *m/z* calculated for $[C_{24}H_{34}FN_8O_6S]^+$ ($[M+H]^+$): 581.2, found 581.1. Purity: 98%.

14c, (2*S*,3*S*)-2-(2-Acetamidoacetamido)-*N*-((*S*)-1-(((*S*)-5-guanidino-1-(6-chlorobenzo [d]thiazol-2-yl)-1-oxopentan-2-yl)amino)-1-oxopropan-2-yl)-3-hydroxybutanamide

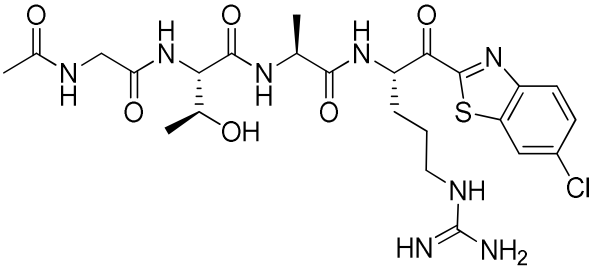

After the coupling of the respective peptide backbone **3** with the P1-warhead moiety **12c**, the deprotection of the Pbf- and O^{t}Bu-protecting group was performed in a TFA/DCM solution (50%, 2 mL) and stirred for 2 h at room temperature. The solution was evaporated under reduced pressure and purified by preparative HPLC (17.9 mg, 0.03 mmol, 8%). ^{1}H NMR (600 MHz, DMSO-d_6): δ/ppm = 8.58 (d, *J* = 6.4 Hz, 1H), 8.49 – 8.42 (m, 1H), 8.32 – 8.17 (m, 2H), 8.08 (d, *J* = 7.5 Hz, 1H), 8.02 – 7.96 (m, 1H), 7.91 – 7.70 (m, 2H), 7.54 (q, *J* = 5.9 Hz, 1H), 5.48 – 5.37 (m, 1H), 5.07 – 4.93 (m, 1H), 4.39 – 4.30 (m, 1H), 4.21 – 4.11 (m, 1H), 4.03 – 3.89 (m, 1H), 3.83 – 3.67 (m, 1H), 3.16 – 3.09 (m, 2H), 1.99 – 1.91 (m, 1H), 1.88 – 1.83 (m, 3H), 1.73 – 1.51 (m, 3H), 1.25 – 1.17 (m, 3H), 1.01 (m, 3H). ppm, ^{13}C NMR (151 MHz, DMSO-d_6): δ/ppm = 193.0, 172.5, 170.0, 169.8, 169.7, 169.6, 169.5, 169.3, 165.4, 165.3, 158.5, 158.3, 158.1, 157.9, 156.6, 151.7, 137.3, 132.9, 128.3, 126.5, 122.9, 118.2, 116.2, 66.7, 66.5, 66.3, 58.5, 58.2, 58.0, 54.3, 54.0, 48.3, 48.1, 47.8, 42.3, 42.1, 40.3, 39.5, 27.6, 27.4, 25.1, 22.4, 19.6, 18.3, 18.1, 18.0, 17.9 ppm, FT-IR: ν/cm^{-1} = 890, 931, 1022, 1184, 1249, 1370, 1482, 1532, 1654, 3295, mp: 82 – 86 °C, $[\alpha]_D^{20}$ = +30 (*c* 1.00, DMSO), MS (ESI) *m/z* calculated for $[C_{24}H_{34}ClN_8O_6S]^+$ ($[M+H]^+$): 597.2, found 597.1. Purity: 99%.

14d, (2*S*,3*S*)-2-(2-Acetamidoacetamido)-*N*-((*S*)-1-(((*S*)-5-guanidino-1-(6-bromobenzo [d]thiazol-2-yl)-1-oxopentan-2-yl)amino)-1-oxopropan-2-yl)-3-hydroxybutanamide

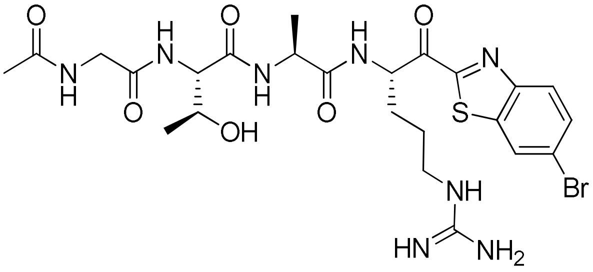

After the coupling of the respective peptide backbone **3** with the P1-warhead moiety **12d**, the deprotection of the Pbf- and O^{t}Bu-protecting group was performed in a TFA/DCM solution (50%, 2 mL) and stirred for 2 h at room temperature. The solution was evaporated under reduced pressure and purified by preparative HPLC (12.8 mg, 0.02 mmol, 4%). ^{1}H NMR (600 MHz, DMSO-d_6): δ/ppm = 8.61 – 8.56 (m, 1H), 8.48 – 8.30 (m, 1H), 8.25 – 8.17 (m, 2H), 8.09 – 7.97 (m, 1H), 7.90 – 7.74 (m, 2H), 7.54 (q, *J* = 5.9 Hz, 1H), 5.47 – 5.36 (m, 1H), 5.06 – 4.93 (m, 1H), 4.39 – 4.30 (m, 1H), 4.21 – 4.11 (m, 1H), 4.03 – 3.89 (m, 1H), 3.82 – 3.67 (m, 2H), 3.15 – 3.09 (m, 2H), 2.00 – 1.91 (m, 1H), 1.87 – 1.83 (m, 3H), 1.75 – 1.53 (m, 3H), 1.24 – 1.17 (m, 3H), 1.04 – 0.98 (m, 3H) ppm, ^{13}C NMR (151 MHz, DMSO-d_6): δ/ppm = 192.9, 172.5, 172.3, 170.0, 169.8, 169.6, 169.5, 169.4, 169.3, 165.3, 165.2, 158.5, 158.3, 158.1, 157.9, 156.6, 151.9, 138.2, 130.9, 126.8, 125.9, 121.4, 118.2, 116.2, 66.7, 66.5, 66.3, 58.5, 58.1, 58.0, 54.3, 54.1, 48.3, 48.1, 47.8, 42.3, 42.1, 40.2, 27.6, 27.4, 25.1, 22.4, 19.6, 18.3, 18.1, 18.0, 17.9 ppm, FT-IR: ν/cm^{-1} = 722, 888, 923, 1030, 1194, 1374, 1484, 1537, 1644, 3281, mp: 78 – 82 °C, $[\alpha]_D^{20}$ = +22 (*c* 1.00, DMSO), MS (ESI) *m/z* calculated for $[C_{24}H_{34}BrN_8O_6S]^+$ ($[M+H]^+$): 641.1, found 643.1. Purity: 99%.

15, (2*S*,3*S*)-2-(2-Acetamidoacetamido)-*N*-((*S*)-1-(((*S*)-5-guanidino-1-oxo-1-(4,5,6,7-tetrahydrobenzo[d]thiazol-2-yl)pentan-2-yl)amino)-1-oxopropan-2-yl)-3-hydroxybutanamide

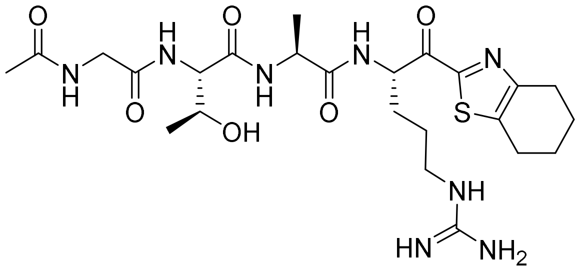

After the coupling of the respective peptide backbone **3** with the P1-warhead moiety **13**, the deprotection of the Pbf- and O^{t}Bu-protecting group was performed in a TFA/DCM solution (50%, 4 mL) and stirred for 2 h at room temperature. The solution was evaporated under reduced pressure and purified by preparative HPLC (16.9 mg, 0.03 mmol, 8%). ^{1}H NMR (600 MHz, DMSO-*d*$_6$): δ/ppm = 8.67 – 8.55 (m, 1H), 8.48 – 8.42 (m, 1H), 8.31 – 8.21 (m, 1H), 8.09 (d, *J* = 7.6 Hz, 1H), 7.89 (dd, *J* = 55.4, 8.2 Hz, 1H), 7.62 (s, 3H), 5.33 – 5.22 (m, 2H), 4.37 – 4.28 (m, 1H), 4.25 – 4.21 (m, 1H), 4.05 – 3.94 (m, 1H), 3.82 – 3.68 (m, 2H), 3.05 (p, *J* = 8.2, 7.4 Hz, 2H), 2.91 – 2.78 (m, 4H), 1.88 – 1.77 (m, 8H), 1.69 – 1.49 (m, 3H), 1.26 – 1.20 (m, 3H), 1.03 (d, *J* = 6.4 Hz, 3H) ppm, ^{13}C NMR (151 MHz, DMSO-*d*$_6$): δ/ppm = 191.2, 172.4, 170.0, 169.8, 169.5, 169.3, 167.4, 160.4, 157.2, 153.3, 138.8, 66.5, 58.0, 53.9, 48.3, 47.8, 42.1, 28.1, 26.4, 25.0, 23.6, 22.4, 19.5, 18.0 ppm, FT-IR: ν/cm^{-1} =773, 886, 939, 1026, 1231, 1369, 1428, 1532, 1642, 2937, mp: 110 – 114 °C, $[\alpha]_D^{20}$ = +27 (*c* 1.00, DMSO), MS (ESI) *m/z* calculated for $[C_{24}H_{39}N_8O_6S]^+$ ($[M+H]^+$): 567.6, found 567.1. Purity: 95%.

20, (2*S*,3*S*)-2-(2-Acetamidoacetamido)-*N*-((*S*)-1-(((*S*)-1-(benzo[d]thiazol-2-yl)-6-guanidino-1-oxohexan-2-yl)amino)-1-oxopropan-2-yl)-3-hydroxybutanamide

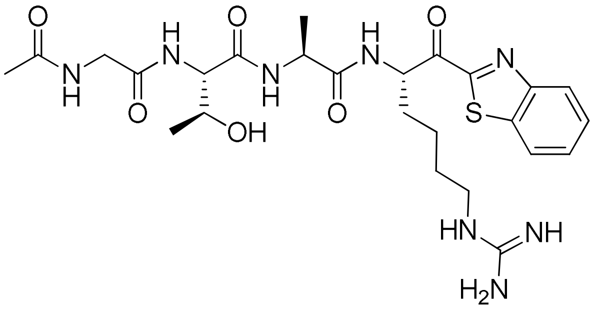

After the coupling of the respective peptide backbone **3** with the P1-warhead moiety **19**, the deprotection of the Cbz- and O^{t}Bu-protecting groups was performed in a TFA/TA solution (5:1, 10 mL) and stirred for 12 h at room temperature. The solution was evaporated under reduced pressure and purified by preparative HPLC (17.3 mg, 0.03 mmol, 8%). ^{1}H NMR (600 MHz, DMSO-*d*$_6$): δ/ppm = 8.54 – 8.33 (m, 1H), 8.25 (m, 2H), 8.18 (m, 1H), 8.06 – 7.93 (m, 1H), 7.87 – 7.54 (m, 4H), 5.56 – 5.39 (m, 1H), 4.44 – 4.31 (m, 1H), 4.25 – 4.13 (m, 1H), 4.05 – 3.92 (m, 1H), 3.80 – 3.69 (m, 2H), 3.18 – 2.98 (m, 2H), 1.95 (s, 1H), 1.89 – 1.83 (m, 3H), 1.81 – 1.34 (m, 6H), 1.28 – 1.19 (m, 3H), 1.06 – 0.98 (m, 3H), ^{13}C NMR (151 MHz, DMSO-*d*$_6$): δ/ppm = 193.3, 172.6, 169.9, 169.8, 169.7, 169.6, 169.5, 169.4, 164.5, 156.8, 153.0, 136.4, 128.3, 127.6, 126.6, 125.3, 123.3, 66.7, 54.7, 44.6, 42.2, 30.0, 28.0, 22.7, 22.4, 19.6, 19.5, 18.2, 18.0, FT-IR: ν/cm^{-1} = 668, 722, 762, 800, 1130, 1203, 1482, 1549, 1634, 3284, mp: 105 – 110 °C, $[\alpha]_D^{20}$ = +31 (*c* 1.00, DMSO), MS (ESI) *m/z* calculated for $[C_{25}H_{37}N_8O_6S]^+$ ($[M+H]^+$): 577.6, found 577.3. Purity: 98%.

29, (2*S*,3*S*)-2-(2-Acetamidoacetamido)-*N*-((*S*)-1-(((*S*)-1-(benzo[d]thiazol-2-yl)-3-(4-guanidinophenyl)-1-oxopropan-2-yl)amino)-1-oxopropan-2-yl)-3-hydroxybutanamide

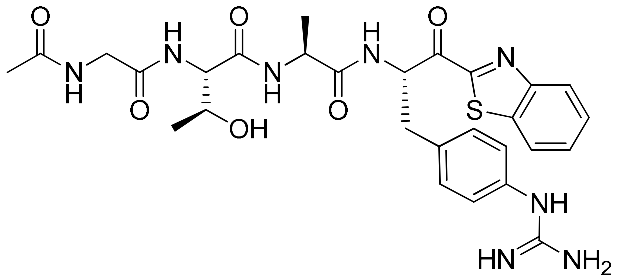

After the coupling of the respective peptide backbone **3** with the P1-warhead moiety **28**, the deprotection of the Cbz- and O^{t}Bu-protecting groups was performed in a TFA/TA solution (5:1, 10 mL) and stirred for 12 h at room temperature. The solution was evaporated under reduced pressure and purified by preparative HPLC (42.7 mg, 0.07 mmol, 25%). ^{1}H NMR (600 MHz, DMSO-*d*$_6$): δ/ppm = 8.65 – 8.51 (m, 2H), 8.31 – 8.25 (m, 2H), 8.16 (m, 1H), 7.97 – 7.90 (m, 2H), 7.70 – 7.66 (m, 2H), 7.52 (s, 4H), 7.42 – 7.37 (m, 2H), 7.20 – 7.16 (m, 2H), 5.74 – 5.67 (m, 1H), 4.98 (s, 1H), 4.38 – 4.12 (m, 3H), 4.04 – 3.90 (m, 1H), 3.75 (dd, 2H), 2.99 – 2.88 (m, 1H), 1.85 (m, 3H), 1.13 (m, 3H), 1.02 – 0.98 (m, 3H), ^{13}C NMR (151 MHz, DMSO-*d*$_6$): δ/ppm = 192.4, 172.6, 172.3, 170.0, 169.8, 169.6, 169.4, 164.2, 159.2, 158.8, 155.8, 152.9, 136.4, 135.6, 133.8, 130.4, 128.3, 127.6, 125.3, 124.4, 123.2, 58.2, 47.8, 42.1, 35.7, 22.4, 19.5, 18.2, 18.0, FT-IR: ν/cm^{-1} = 668, 721, 762, 801, 1132, 1183, 1202, 1515, 1552, 1645, mp: 125 – 133 °C, $[\alpha]_D^{20}$ = −23 (*c* 1.00, DMSO), MS (ESI) *m/z* calculated for $[C_{28}H_{35}N_8O_6S]^+$ ($[M+H]^+$): 611.2, found 611.2. Purity: 97%.

45, (2*S*,3*S*)-2-(2-Acetamidoacetamido)-*N*-((*S*)-1-(((*S*)-1-(benzo[d]thiazol-2-yl)-3-(3-guanidinophenyl)-1-oxopropan-2-yl)amino)-1-oxopropan-2-yl)-3-hydroxybutanamide

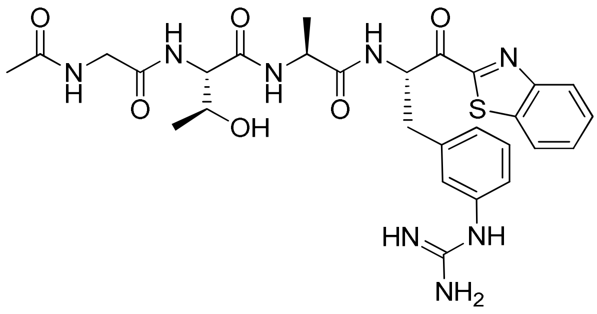

After the coupling of the respective peptide backbone **3** with the P1-warhead moiety **44**, the deprotection of the Cbz- and O^{t}Bu-protecting groups was performed in a TFA/TA solution (5:1, 10 mL) and stirred for 12 h at room temperature. The solution was evaporated under reduced pressure and purified by preparative HPLC (36.6 mg, 0.06 mmol, 40%). ^{1}H NMR (600 MHz, DMSO-*d*$_6$): δ/ppm = 8.52 (dd, *J* = 13.2, 7.4 Hz, 1H), 8.44 (d, *J* = 1.2 Hz, 1H), 8.33 – 8.28 (m, 2H), 8.21 (dd, *J* = 26.0, 5.8 Hz, 1H), 8.01 – 7.89 (m, 4H), 7.71 – 7.66 (m, 2H), 7.35 – 7.30 (m, 1H), 7.23 – 7.14 (m, 2H), 7.08 – 7.02 (m, 1H), 5.77 – 5.67 (m, 1H), 5.28 (s, 0H), 5.03 (s, 1H), 4.33 – 4.11 (m, 3H), 3.99 – 3.91 (m, 1H), 3.78 – 3.68 (m, 2H), 2.96 – 2.91 (m, 1H), 1.87 – 1.83 (m, 3H), 1.17 – 1.08 (m, 3H), 1.00 (t, *J* = 6.2 Hz, 3H), ^{13}C NMR (151 MHz, DMSO-*d*$_6$): δ/ppm = 192.4, 172.5, 172.4, 170.0, 169.7, 169.2, 167.2, 164.2, 156.1, 152.9, 138.9, 136.4, 129.6, 128.3, 127.6, 125.4, 124.6, 123.3, 122.2, 66.6, 57.7, 56.5, 48.0, 42.1, 36.2, 22.4, 19.7, 18.1, FT-IR: ν/cm^{-1} = 751, 886, 1023, 1130, 1251, 1347, 1473, 1512, 1580, 2783, mp: 103 – 106 °C, $[\alpha]_D^{20}$ = +18 (*c* 1.00, DMSO), MS (ESI) *m/z* calculated for $[C_{28}H_{35}N_8O_6S]^+$ ($[M+H]^+$): 611.2, found 611.1. Purity: 96%.

38, (2*S*,3*S*)-2-(2-Acetamidoacetamido)-*N*-((*S*)-1-(((*S*)-1-(benzo[d]thiazol-2-yl)-3-(4-guanidinocyclohexyl)-1-oxopropan-2-yl)amino)-1-oxopropan-2-yl)-3-hydroxybutanamide

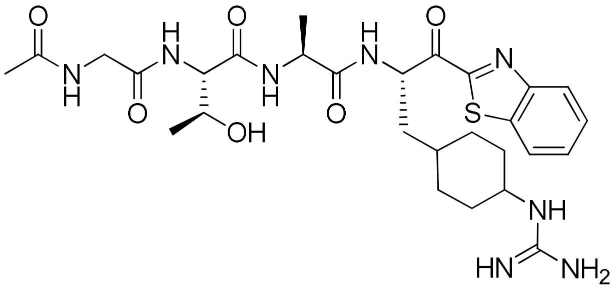

After the coupling of the respective peptide backbone **3** with the P1-warhead moiety **37**, the deprotection of the Cbz- and O^{t}Bu-protecting groups was performed in a TFA/TA solution (5:1, 10 mL) and stirred for 12 h at room temperature. The solution was evaporated under reduced pressure and purified by preparative HPLC (24.6 mg, 0.04 mmol, 20%). ^{1}H NMR (600 MHz, DMSO-*d*$_6$): *δ*/ppm = 8.42 – 8.39 (m, 1H), 8.30 – 8.21 (m, 2H), 8.07 (d, *J* = 7.6 Hz, 1H), 8.02 – 7.89 (m, 1H), 7.79 (d, *J* = 8.2 Hz, 1H), 7.71 – 7.65 (m, 2H), 5.63 – 5.51 (m, 1H), 5.19 – 5.05 (m, 1H), 4.39 – 4.16 (m, 2H), 4.05 – 3.93 (m, 1H), 3.84 – 3.66 (m, 2H), 3.29 – 3.22 (m, 1H), 1.96 – 1.38 (m, 9H), 1.36 – 1.06 (m, 5H), 1.06 – 1.00 (m, 3H), ^{13}C NMR (151 MHz, DMSO-*d*$_6$): *δ*/ppm = 193.7, 172.5, 170.0, 169.4, 167.2, 164.5, 157.9, 155.9, 152.9, 136.4, 135.9, 128.3, 127.7, 125.2, 123.3, 66.6, 58.0, 52.7, 52.5, 49.7, 48.3, 47.8, 42.3, 32.9, 29.9, 22.4, 19.6, 18.1, FT-IR: *ν*/cm^{-1} = 657, 668, 699, 730, 764, 1139, 1203, 1551, 1655, 2343, mp: 148 – 155 °C, $[\alpha]_D^{20}$ = –25 (*c* 1.00, DMSO), MS (ESI) *m/z* calculated for $[C_{28}H_{41}N_8O_6S]^+$ ($[M+H]^+$): 617.3, found 617.3. Purity: 97%.

Synthesis of TMPRSS2 targeting compounds

30, (*S*)-1-((*S*)-2-Acetamido-4-amino-4-oxobutanoyl)-*N*-((*S*)-1-(benzo[d]thiazol-2-yl)-3-(4-guanidinophenyl)-1-oxopropan-2-yl)pyrrolidine-2-carboxamide

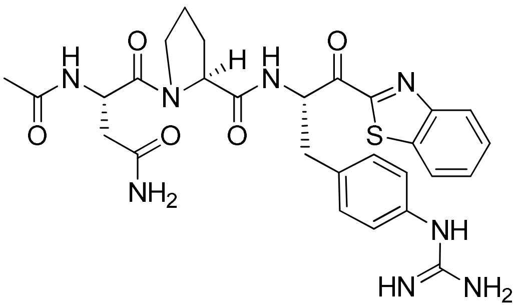

After the coupling of the respective peptide backbone **21** with the P1-warhead moiety **28**, the deprotection of the Cbz- and O^{t}Bu-protecting groups was performed in a TFA/TA solution (5:1, 10 mL) and stirred for 12 h at room temperature. The solution was evaporated under reduced pressure and purified by preparative HPLC (50.0 mg, 0.08 mmol, 19%). ^{1}H NMR (600 MHz, DMSO-*d*$_6$): *δ*/ppm = δ 8.45 – 8.41 (m, 1H), 8.39 – 8.37 (m, 1H), 8.32 – 8.26 (m, 2H), 7.85 (s, 2H), 7.72 – 7.64 (m, 2H), 7.63 – 7.59 (m, 1H), 7.46 – 7.41 (m, 1H), 7.34 (d, *J* = 8.3 Hz, 1H), 7.20 – 7.10 (m, 2H), 7.08 – 7.05 (m, 1H), 5.71 – 5.54 (m, 1H), 4.81 – 4.75 (m, 1H), 4.29 (ddd, *J* = 7.9, 4.8, 2.4 Hz, 1H), 3.78 – 3.64 (m, 2H), 3.33 – 3.28 (m, 2H), 3.06 – 2.96 (m, 1H), 2.71 – 2.59 (m, 1H), 2.43 – 2.34 (m, 1H), 1.96 – 1.61 (m, 7H), ^{13}C NMR (151 MHz, DMSO-*d*$_6$): *δ*/ppm = 192.6, 172.3, 172.1, 171.7, 171.5, 170.6, 170.3, 168.9, 167.7, 164.4, 164.1, 156.1, 153.0, 136.4, 130.3, 128.3, 127.6, 125.3, 124.0, 123.7, 59.7, 59.2, 56.8, 47.0, 37.6, 34.9, 29.5, 23.6, 22.3, FT-IR: *ν*/cm^{-1} = 759, 877, 1021, 1127, 1203, 1313, 1375, 1420, 1619, 2364, mp: 200 – 203 °C, $[\alpha]_D^{20}$ = –59 (*c* 1.00, DMSO), MS (ESI) *m/z* calculated for $[C_{28}H_{33}N_8O_5S]^+$ ($[M+H]^+$): 593.2, found 593.1. Purity: 97%.

39, (*S*)-1-((*S*)-2-Acetamido-4-amino-4-oxobutanoyl)-*N*-((*S*)-1-(benzo[d]thiazol-2-yl)-3-(4-guanidinocyclohexyl)-1-oxopropan-2-yl)pyrrolidine-2-carboxamide

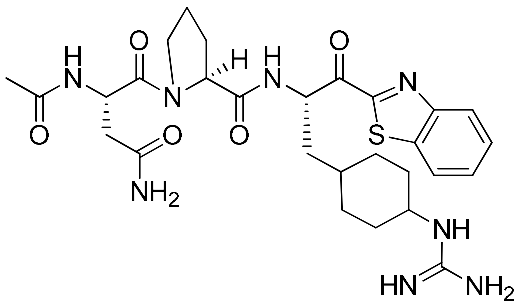

After the coupling of the respective peptide backbone **21** with the P1-warhead moiety **37**, the deprotection of the Cbz- and O^tBu-protecting groups was performed in a TFA/TA solution (5:1, 10 mL) and stirred for 12 h at room temperature. The solution was evaporated under reduced pressure and purified by preparative HPLC (27.8 mg, 0.05 mmol, 10%). ^{1}H NMR (600 MHz, DMSO-d_6): δ/ppm = δ 8.40 – 8.31 (m, 1H), 8.29 – 8.20 (m, 2H), 8.17 – 8.13 (m, 1H), 7.70 – 7.65 (m, 2H), 7.59 – 7.47 (m, 1H), 7.43 – 7.36 (m, 1H), 5.67 – 5.58 (m, 1H), 5.52 – 5.38 (m, 1H), 4.80 – 4.74 (m, 1H), 4.35 – 4.28 (m, 1H), 3.86 – 3.55 (m, 3H), 3.32 – 3.25 (m, 1H), 2.74 – 2.60 (m, 1H), 2.43 – 2.33 (m, 1H), 2.05 – 0.93 (m, 20H), ^{13}C NMR (151 MHz, DMSO-d_6): δ/ppm = 193.9, 172.8, 172.2, 170.6, 169.3, 165.1, 164.7, 158.6, 156.2, 153.3, 136.7, 128.6, 128.1, 125.5, 123.8, 118.7, 116.7, 60.4, 59.6, 53.3, 50.2, 47.7, 37.7, 33.4, 29.6, 24.1, 23.5, 22.7, FT-IR: v/cm^{-1} = 720, 835, 990, 1023, 1119, 1200, 1431, 1538, 1619, 2347, mp: 113 – 116 °C, $[\alpha]_D^{20}$ = −60 (*c* 1.00, DMSO), MS (ESI) *m/z* calculated for $[C_{28}H_{38}N_8O_5S]^+$ ($[M+H]^+$): 599.2, found 599.1. Purity: 98%.

31, (*S*)-2-Acetamido-*N*-((*S*)-1-(((*S*)-1-(benzo[d]thiazol-2-yl)-3-(4-guanidinophenyl)-1-oxopropan-2-yl)amino)-1-oxo-3-phenylpropan-2-yl)succinamide

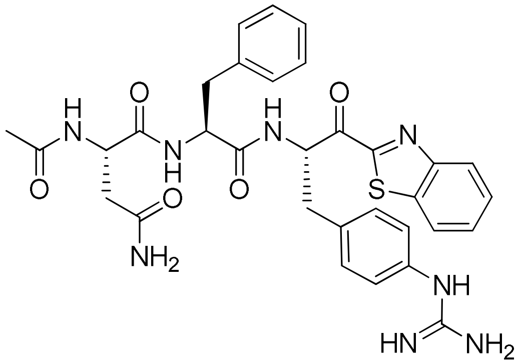

After the coupling of the respective peptide backbone **22** with the P1-warhead moiety **28**, the deprotection of the Cbz- and O^tBu-protecting groups was performed in a TFA/TA solution (5:1, 10 mL) and stirred for 12 h at room temperature. The solution was evaporated under reduced pressure and purified by preparative HPLC (20.5 mg, 0.03 mmol, 11%). ^{1}H NMR (600 MHz, DMSO-d_6): δ/ppm = 8.80 – 8.69 (m, 1H), 8.34 – 8.29 (m, 1H), 8.13 – 7.89 (m, 2H), 7.75 – 7.68 (m, 2H), 7.49 – 7.36 (m, 6H), 7.33 – 7.25 (m, 1H), 7.21 – 7.14 (m, 5H), 7.03 (ddt, *J* = 5.9, 4.4, 1.7 Hz, 1H), 6.97 – 6.92 (m, 1H), 5.79 – 5.71 (m, 1H), 4.55 – 4.44 (m, 2H), 3.10 – 2.61 (m, 4H), 2.35 – 2.13 (m, 2H), 1.84 – 1.73 (m, 3H), ^{13}C NMR (151 MHz, DMSO-d_6): δ/ppm = 192.5, 171.6, 171.3, 169.4, 164.3, 164.2, 155.7, 153.0, 137.5, 136.4, 136.0, 133.9, 130.4, 129.2, 128.4, 128.0, 127.7, 126.3, 125.4, 124.4, 123.8, 118.2, 116.3, 56.4, 53.7, 53.4, 49.9, 49.5, 37.3, 35.5, 22.5, FT-IR: v/cm^{-1} = 700, 728, 779, 877, 1122, 1195, 1377, 1552, 1656, 2358, mp: 136 – 138 °C, $[\alpha]_D^{20}$ = −57 (*c* 1.00, DMSO), MS (ESI) *m/z* calculated for $[C_{32}H_{35}N_8O_5S]^+$ ($[M+H]^+$): 643.2, found 643.1. Purity: 96%.

32, (*S*)-2-Acetamido-*N*-((*S*)-1-(((*S*)-1-(benzo[d]thiazol-2-yl)-3-(4-guanidinophenyl)-1-oxopropan-2-yl)amino)-1-oxo-3-cyclohexylpropan-2-yl)succinamide

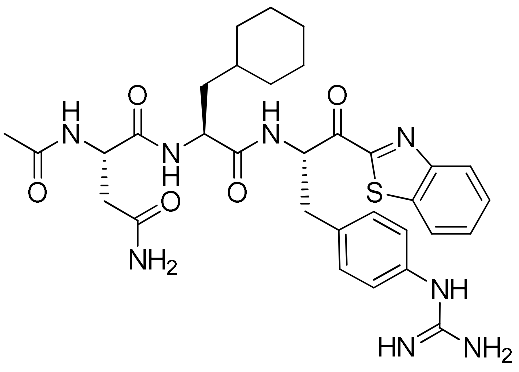

After the coupling of the respective peptide backbone **23** with the P1-warhead moiety **28**, the deprotection of the Cbz- and O^{t}Bu-protecting groups was performed in a TFA/TA solution (5:1, 10 mL) and stirred for 12 h at room temperature. The solution was evaporated under reduced pressure and purified by preparative HPLC (35.2 mg, 0.05 mmol, 19%). ^{1}H NMR (600 MHz, DMSO-*d*$_6$): *δ*/ppm = 8.63 – 8.52 (m, 1H), 8.41 (s, 1H), 8.32 – 8.26 (m, 1H), 8.19 – 8.13 (m, 1H), 7.98 – 7.85 (m, 4H), 7.74 – 7.62 (m, 2H), 7.39 – 7.33 (m, 2H), 7.16 – 7.10 (m, 2H), 6.99 – 6.92 (m, 1H), 5.68 – 5.59 (m, 1H), 4.56 – 4.48 (m, 1H), 4.31 – 4.23 (m, 1H), 3.32 – 3.26 (m, 1H), 3.08 (s, 1H), 3.05 – 2.94 (m, 1H), 2.48 – 2.32 (m, 2H), 1.87 – 1.75 (m, 3H), 1.60 – 1.00 (m, 11H), 0.82 – 0.68 (m, 2H), ^{13}C NMR (151 MHz, DMSO-*d*$_6$): *δ*/ppm = 192.4, 172.4, 171.6, 171.1, 169.6, 169.3, 167.6, 164.2, 161.5, 156.1, 155.1, 153.6, 152.9, 143.7, 136.4, 135.1, 130.3, 129.7, 128.3, 127.6, 125.4, 124.2, 123.7, 123.3, 56.6, 49.8, 37.0, 35.2, 33.1, 31.5, 26.0, 22.6, FT-IR: ν/cm^{-1} =664, 754, 1015, 1203, 1377, 1512, 1538, 1636, 2355, 2920, mp: 153 – 155 °C, $[\alpha]_D^{20}$ = −60 (*c* 1.00, DMSO), MS (ESI) *m/z* calculated for $[C_{32}H_{41}N_8O_5S]^+$ ([M+H]$^+$): 649.2, found]$^+$): 649.2, Purity: 96%.

33, (*S*)-2-Acetamido-*N*-((*S*)-1-(benzo[d]thiazol-2-yl)-3-(4-guanidinophenyl)-1-oxopropan-2-yl)-3-phenylpropanamide

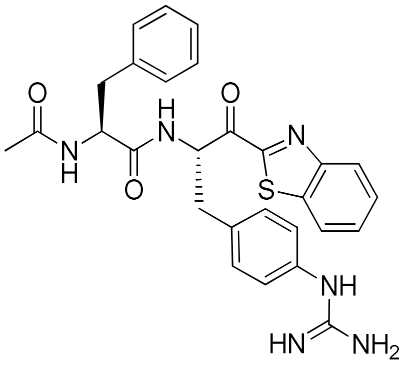

After the coupling of Ac-Phe-OH with the P1-warhead moiety **28**, the deprotection of the Cbz- and O^{t}Bu-protecting groups was performed in a TFA/TA solution (5:1, 10 mL) and stirred for 12 h at room temperature. The solution was evaporated under reduced pressure and purified by preparative HPLC (16.3 mg, 0.03 mmol, 28%). ^{1}H NMR (400 MHz, DMSO-*d*$_6$): *δ*/ppm = 8.76 (dd, *J* = 28.0, 7.4 Hz, 1H), 8.41 (s, 1H), 8.32 – 8.27 (m, 2H), 8.08 (dd, *J* = 8.5, 4.4 Hz, 1H), 7.91 (s, 2H), 7.72 – 7.64 (m, 2H), 7.38 – 7.31 (m, 2H), 7.23 – 7.11 (m, 8H), 5.77 – 5.68 (m, 0H), 4.60 – 4.50 (m, 1H), 3.32 – 3.29 (m, 1H), 3.00 – 2.52 (m, 3H), 1.73 – 1.66 (m, 3H), ^{13}C NMR (101 MHz, DMSO-*d*$_6$): *δ*/ppm = 193.0, 172.0, 169.6, 167.9, 164.7, 156.6, 153.4, 138.3, 136.9, 135.4, 129.5, 128.4, 128.1, 126.7, 125.8, 124.3, 123.7, 56.7, 54.2, 37.8, 36.1, 22.8, FT-IR: ν/cm^{-1} = 711, 773, 793, 832, 1125, 1195, 1397, 1510, 1636, 2355, mp: 140 – 143 °C, $[\alpha]_D^{20}$ = +57 (*c* 1.00, DMSO), MS (ESI) *m/z* calculated for $[C_{28}H_{29}N_6O_3S]^+$ ([M+H]$^+$): 529.1, found 529.0. Purity: 95%.

Spectra and chromatograms

14a, (2*S*,3*S*)-2-(2-Acetamidoacetamido)-*N*-((*S*)-1-(((*S*)-1-(benzo[*d*]thiazol-2-yl)-5-guanidino-1-oxopentan-2-yl)amino)-1-oxopropan-2-yl)-3-hydroxybutanamide

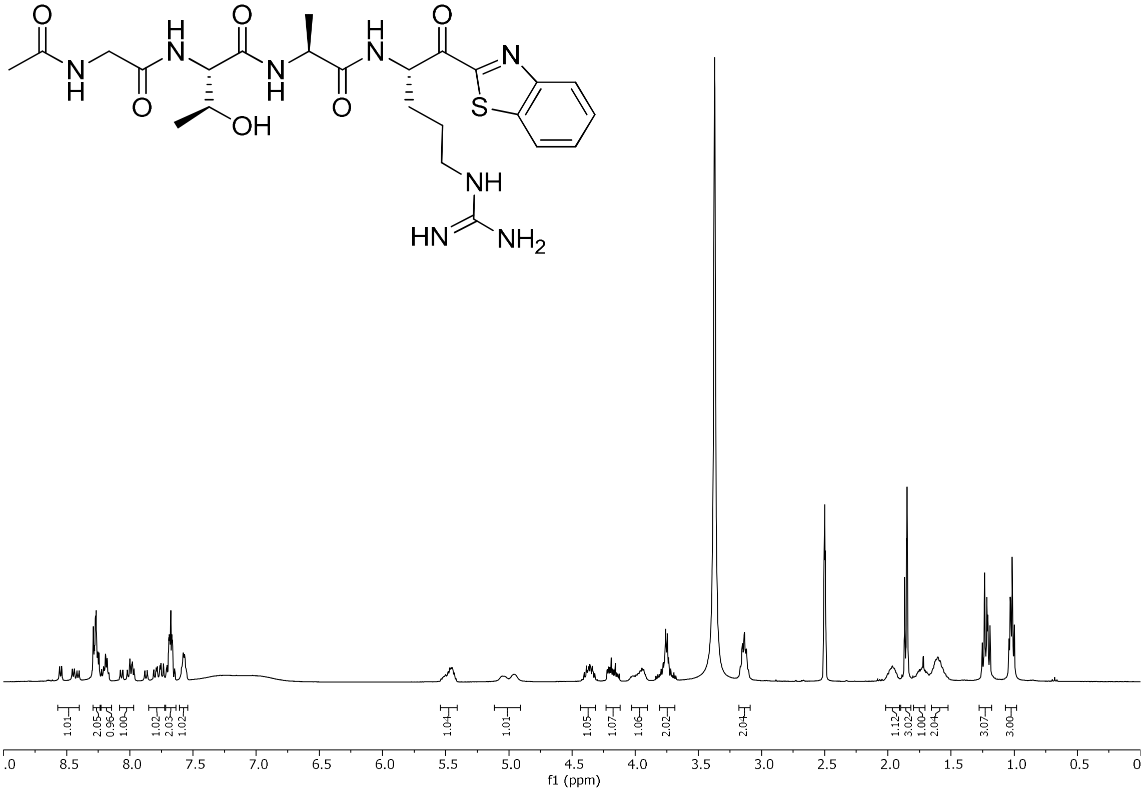

Figure S6a: ^{1}H NMR of **14a**.

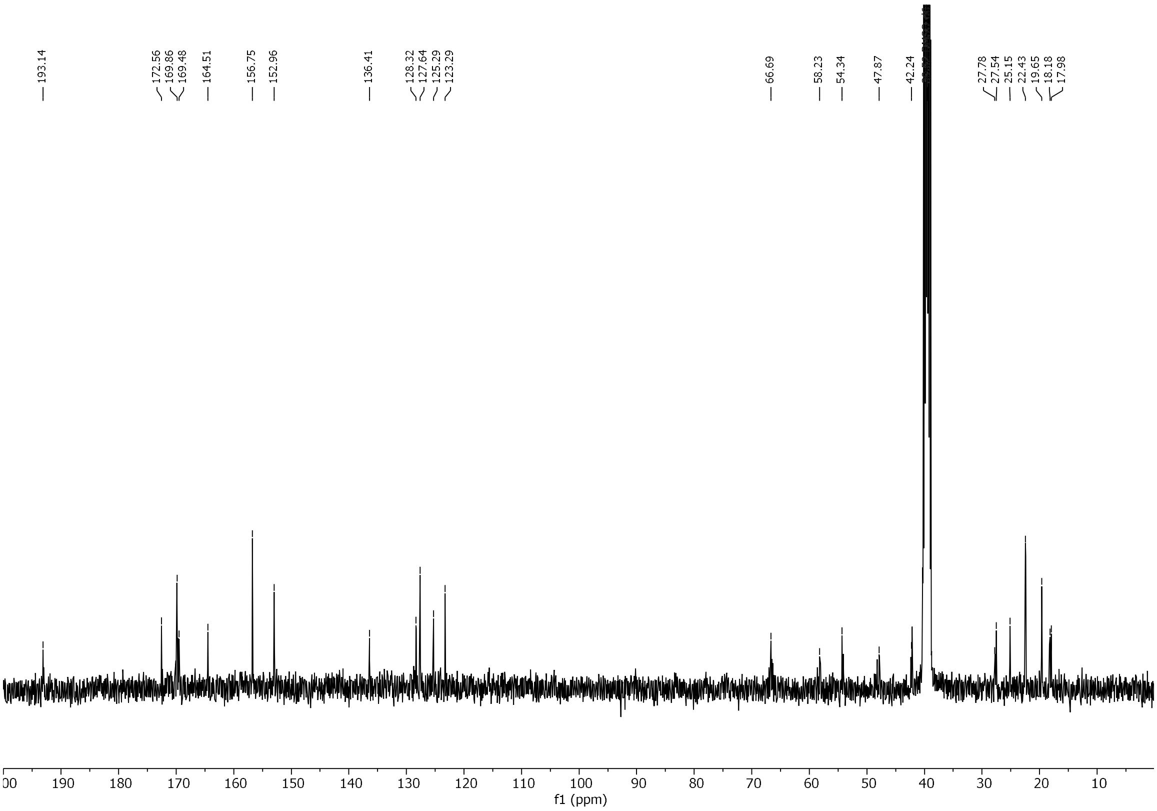

Figure S6b: ^{13}C NMR of **14a**.

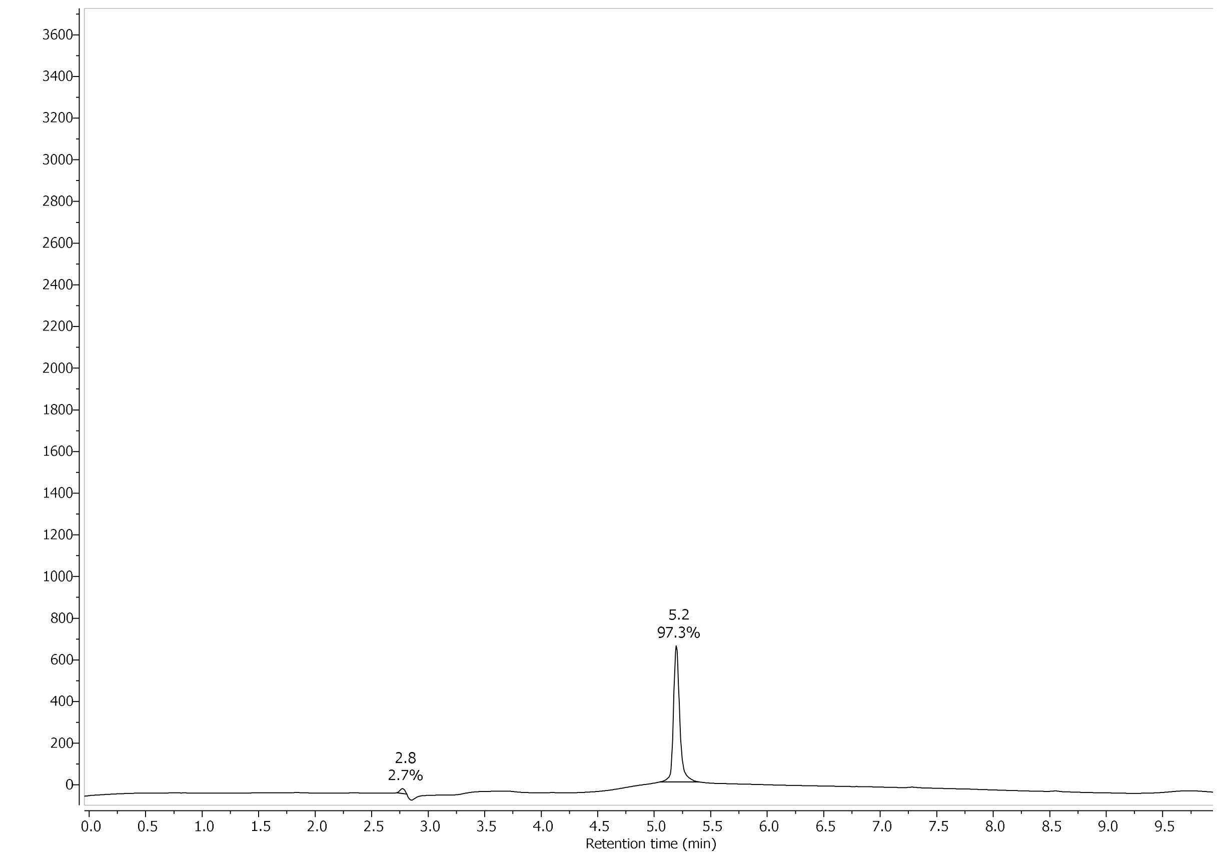

Figure S6c: HPLC Chromatogram of **14a** at 210 nm.

14f, (2*S*,3*S*)-2-(2-Acetamidoacetamido)-*N*-((*S*)-1-(((*S*)-1-(benzo[b]thiophen-2-yl)-5-guanidino-1-oxopentan-2-yl)amino)-1-oxopropan-2-yl)-3-hydroxybutanamide

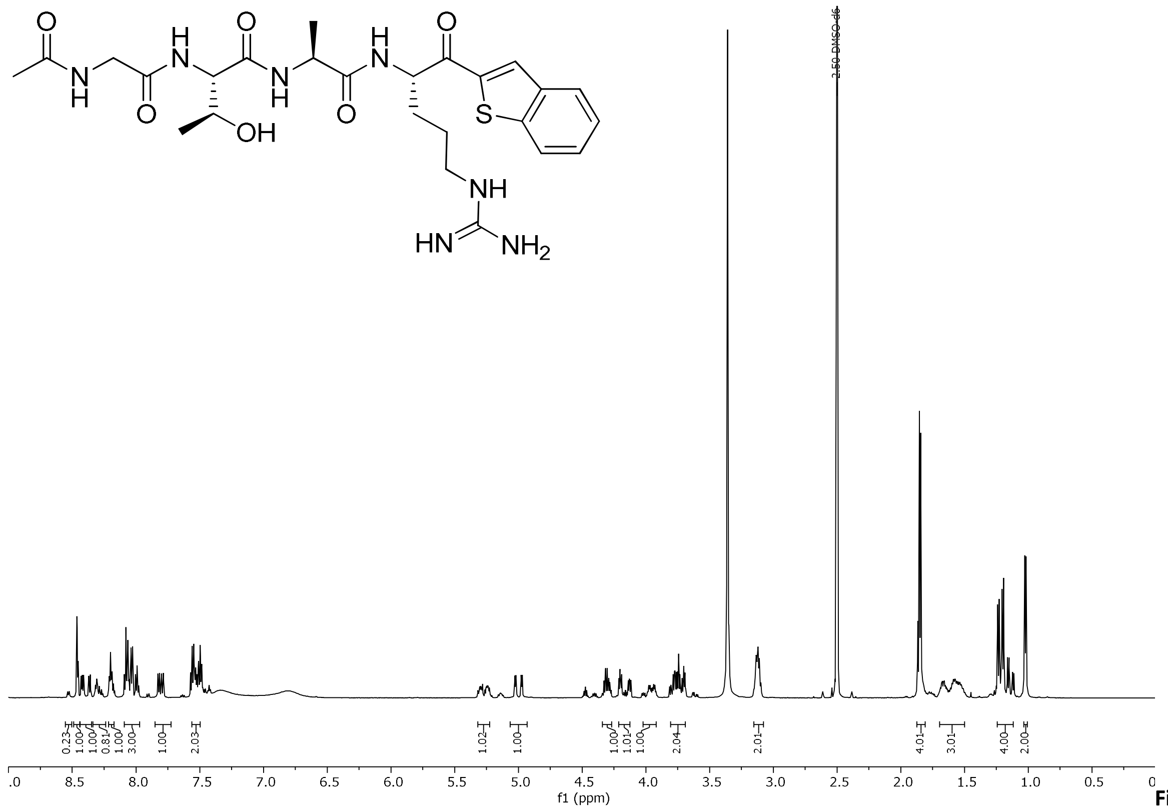

Figure S7a: ^{1}H NMR of **14f**.

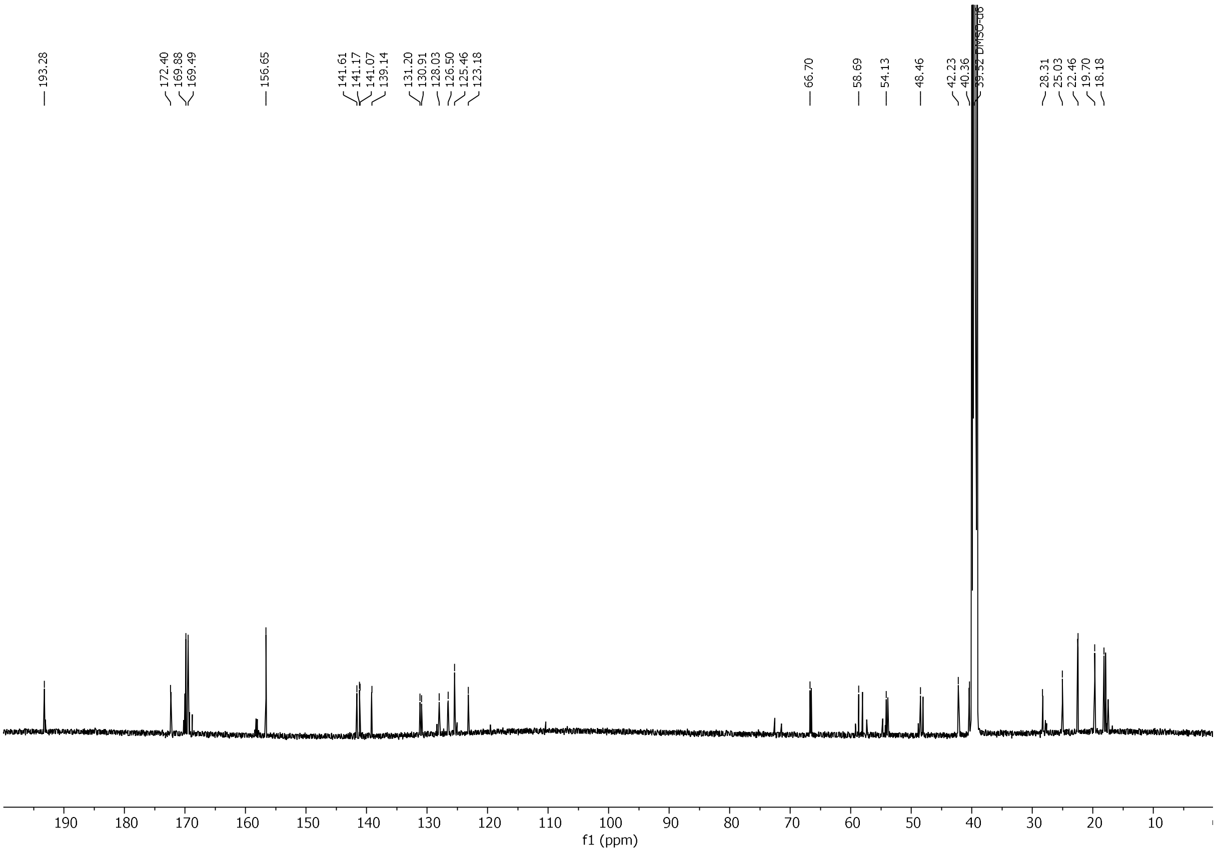

Figure S7b: ^{13}C NMR of **14f**.

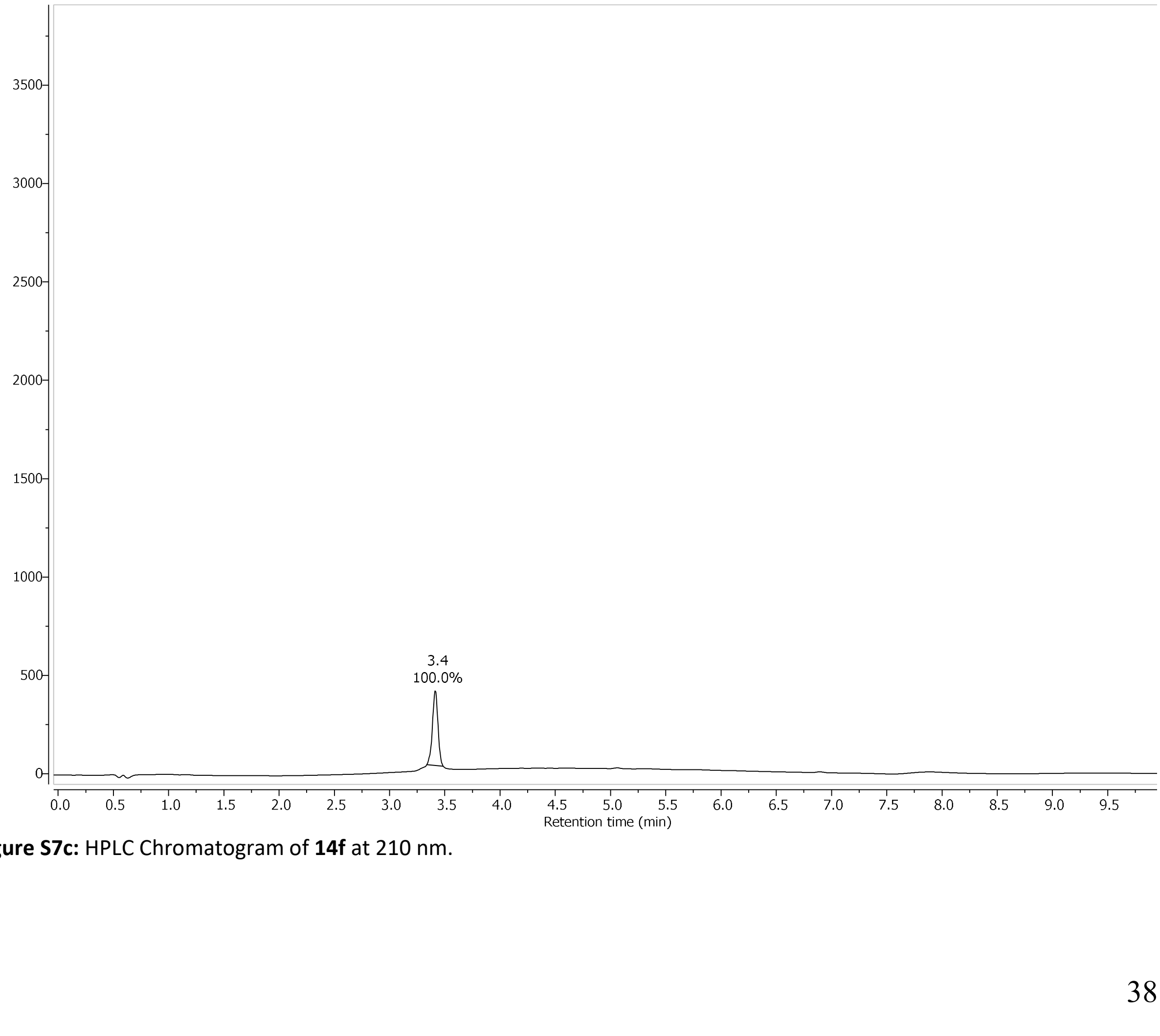

Figure S7c: HPLC Chromatogram of **14f** at 210 nm.

14e, (2*S*,3*S*)-2-(2-Acetamidoacetamido)-*N*-((*S*)-1-(((*S*)-5-guanidino-1-(6-methoxybenzo [d]thiazol-2-yl)-1-oxopentan-2-yl)amino)-1-oxopropan-2-yl)-3-hydroxybutanamide

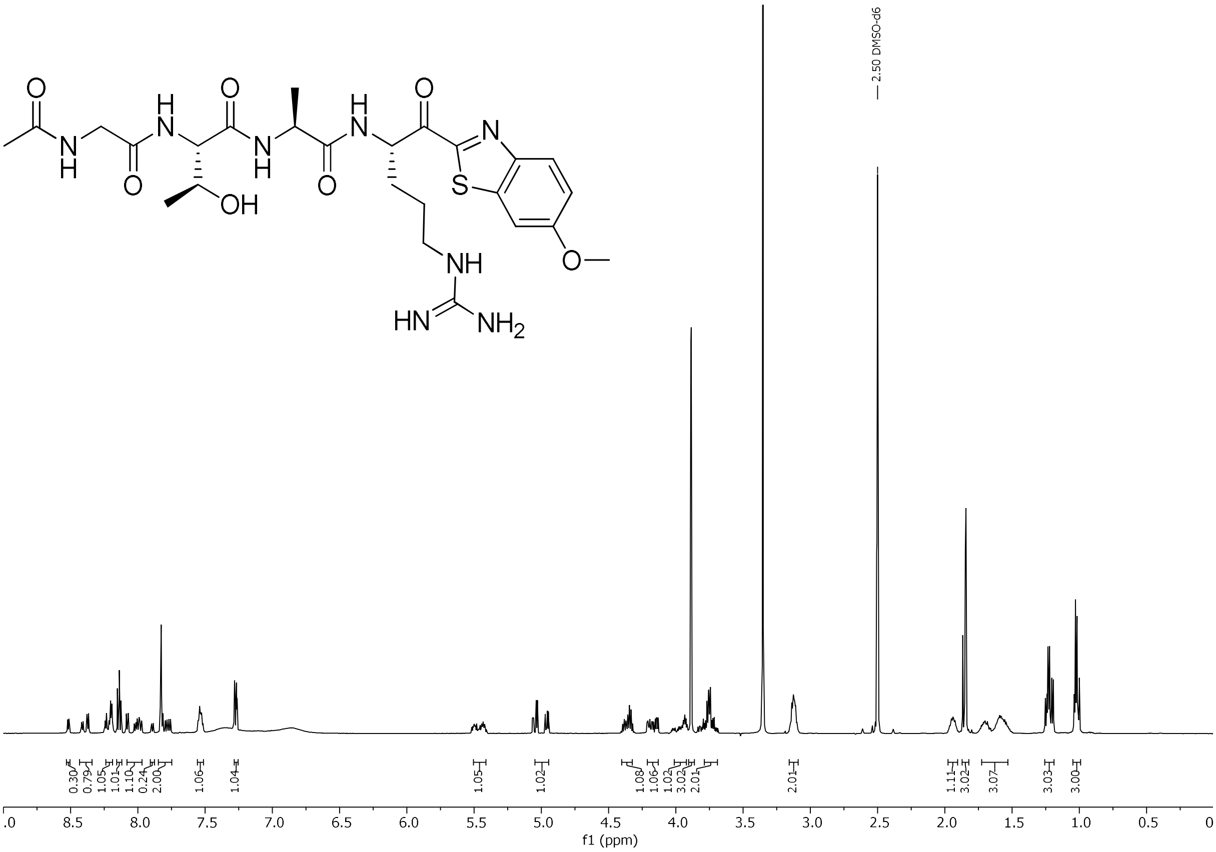

Figure S8a: ^{1}H NMR of **14e**.

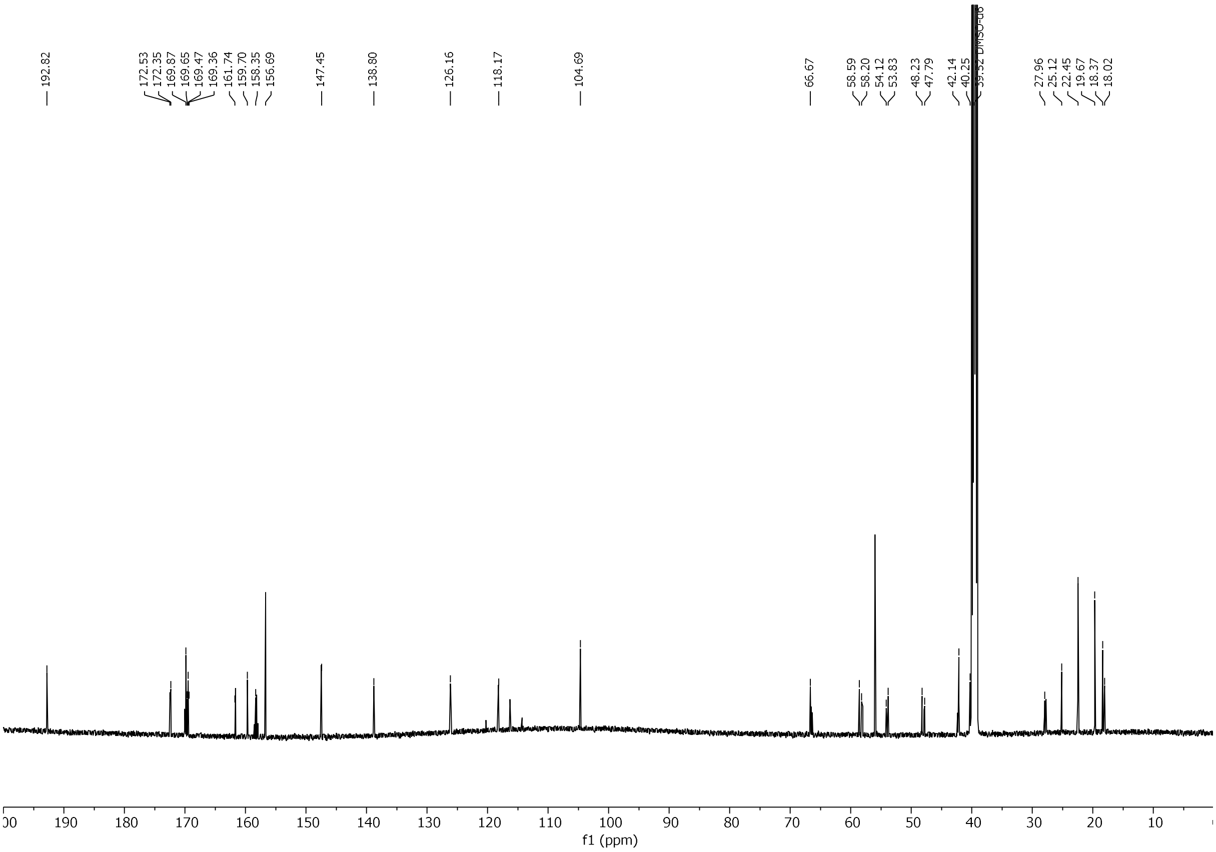

Figure S8b: ^{13}C NMR of **14e**.

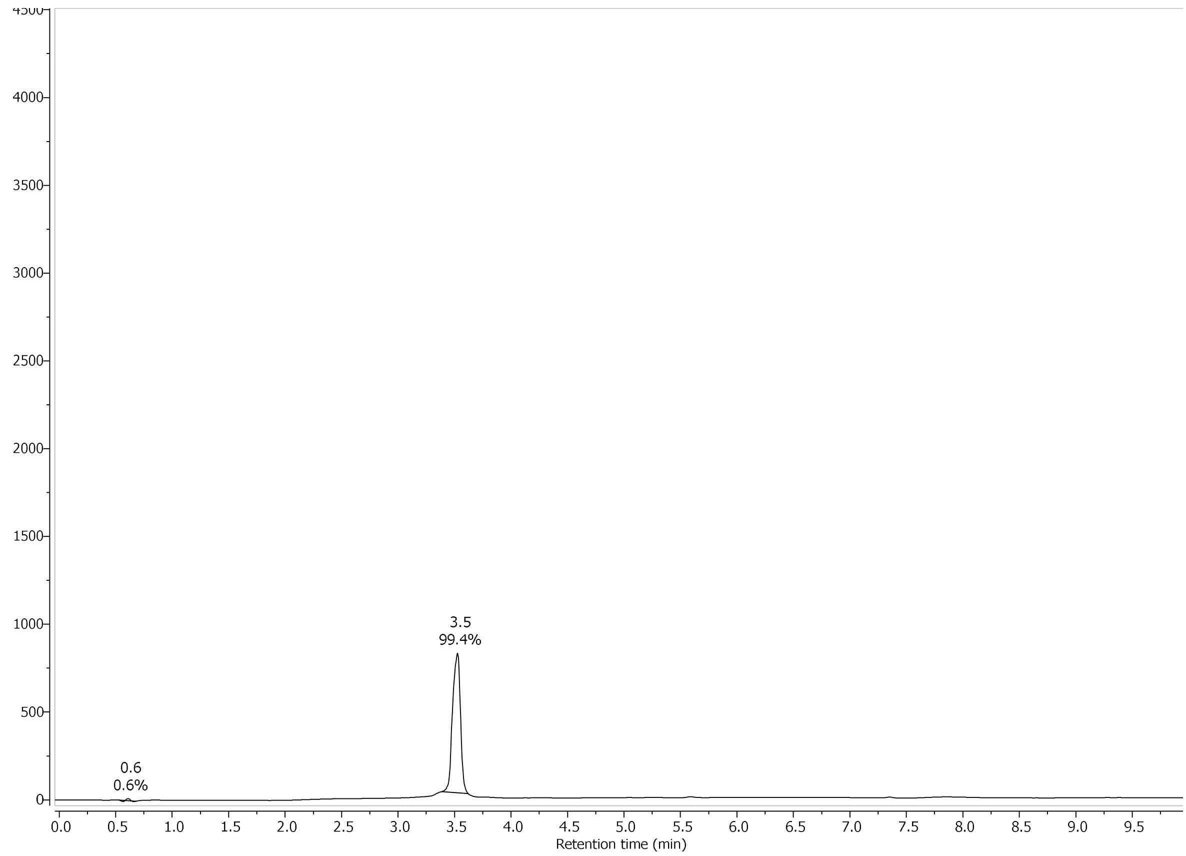

Figure S8c: HPLC Chromatogram of **14e** at 210 nm.

14b, (2*S*,3*S*)-2-(2-Acetamidoacetamido)-*N*-((*S*)-1-(((*S*)-5-guanidino-1-(6-fluorobenzo [d]thiazol-2-yl)-1-oxopentan-2-yl)amino)-1-oxopropan-2-yl)-3-hydroxybutanamide

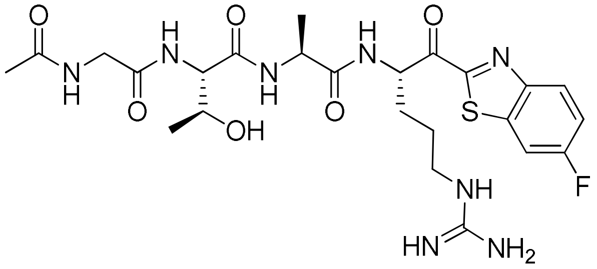

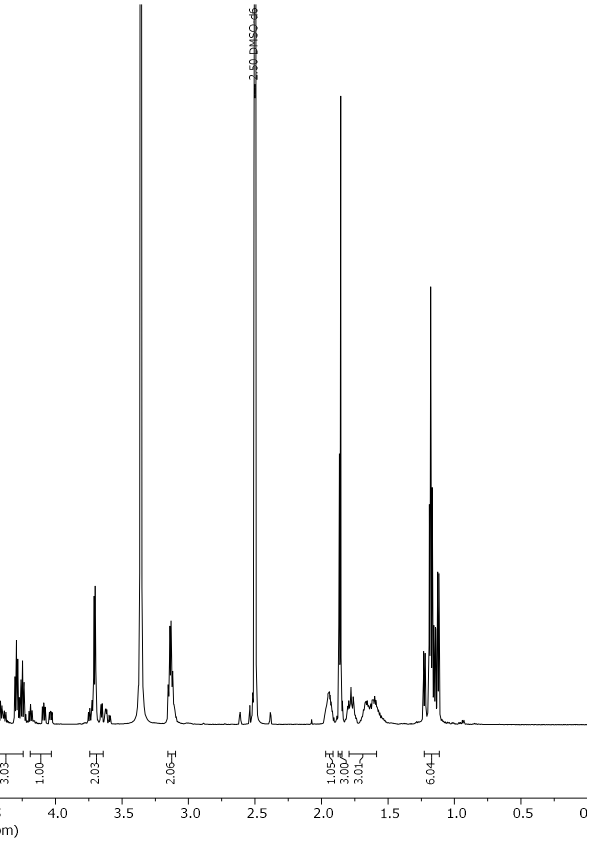

Figure S9a: ^{1}H NMR of **14b**.

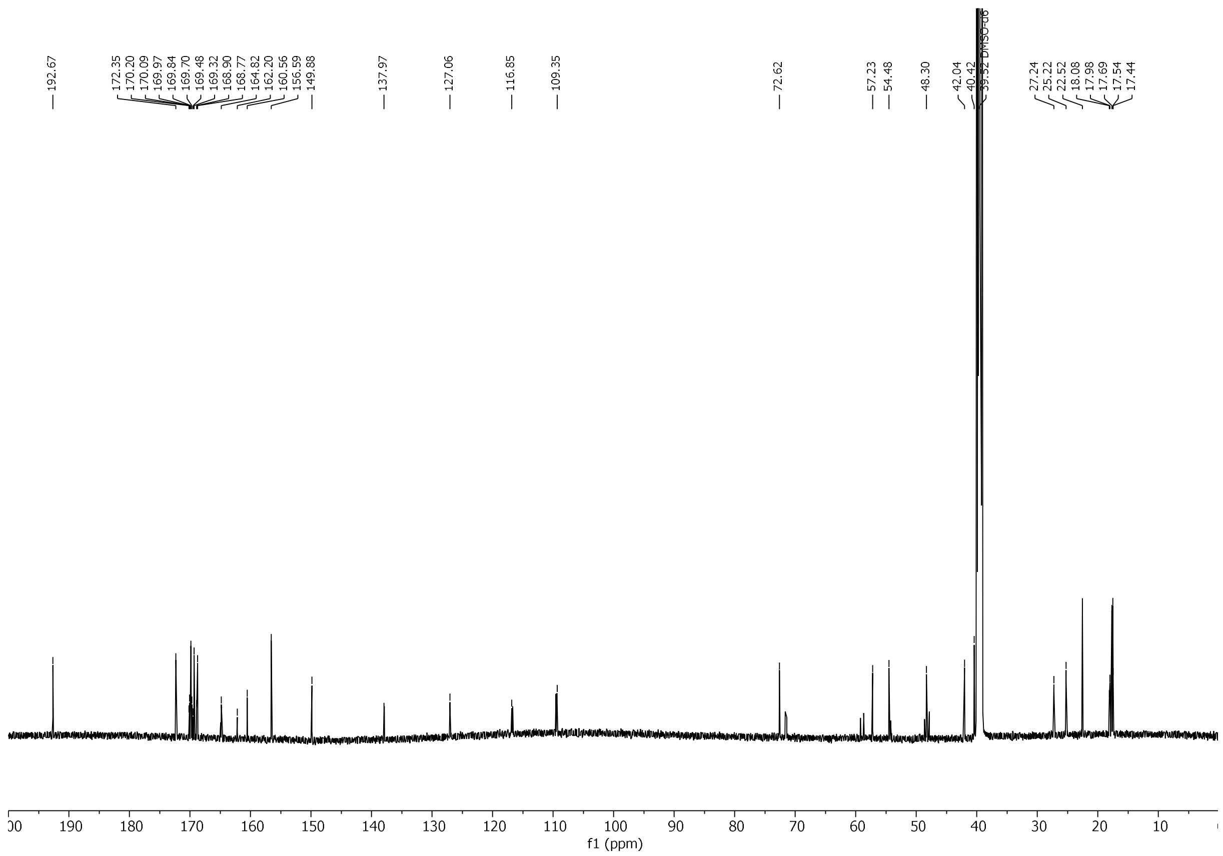

Figure S9b: ^{13}C NMR of **14b**.

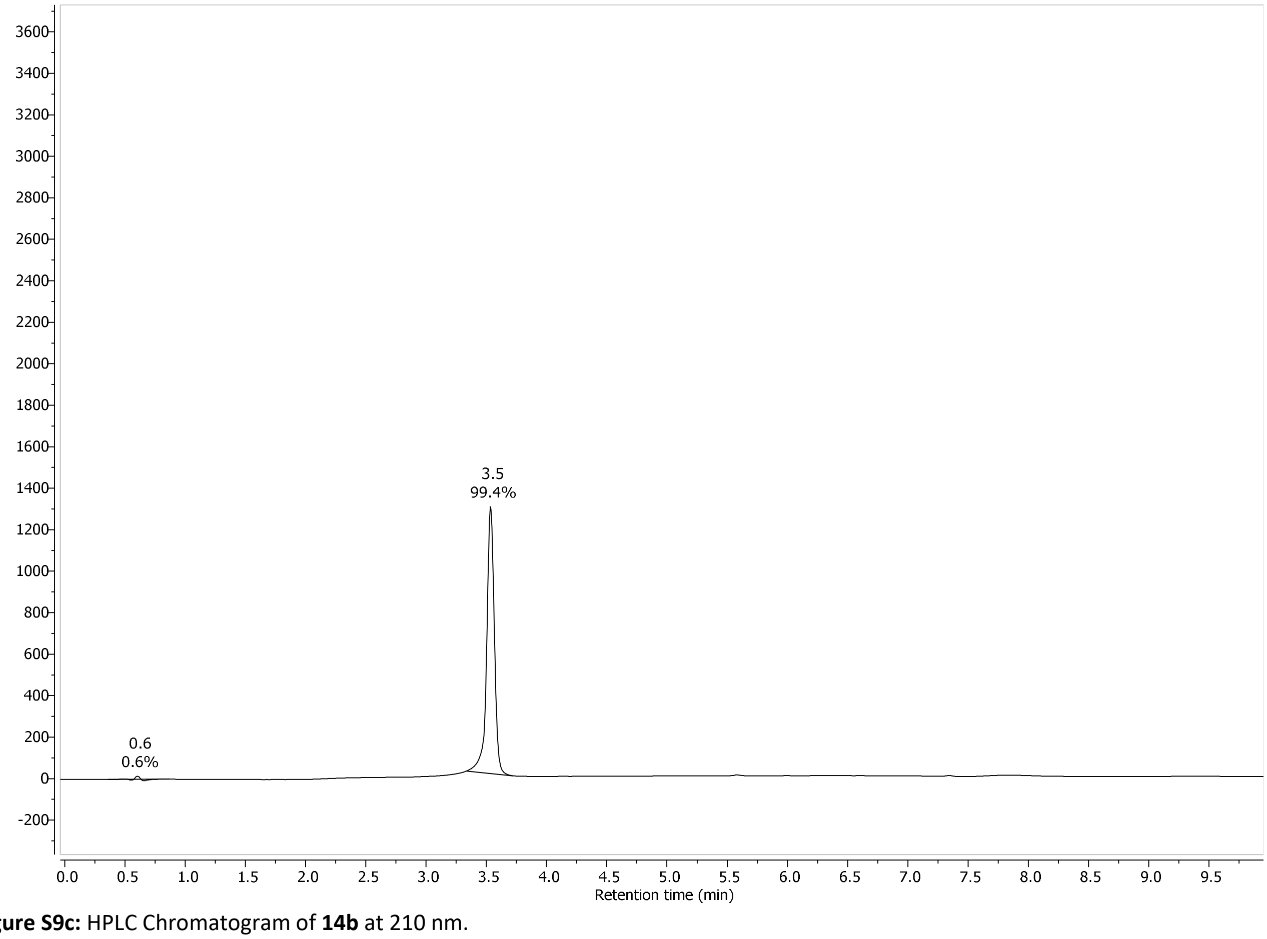

Figure S9c: HPLC Chromatogram of **14b** at 210 nm.

14c, (2*S*,3*S*)-2-(2-Acetamidoacetamido)-*N*-((*S*)-1-(((*S*)-5-guanidino-1-(6-chlorobenzo [d]thiazol-2-yl)-1-oxopentan-2-yl)amino)-1-oxopropan-2-yl)-3-hydroxybutanamide

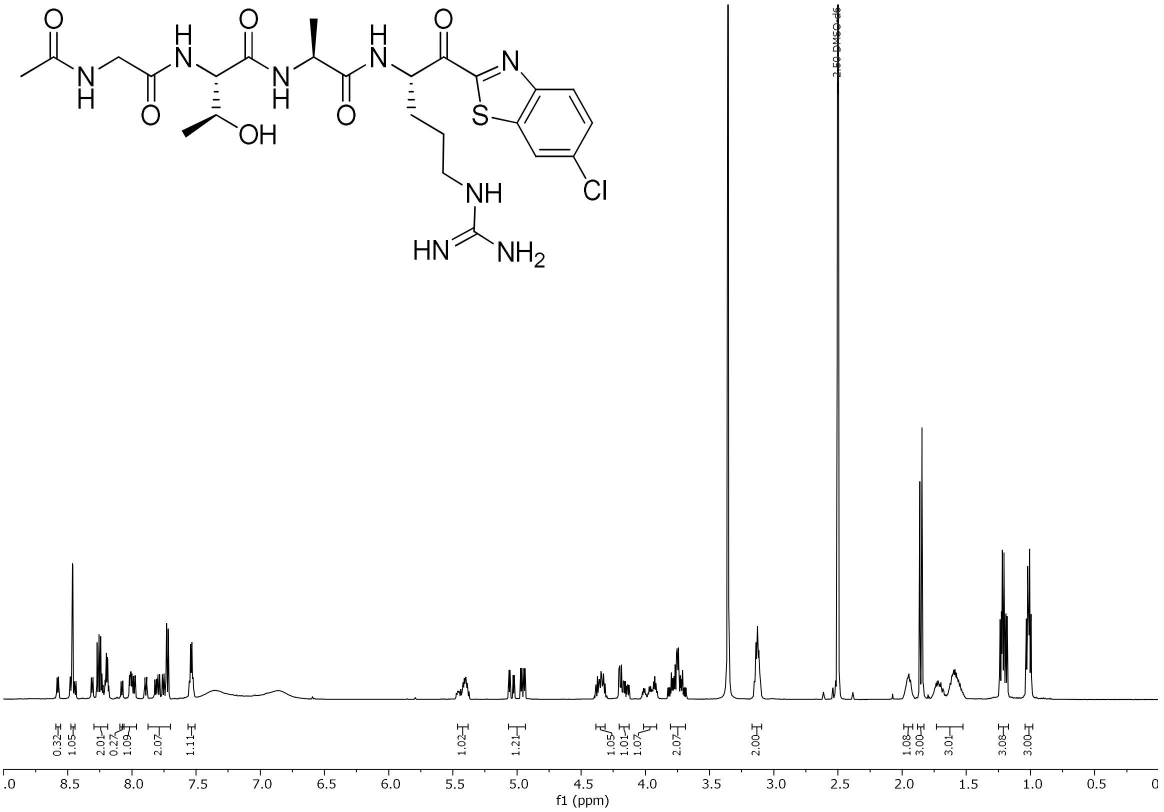

Figure S10a: ^{1}H NMR of **14c**.

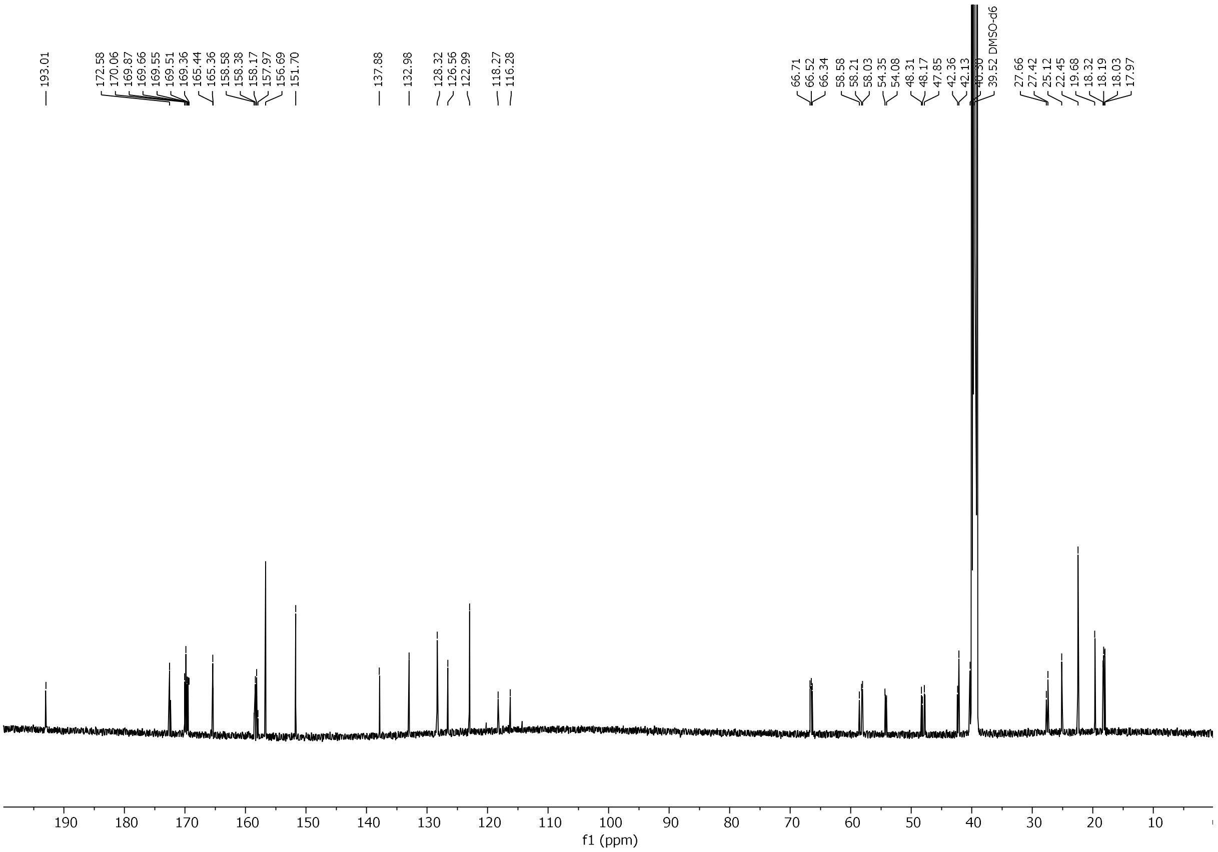

Figure S10b: ^{13}C NMR of **14c**.

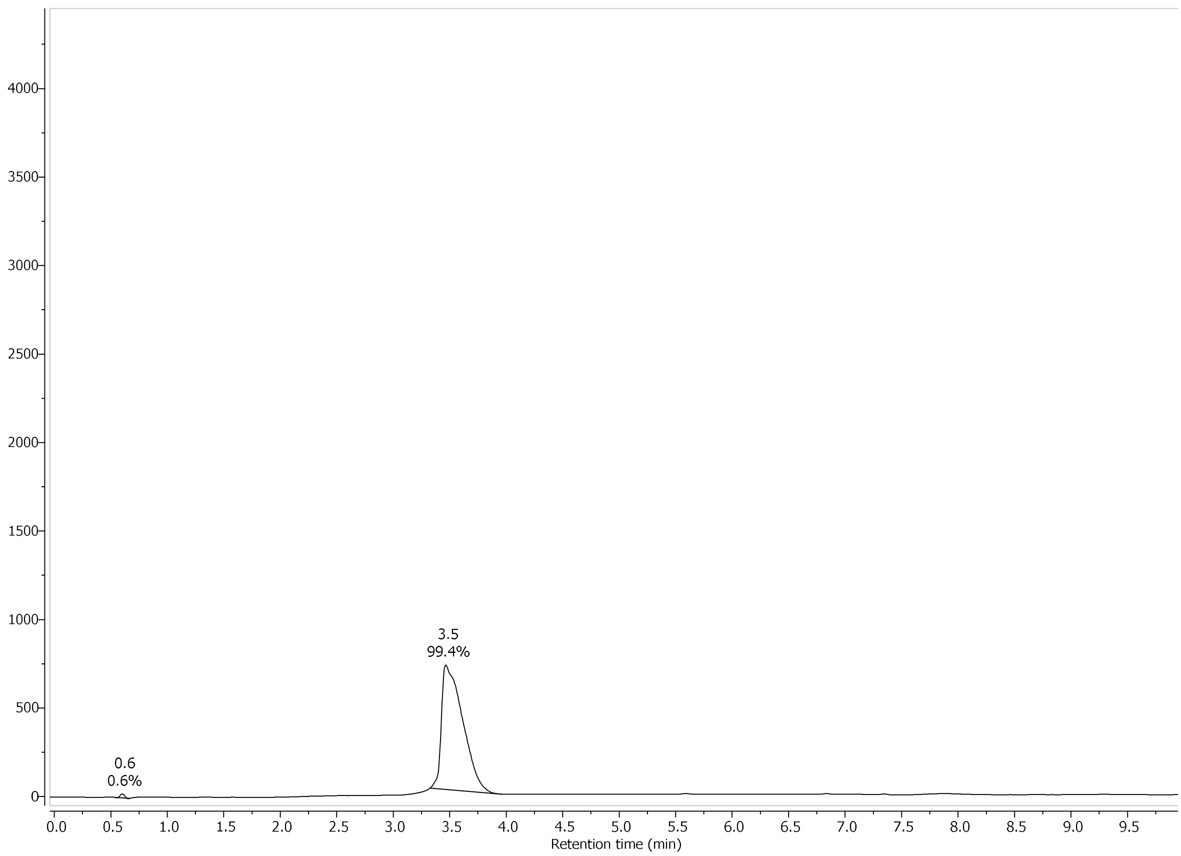

Figure S10c: HPLC Chromatogram of **14c** at 210 nm.

14d, (2*S*,3*S*)-2-(2-Acetamidoacetamido)-*N*-((*S*)-1-(((*S*)-5-guanidino-1-(6-bromobenzo [d]thiazol-2-yl)-1-oxopentan-2-yl)amino)-1-oxopropan-2-yl)-3-hydroxybutanamide

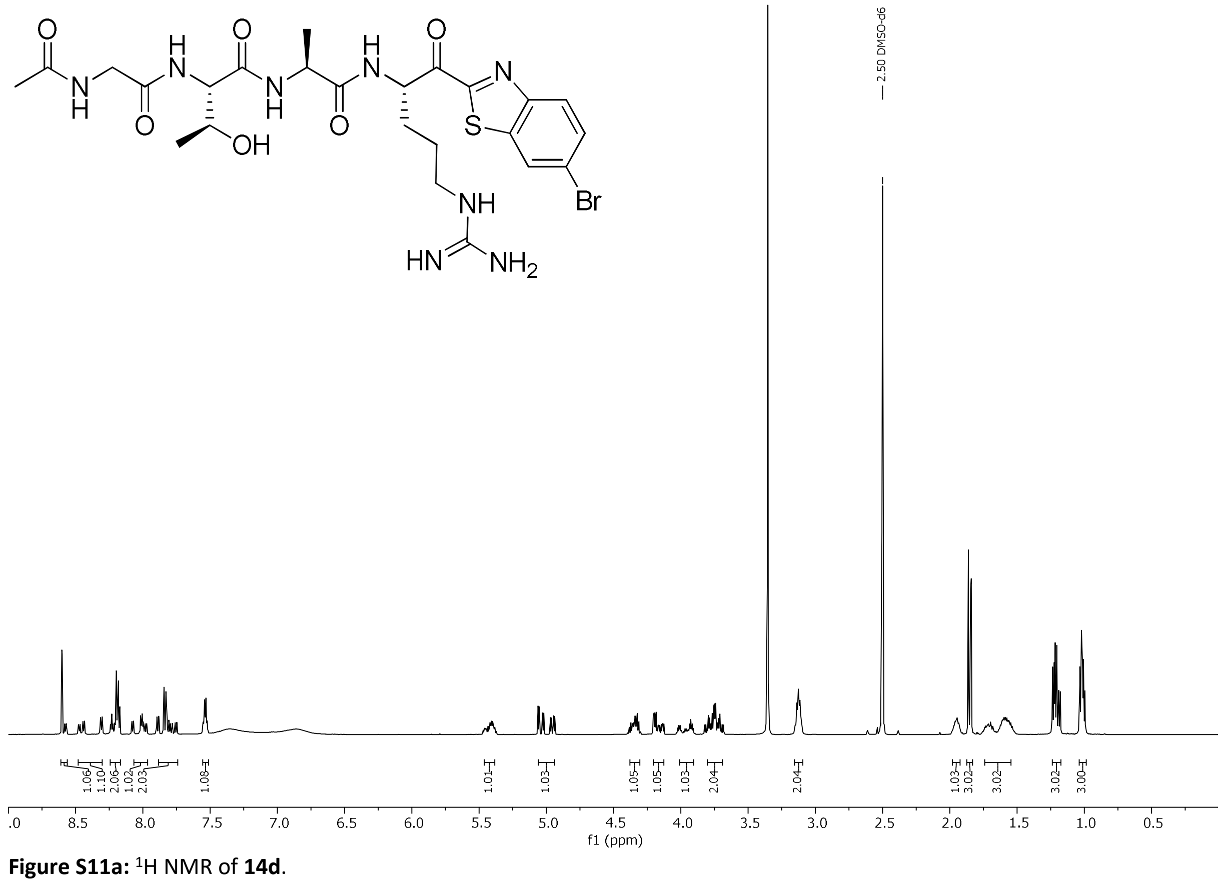

Figure S11a: ^{1}H NMR of **14d**.

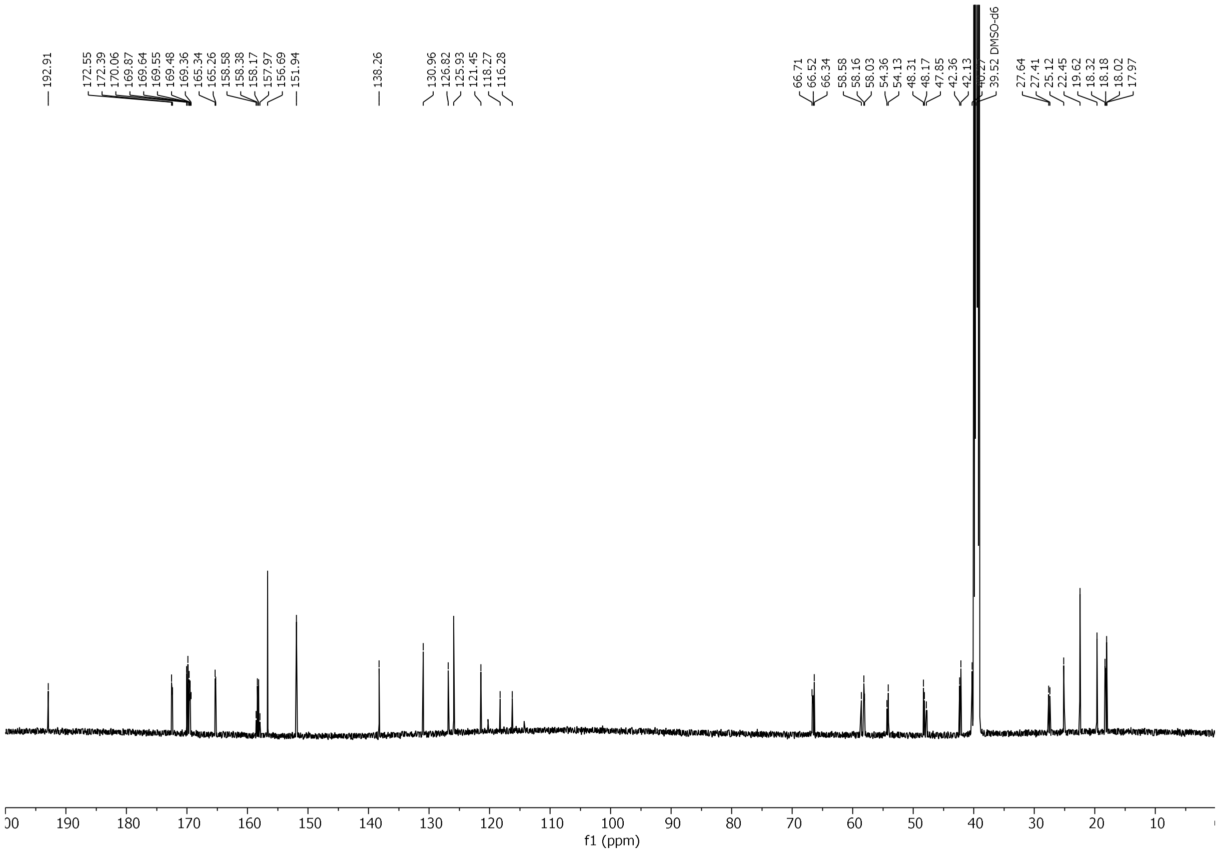

Figure S11b: ^{13}C NMR of **14d**.

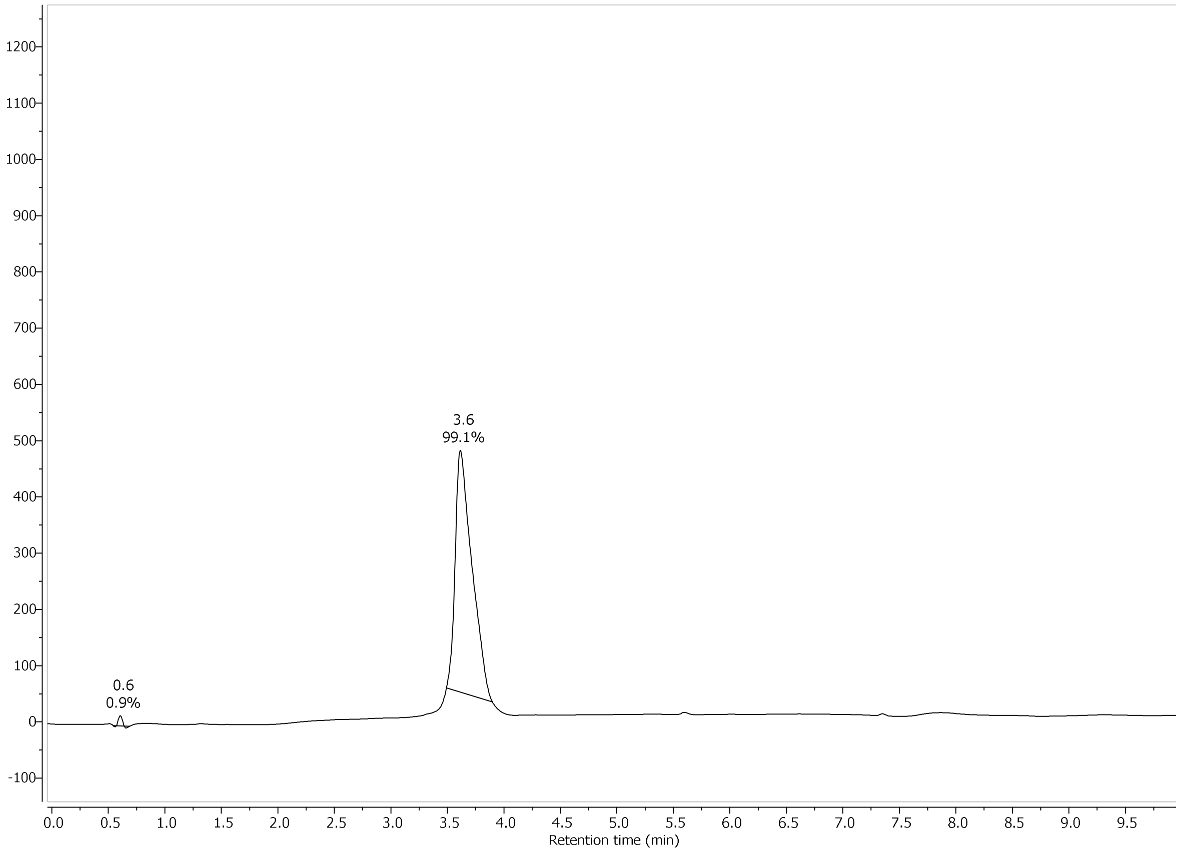

Figure S11c: HPLC Chromatogram of **14d** at 210 nm.

15, (2*S*,3*S*)-2-(2-Acetamidoacetamido)-*N*-((*S*)-1-(((*S*)-5-guanidino-1-oxo-1-(4,5,6,7-tetrahydrobenzo[d]thiazol-2-yl)pentan-2-yl)amino)-1-oxopropan-2-yl)-3-hydroxybutanamide

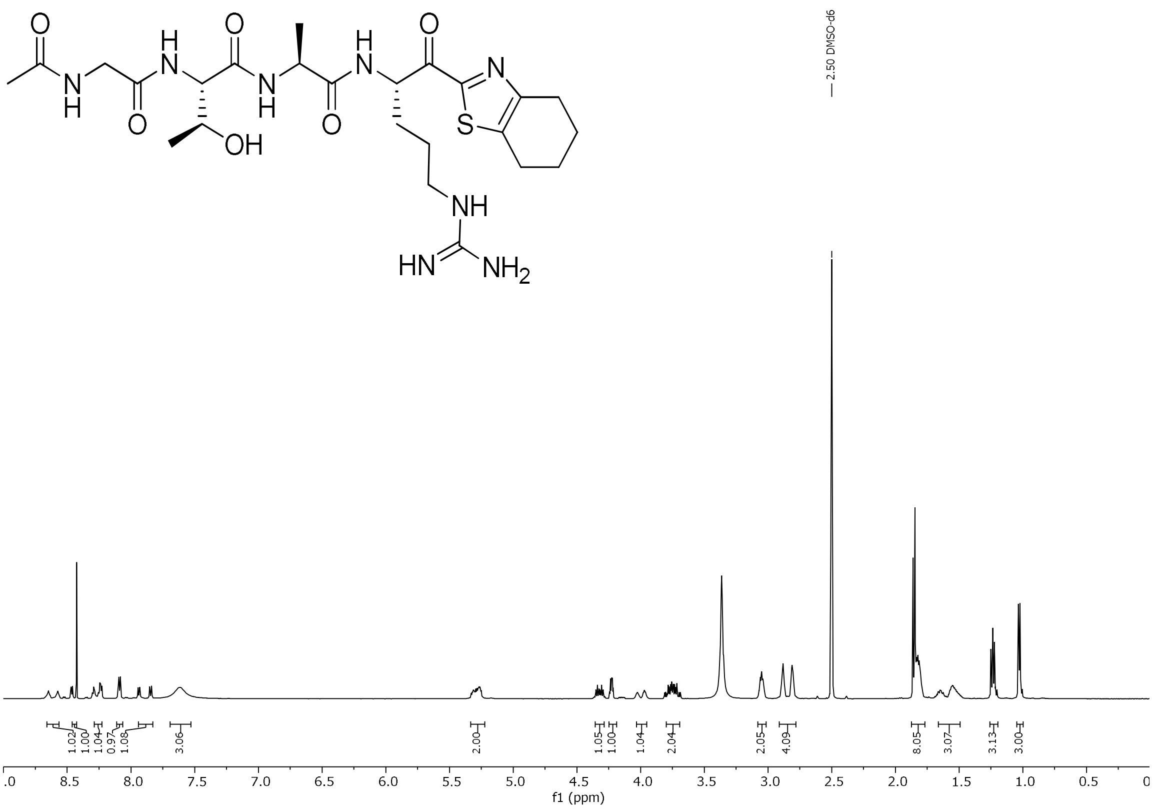

Figure S12a: ^{1}H NMR of **15**.

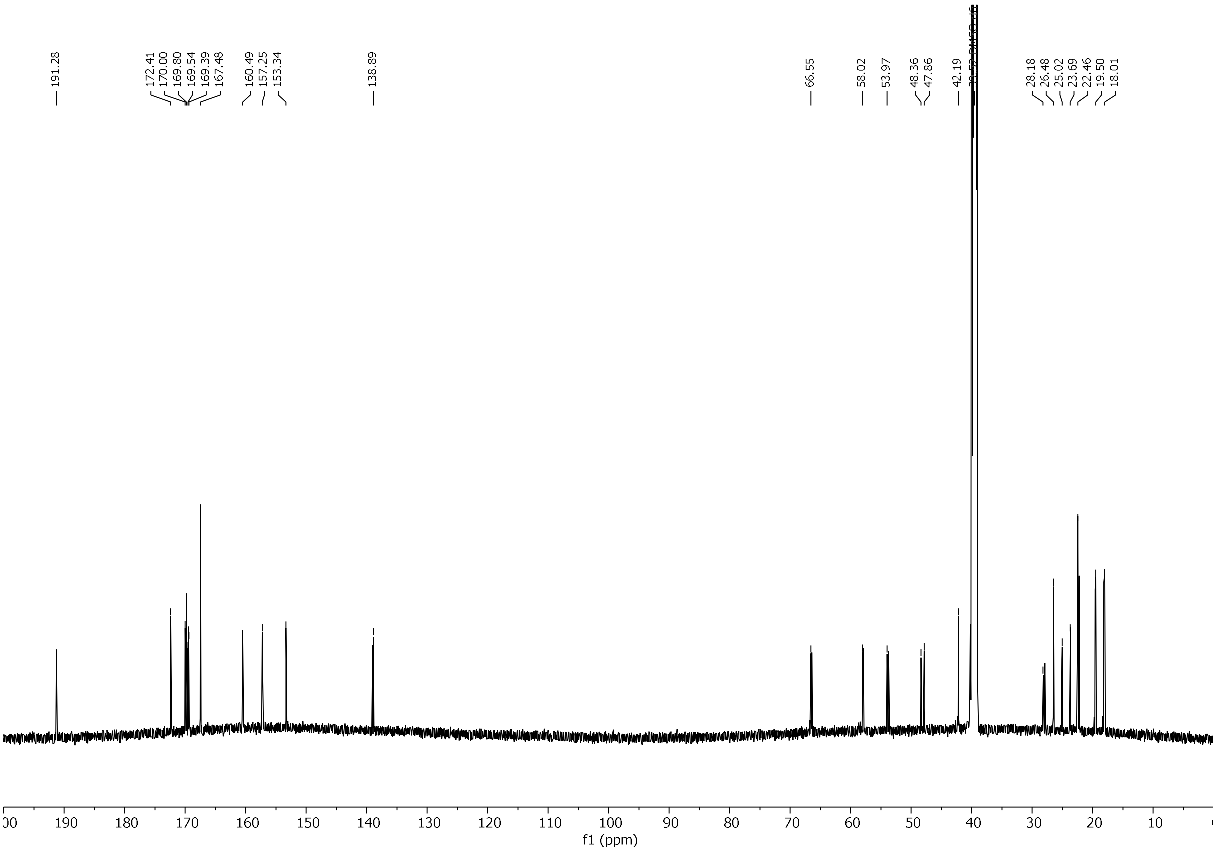

Figure S12b: ^{13}C NMR of **15**.

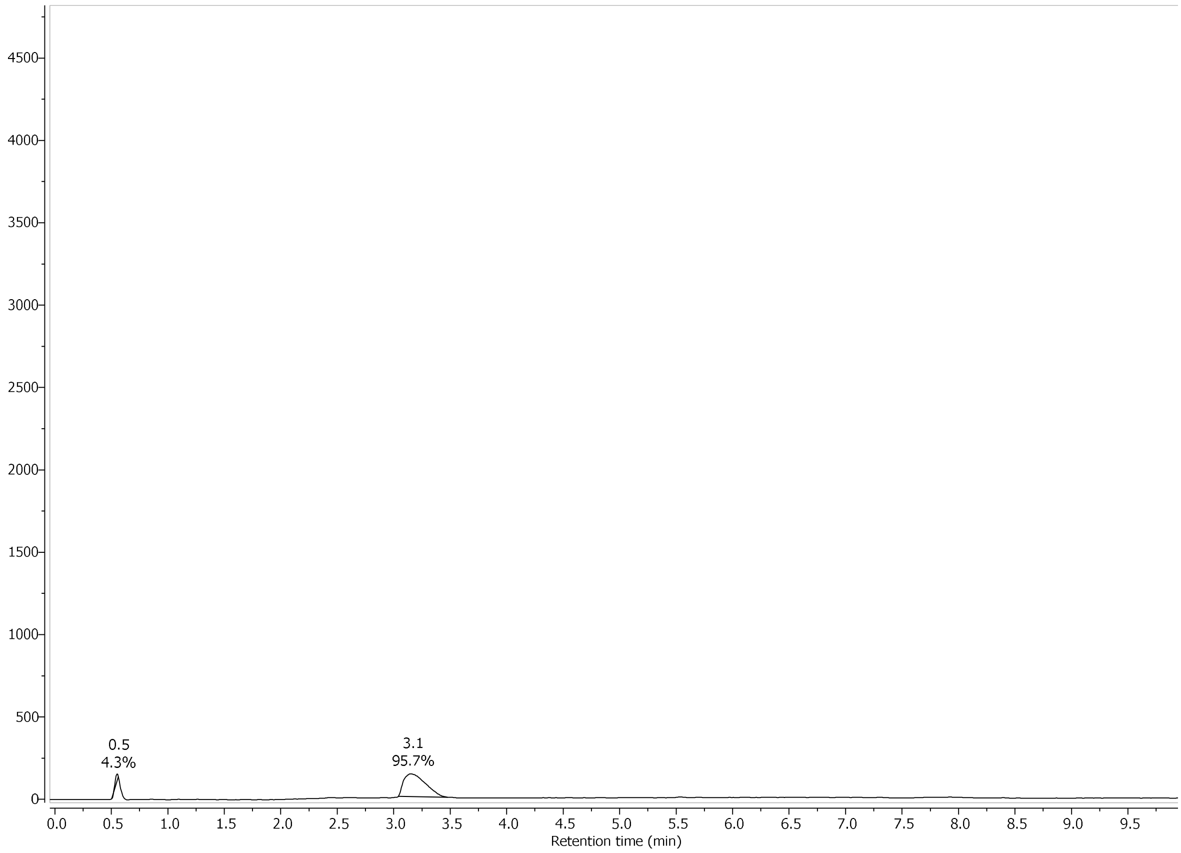

Figure S12c: HPLC Chromatogram of **15** at 210 nm.

20, (2*S*,3*S*)-2-(2-Acetamidoacetamido)-*N*-((*S*)-1-(((*S*)-1-(benzo[d]thiazol-2-yl)-6-guanidino-1-oxohexan-2-yl)amino)-1-oxopropan-2-yl)-3-hydroxybutanamide

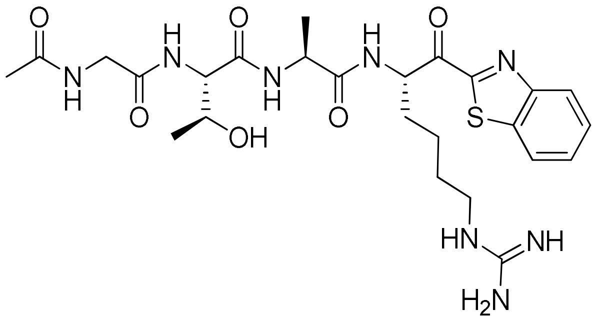

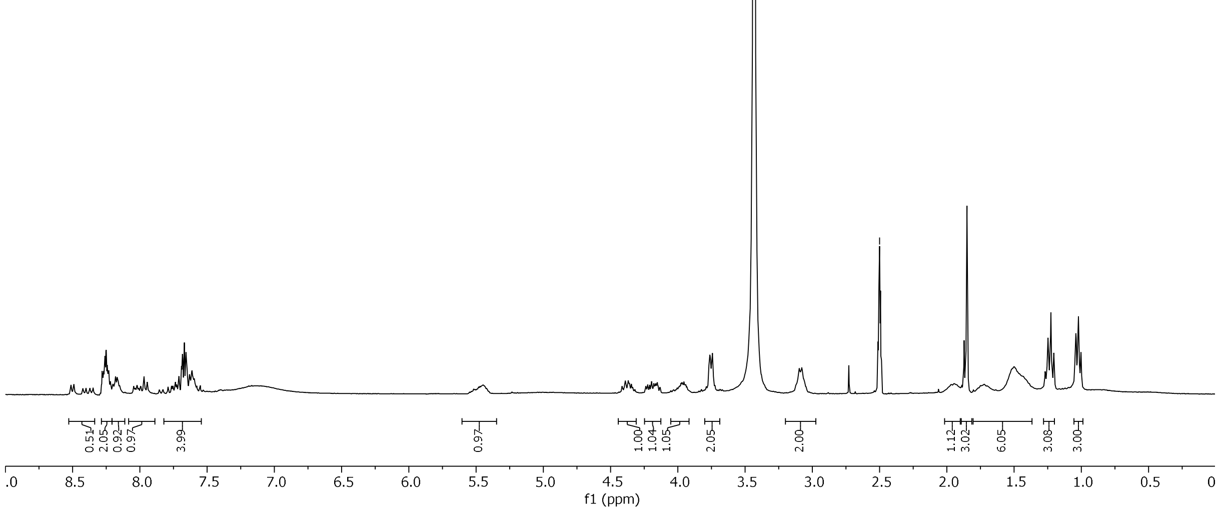

Figure S13a: ^{1}H NMR of **20**.

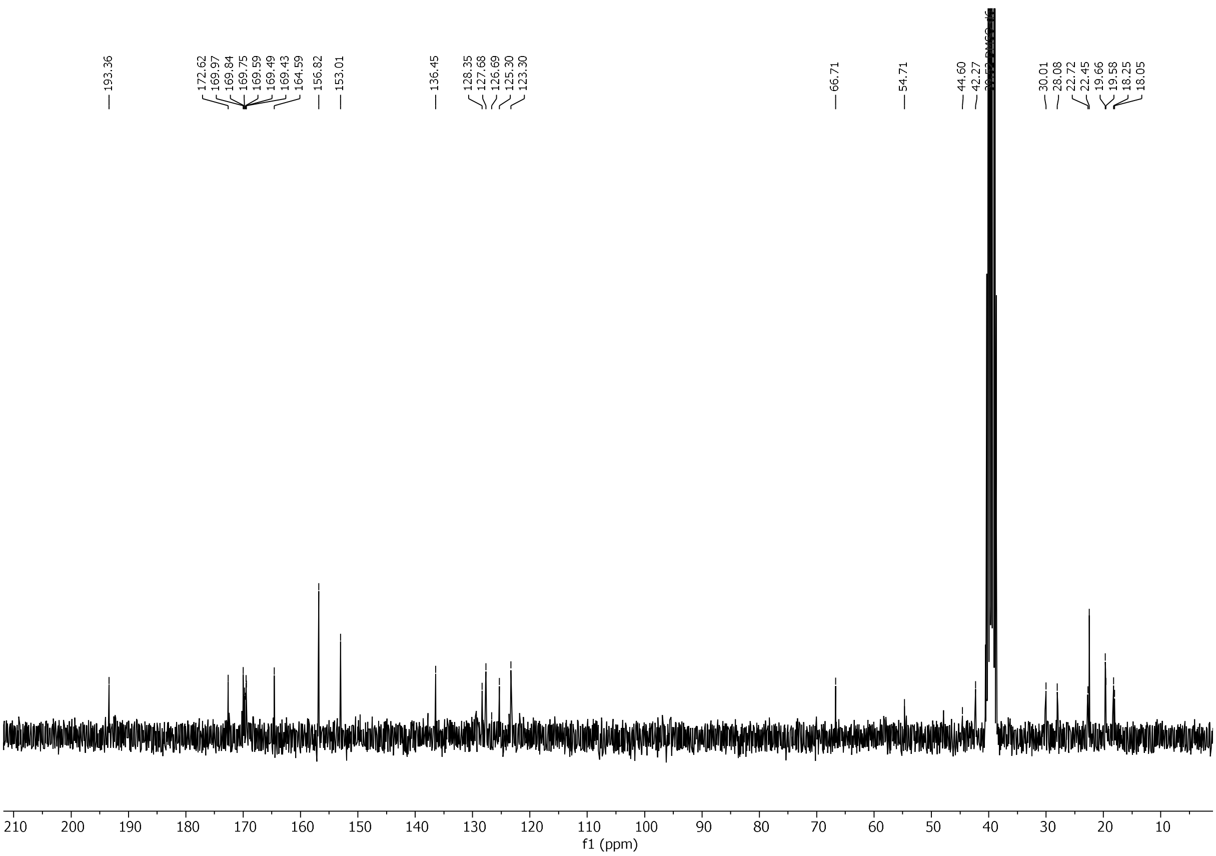

Figure S13b: ^{13}C NMR of **20**.

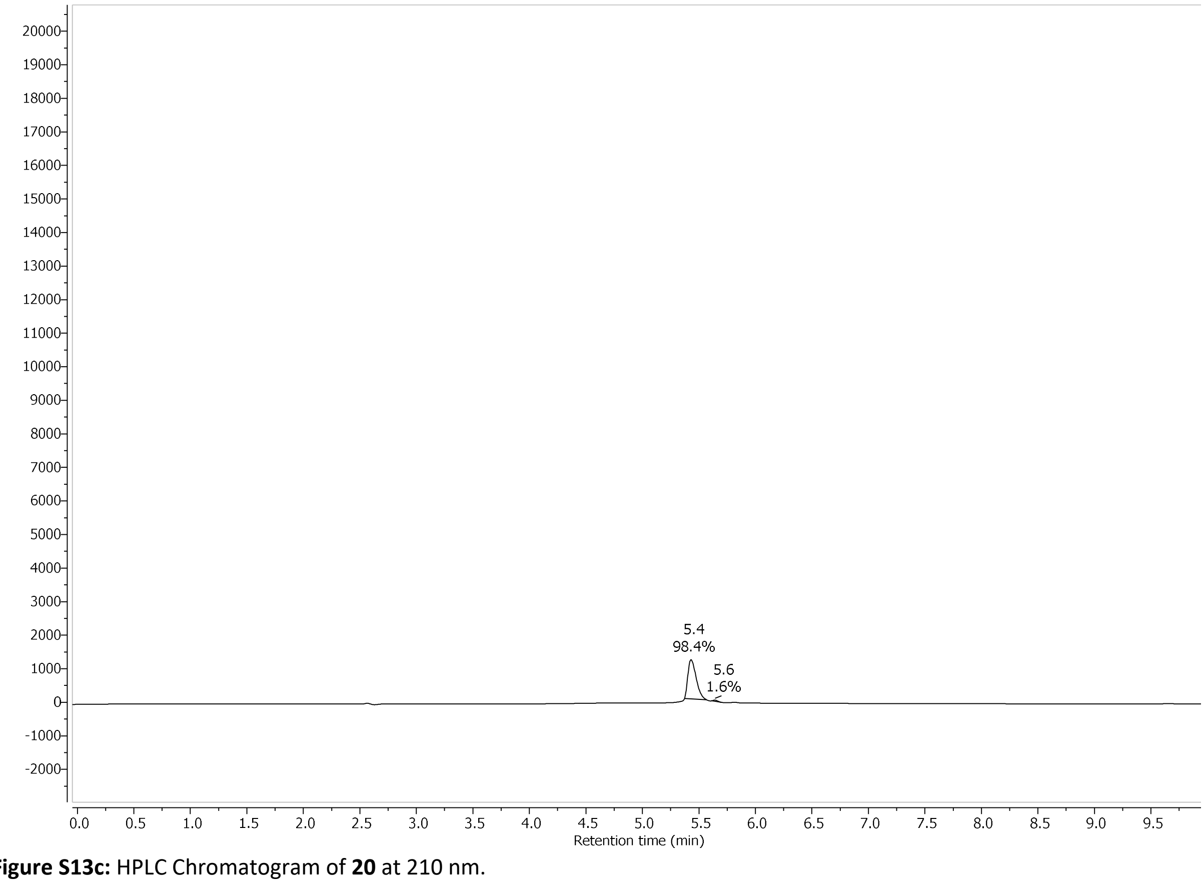

Figure S13c: HPLC Chromatogram of **20** at 210 nm.

29, (2*S*,3*S*)-2-(2-Acetamidoacetamido)-*N*-((*S*)-1-(((*S*)-1-(benzo[d]thiazol-2-yl)-3-(4-guanidinophenyl)-1-oxopropan-2-yl)amino)-1-oxopropan-2-yl)-3-hydroxybutanamide

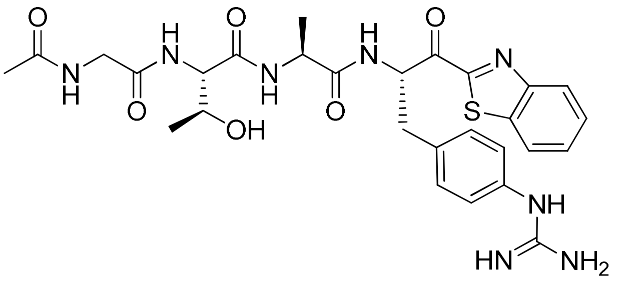

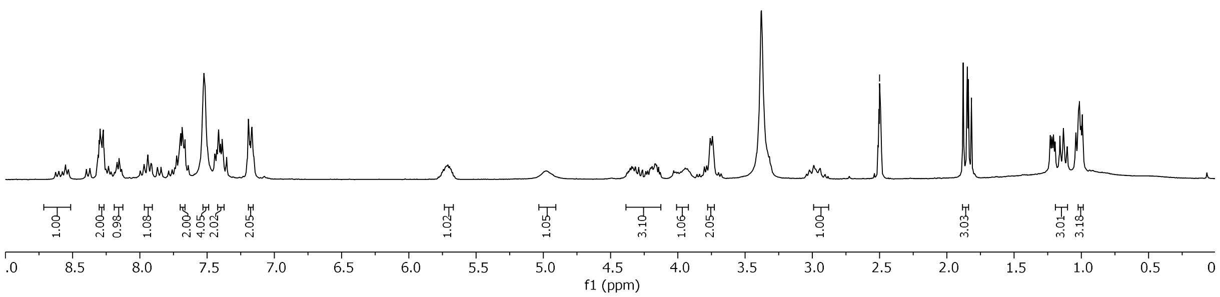

Figure S14a: ^{1}H NMR of **29**.

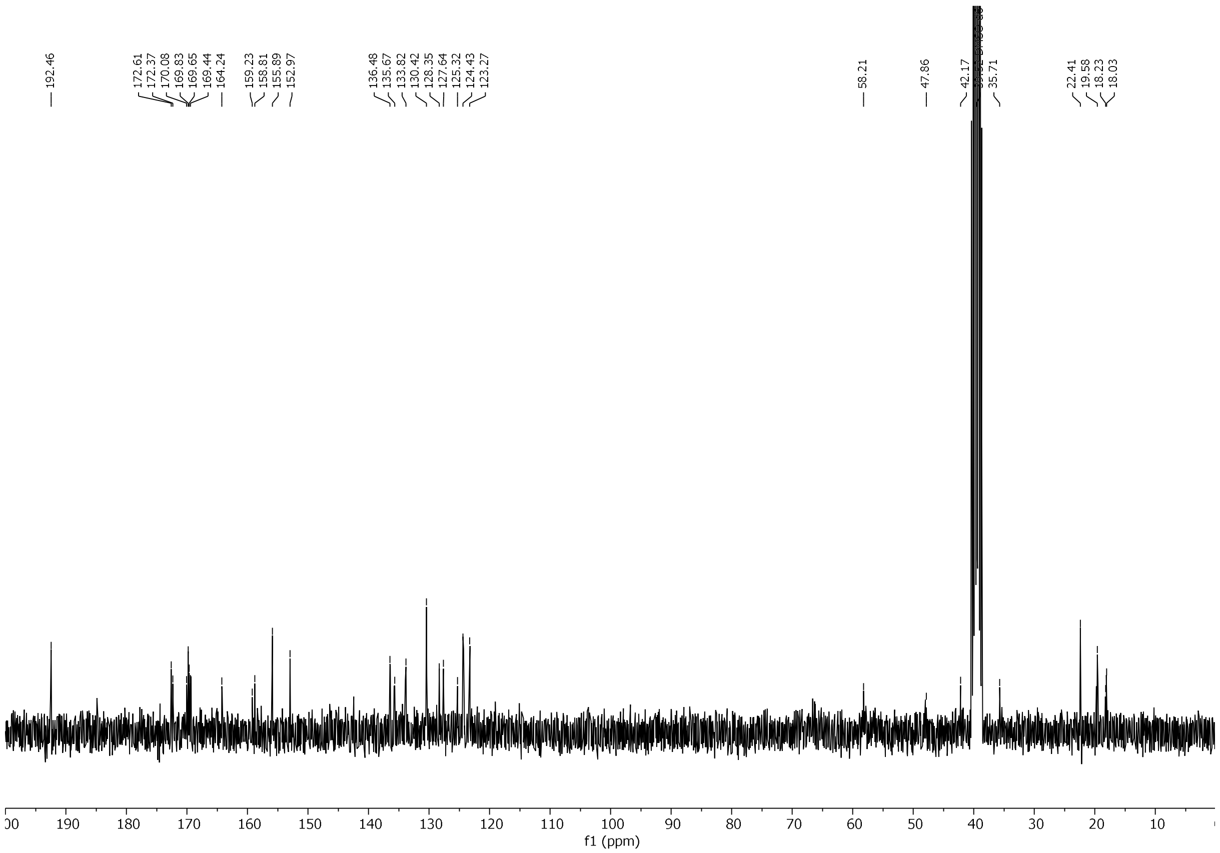

Figure S14b: ^{13}C NMR of **29**.

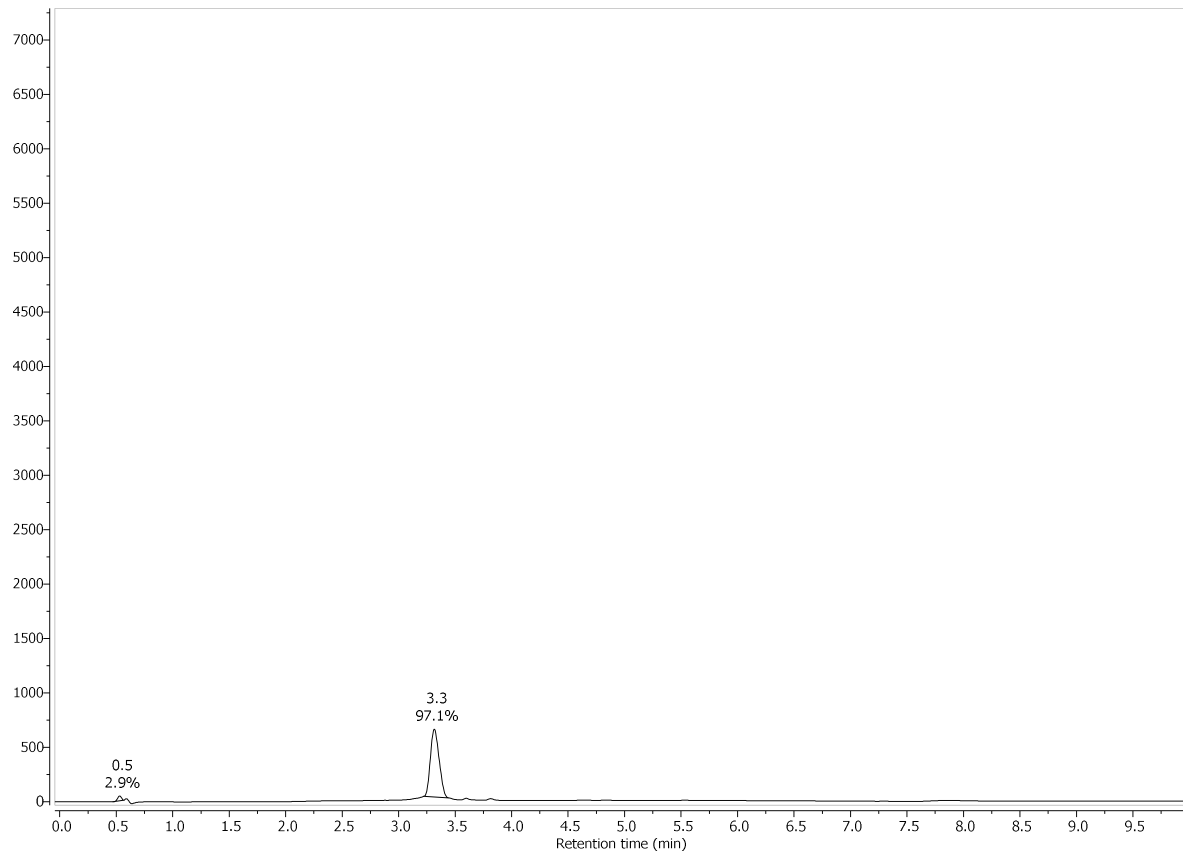

Figure S14c: HPLC Chromatogram of **29** at 210 nm.

45, (2*S*,3*S*)-2-(2-Acetamidoacetamido)-*N*-((*S*)-1-(((*S*)-1-(benzo[d]thiazol-2-yl)-3-(3-guanidinophenyl)-1-oxopropan-2-yl)amino)-1-oxopropan-2-yl)-3-hydroxybutanamide

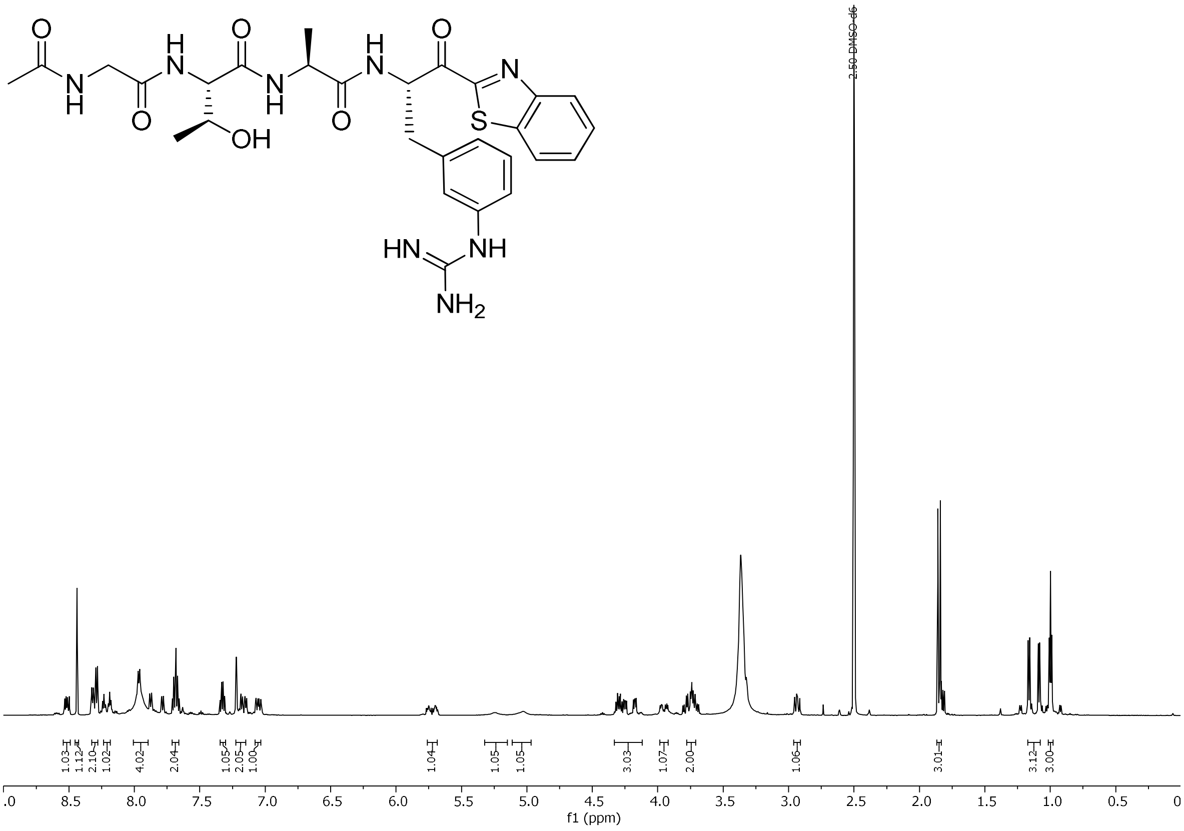

Figure S15a: ^{1}H NMR of **45**.

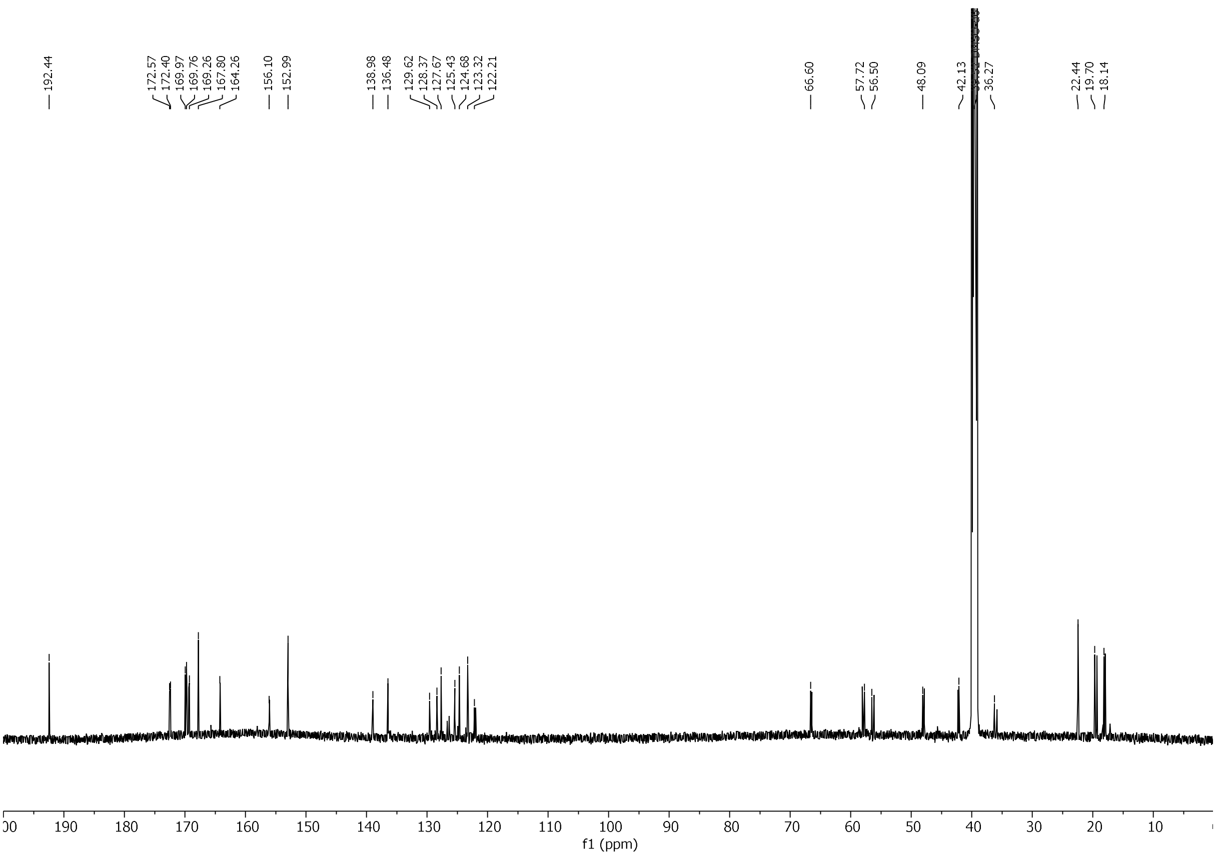

Figure S15b: ^{13}C NMR of **45**.

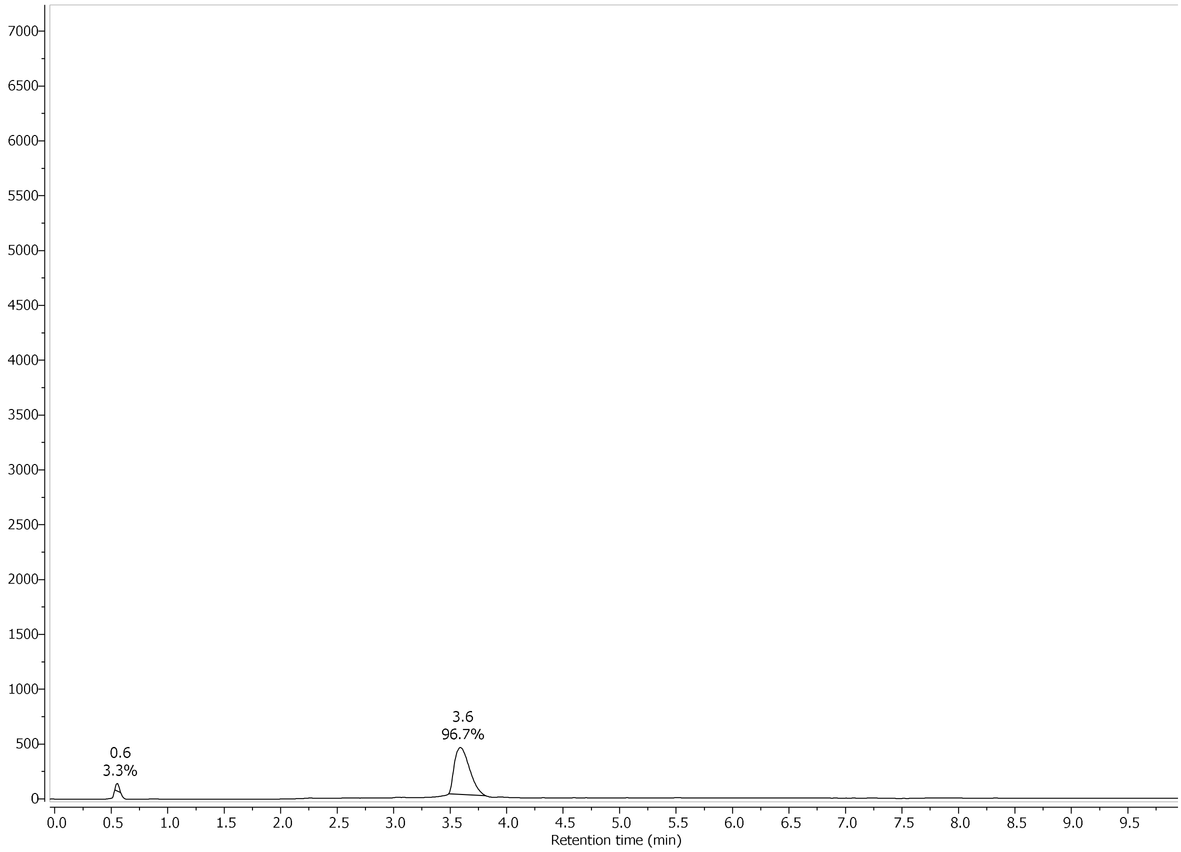

Figure 15c: HPLC Chromatogram of **45** at 210 nm.

38, (2*S*,3*S*)-2-(2-Acetamidoacetamido)-*N*-((*S*)-1-(((*S*)-1-(benzo[d]thiazol-2-yl)-3-(4-guanidinocyclohexyl)-1-oxopropan-2-yl)amino)-1-oxopropan-2-yl)-3-hydroxybutanamide

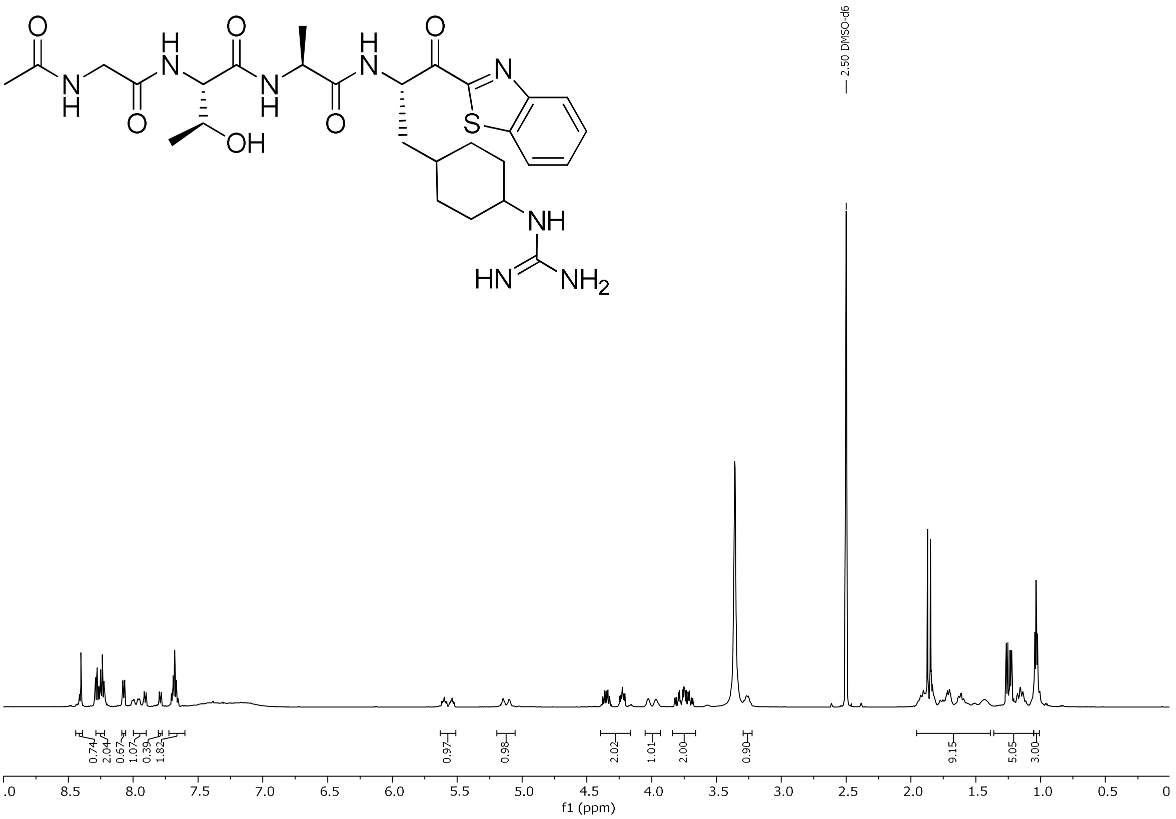

Figure S16a: ^{1}H NMR of **38**.

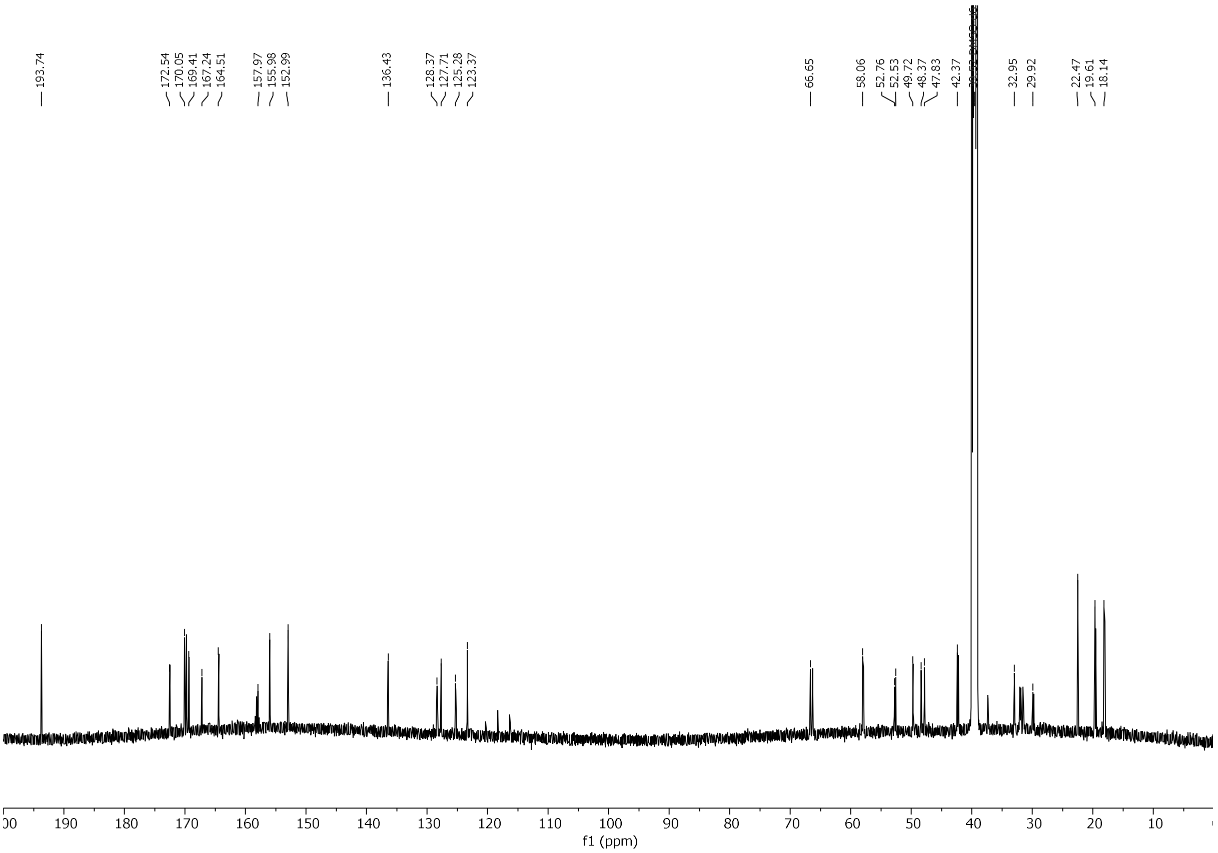

Figure S16b: ^{13}C NMR of **38**.

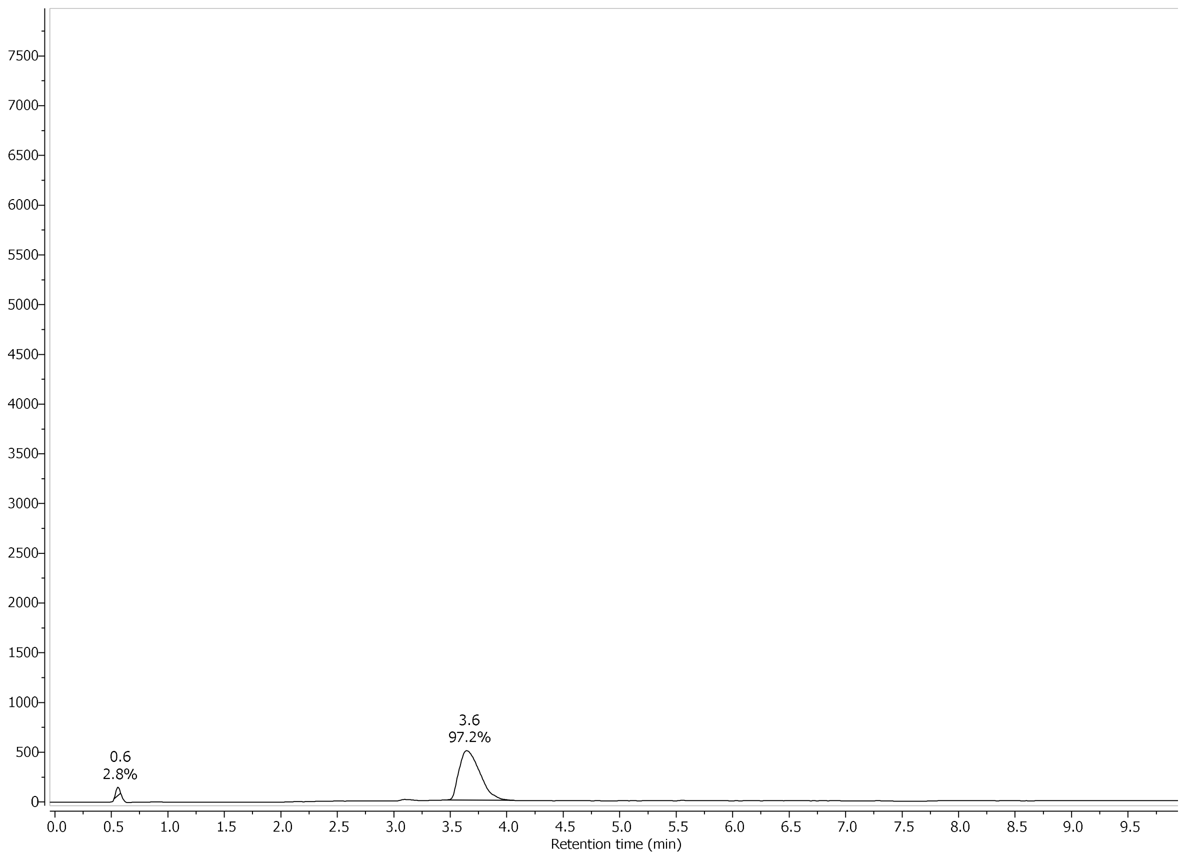

Figure S16c: HPLC Chromatogram of **38** at 210 nm.

30, (*S*)-1-((*S*)-2-Acetamido-4-amino-4-oxobutanoyl)-*N*-((*S*)-1-(benzo[d]thiazol-2-yl)-3-(4-guanidinophenyl)-1-oxopropan-2-yl)pyrrolidine-2-carboxamide

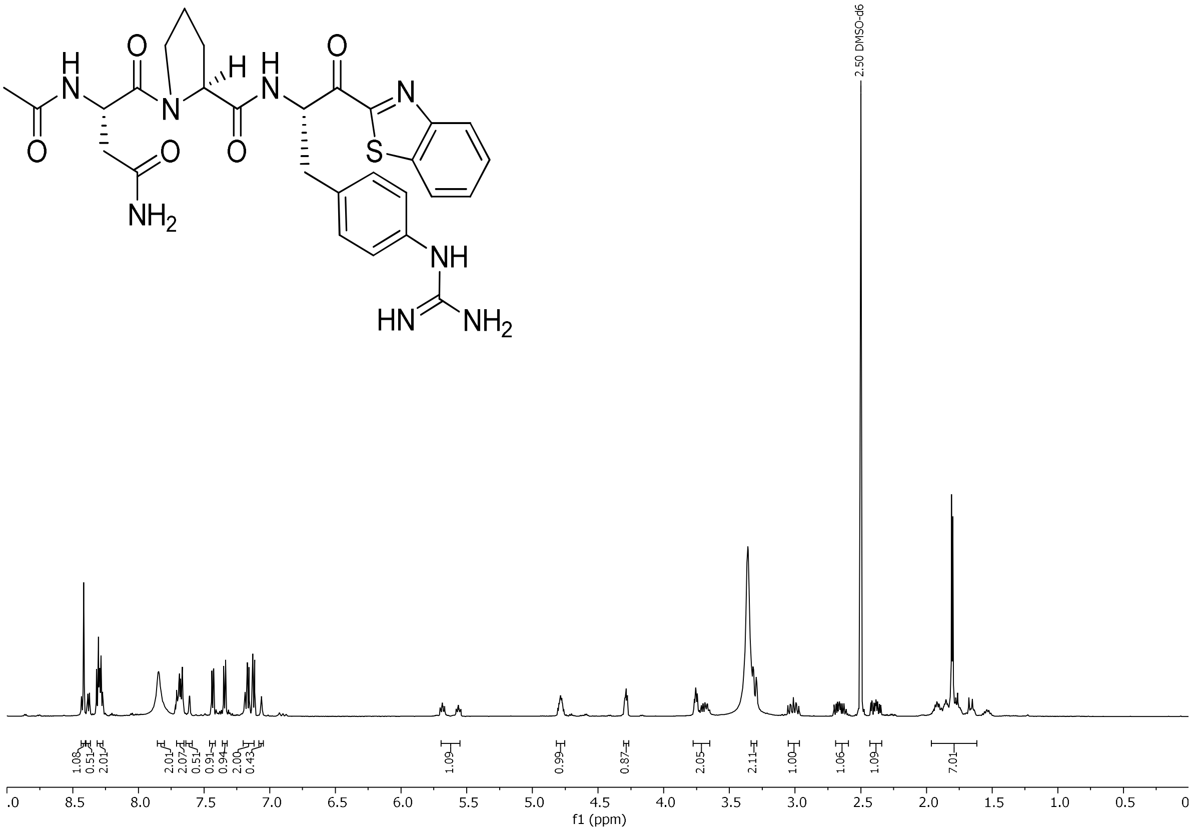

Figure S17a: ^{1}H NMR of **30**.

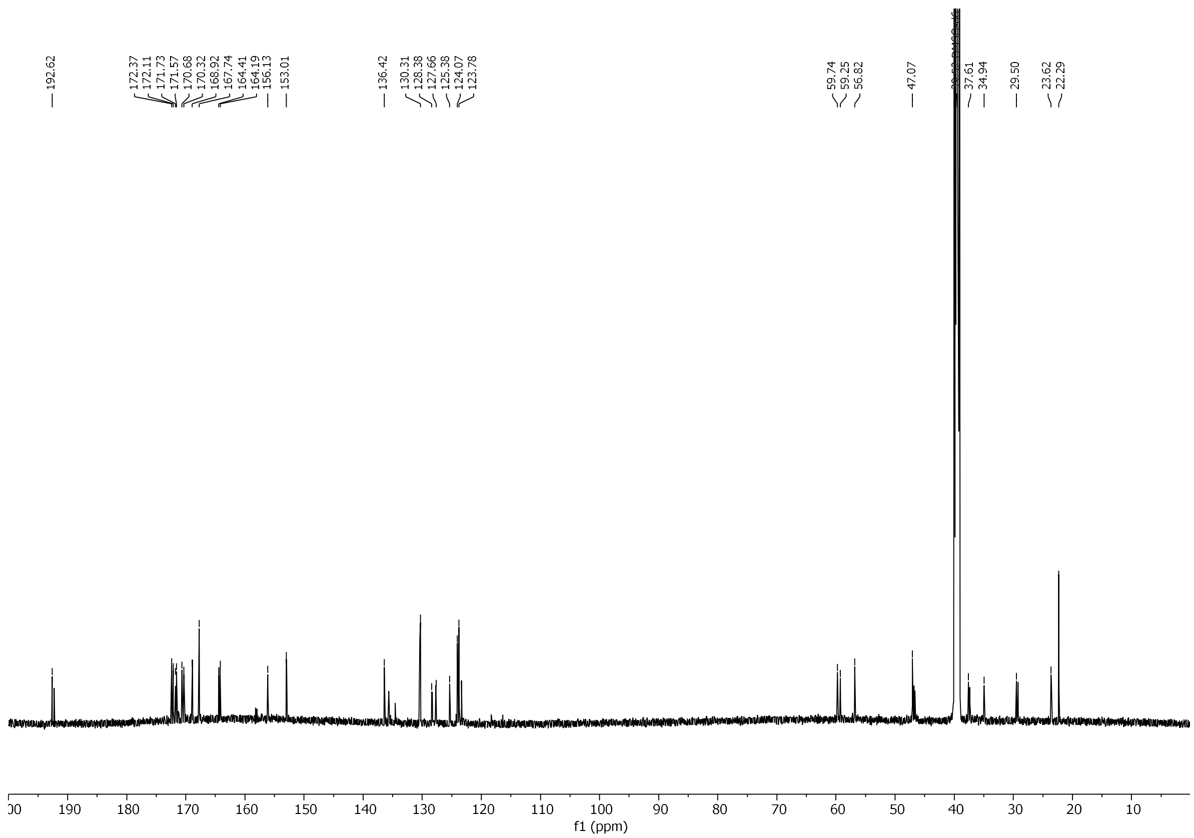

Figure S17b: ^{13}C NMR of **30**.

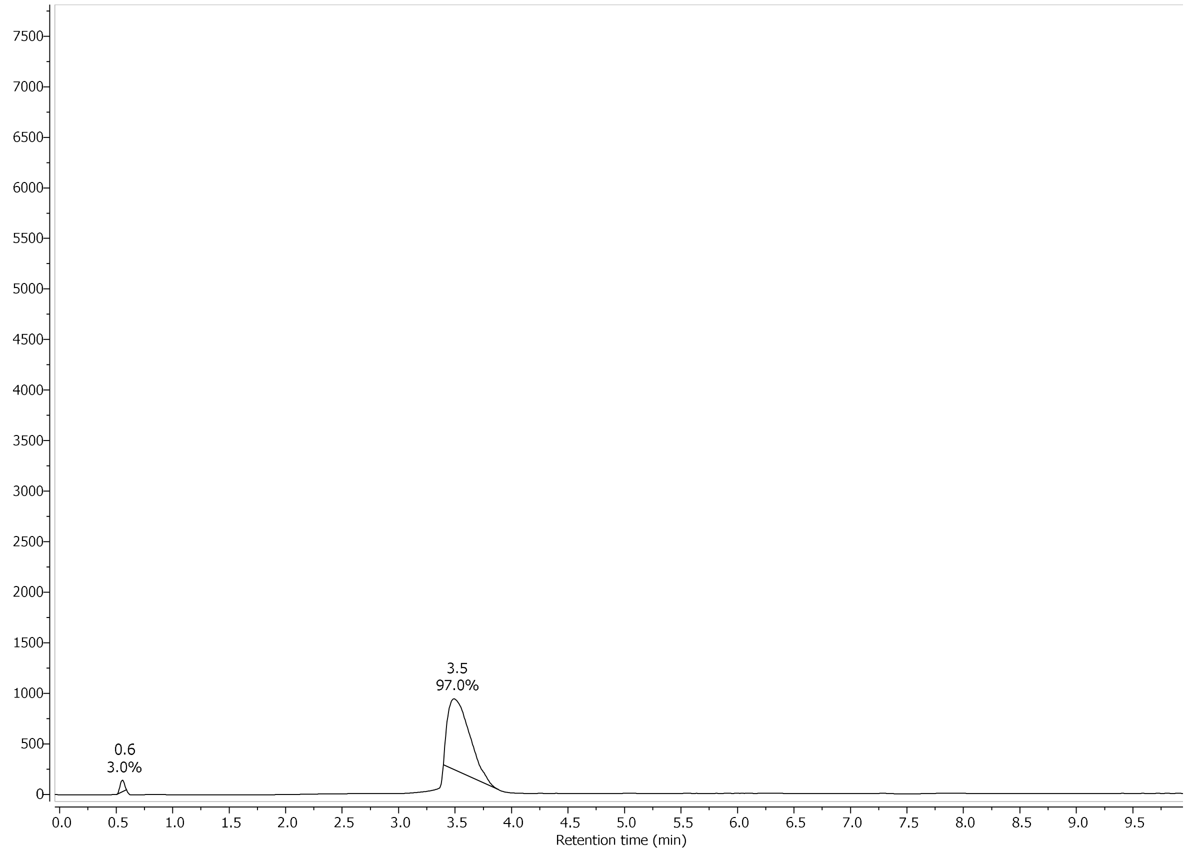

Figure S17c: HPLC Chromatogram of **30** at 210 nm.

39, (*S*)-1-((*S*)-2-Acetamido-4-amino-4-oxobutanoyl)-*N*-((*S*)-1-(benzo[d]thiazol-2-yl)-3-(4-guanidinocyclohexyl)-1-oxopropan-2-yl)pyrrolidine-2-carboxamide

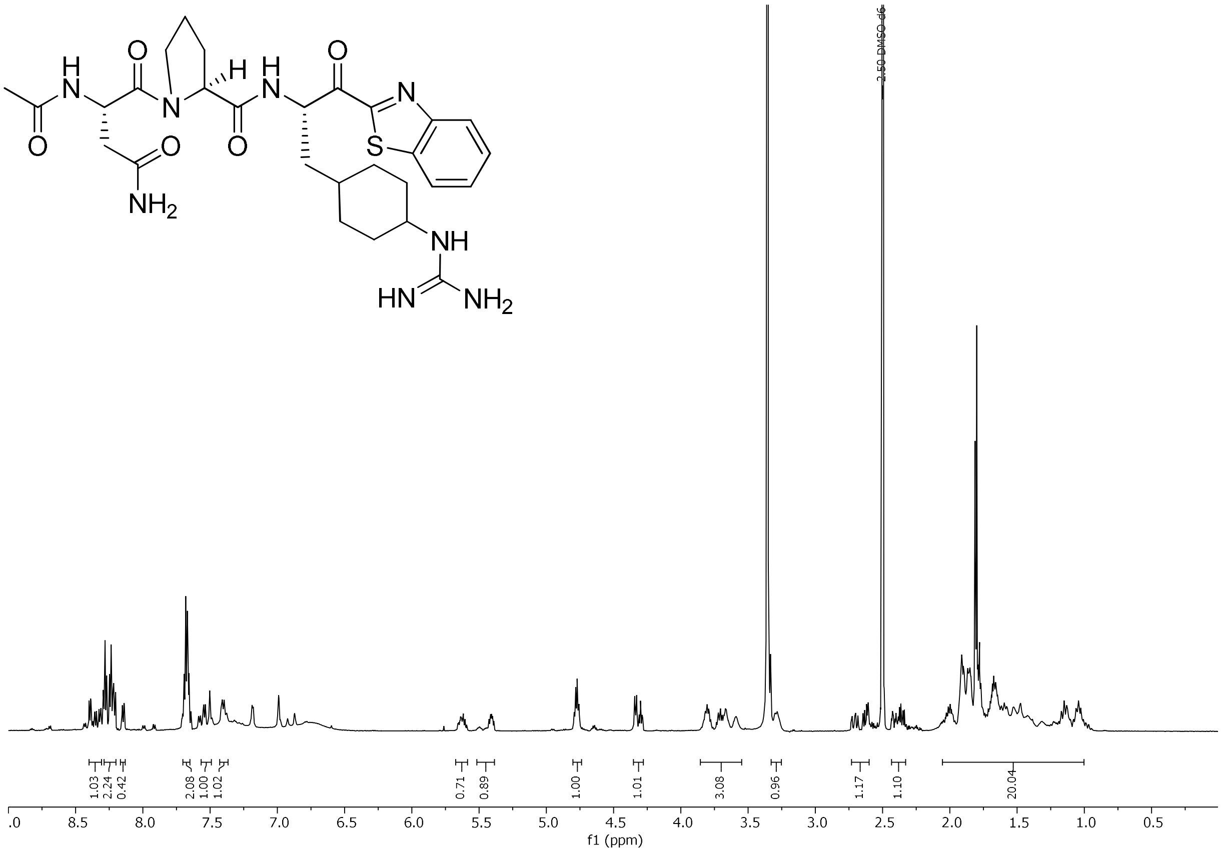

Figure S18a: ^{1}H NMR of **39**.

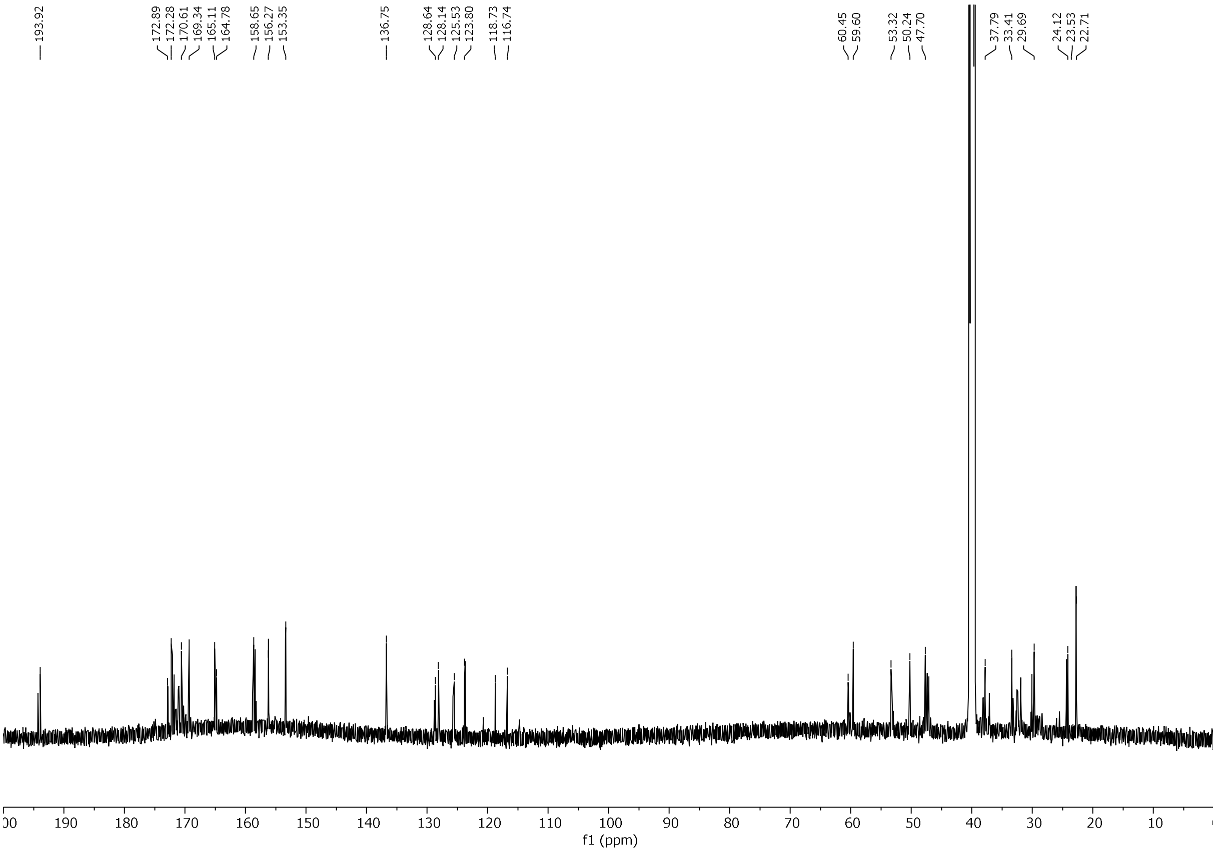

Figure S18b: ^{13}C NMR of **39**.

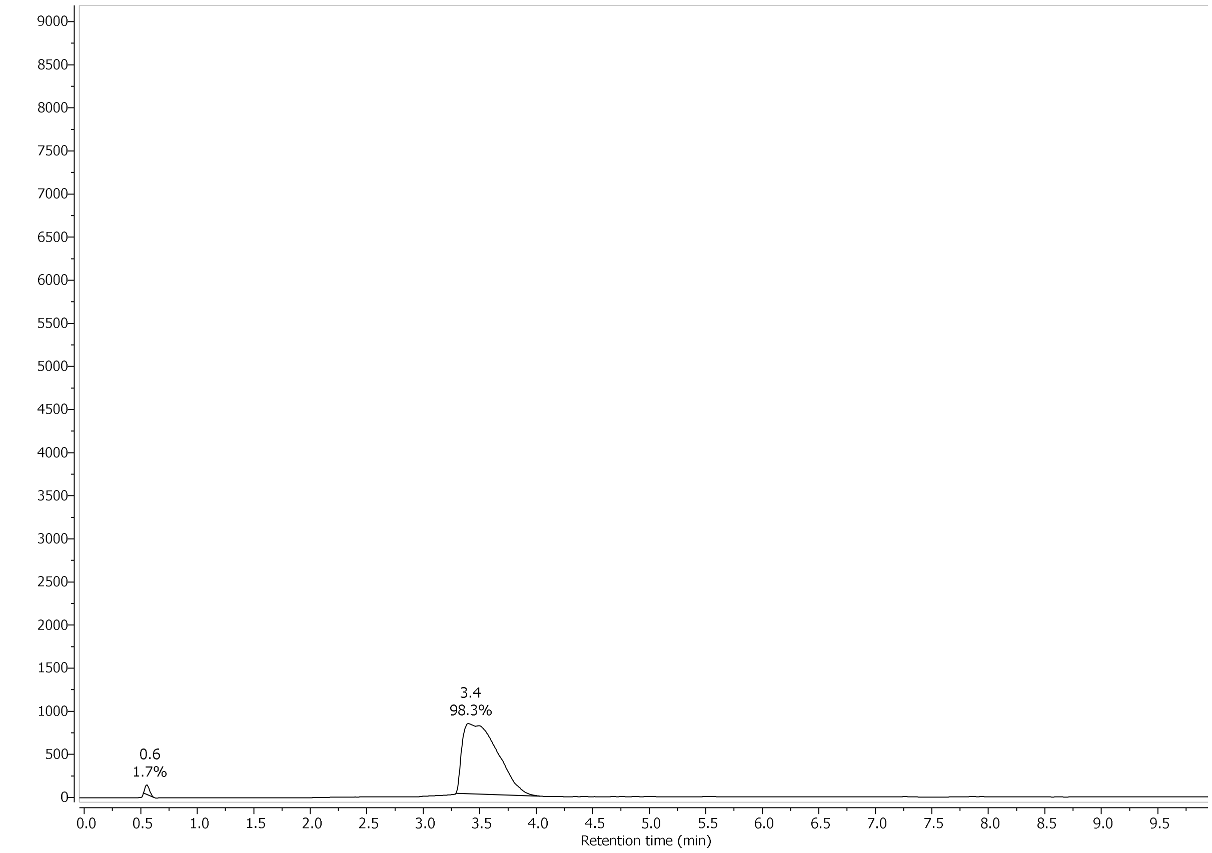

Figure S18c: HPLC Chromatogram of **39** at 210 nm.

31, (*S*)-2-Acetamido-*N*-((*S*)-1-(((*S*)-1-(benzo[d]thiazol-2-yl)-3-(4-guanidinophenyl)-1-oxopropan-2-yl)amino)-1-oxo-3-phenylpropan-2-yl)succinamide

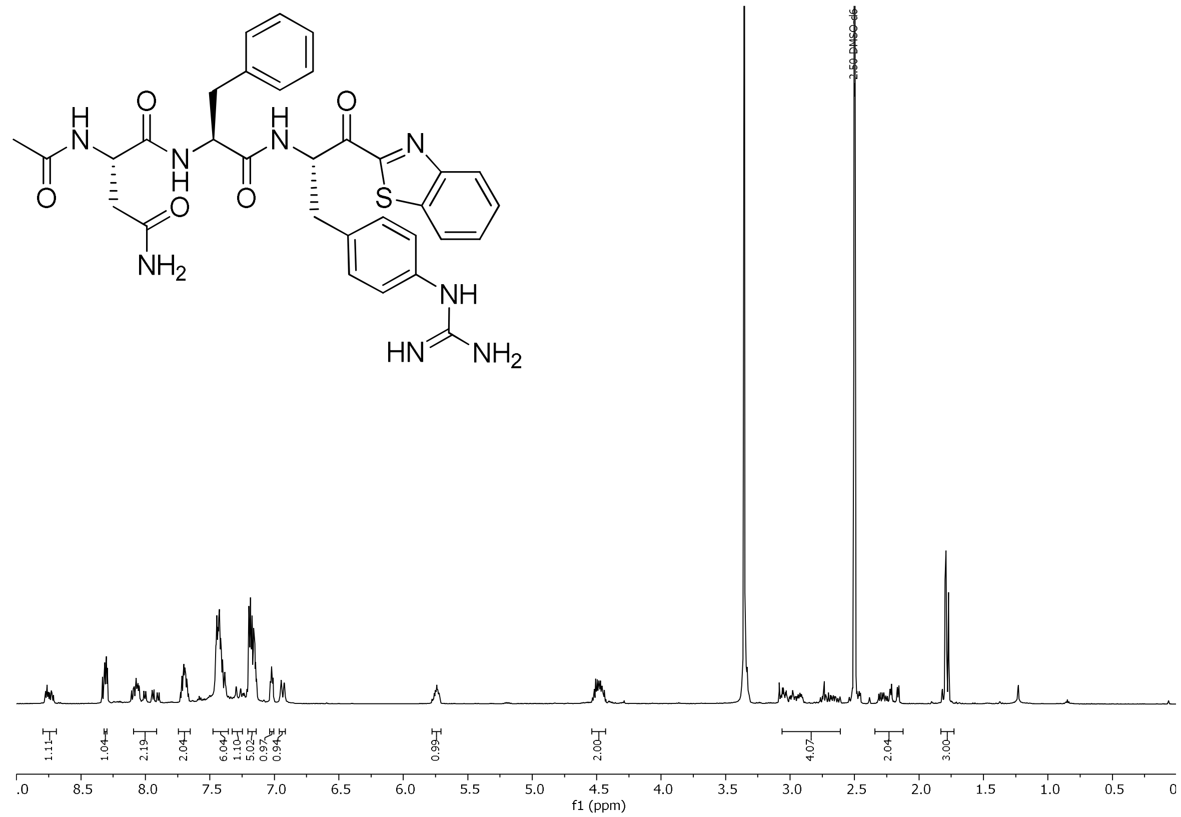

Figure S19a: ^{1}H NMR of **31**.

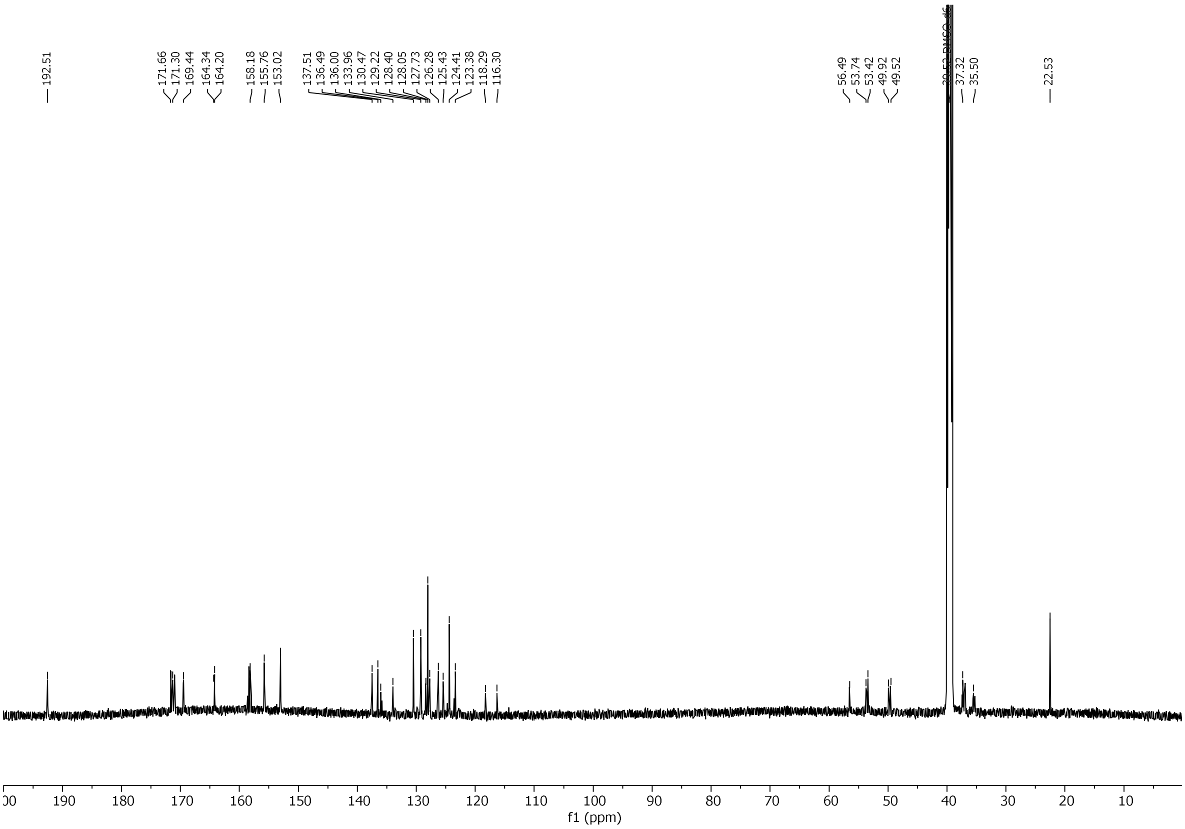

Figure S19b: ^{13}C NMR of **31**.

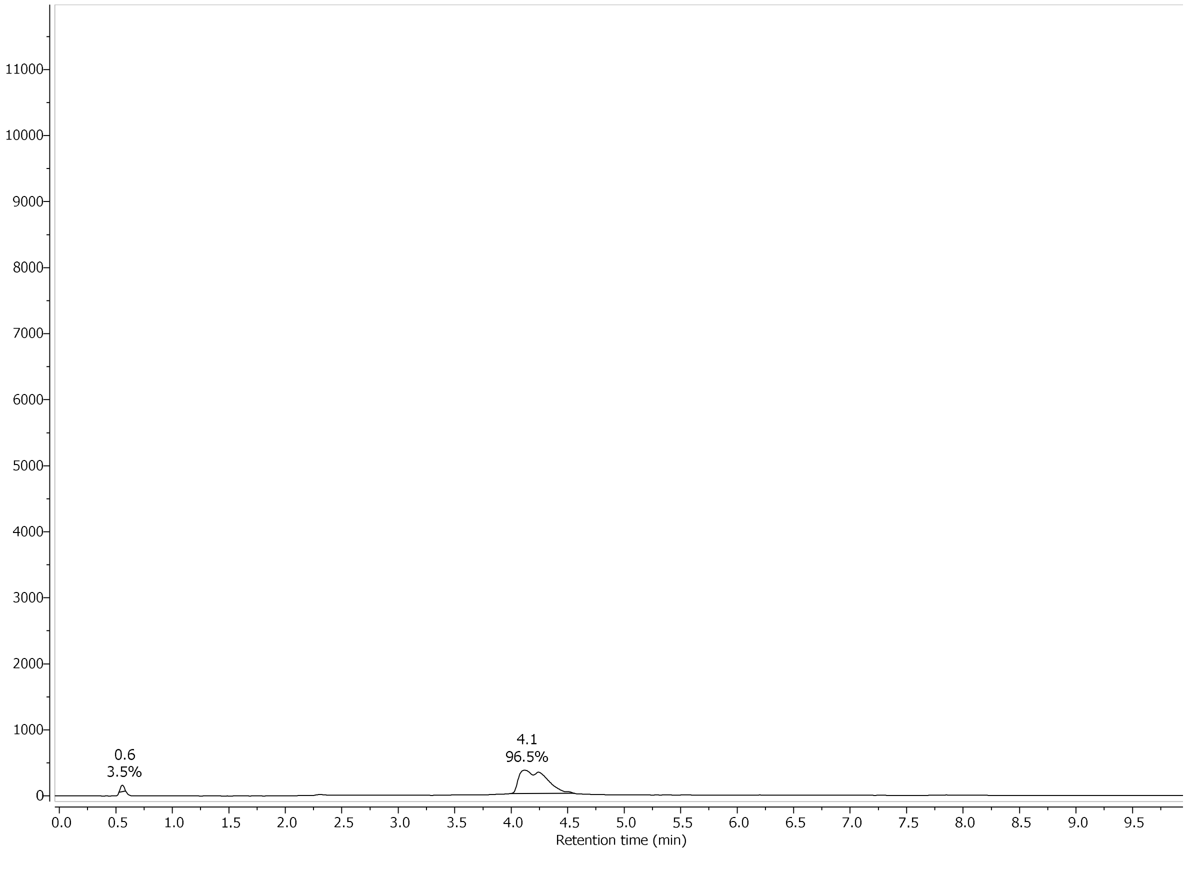

Figure S19c: HPLC Chromatogram of **31** at 210 nm.

32, (*S*)-2-Acetamido-*N*-((*S*)-1-(((*S*)-1-(benzo[d]thiazol-2-yl)-3-(4-guanidinophenyl)-1-oxopropan-2-yl)amino)-1-oxo-3-cyclohexylpropan-2-yl)succinamide

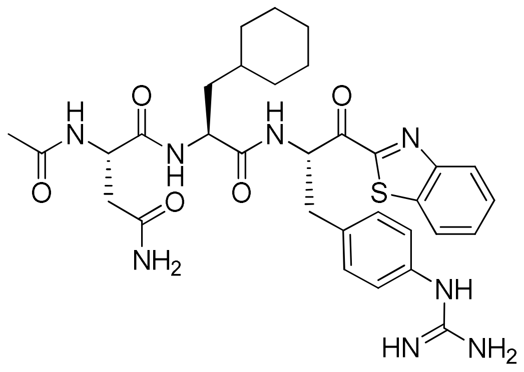

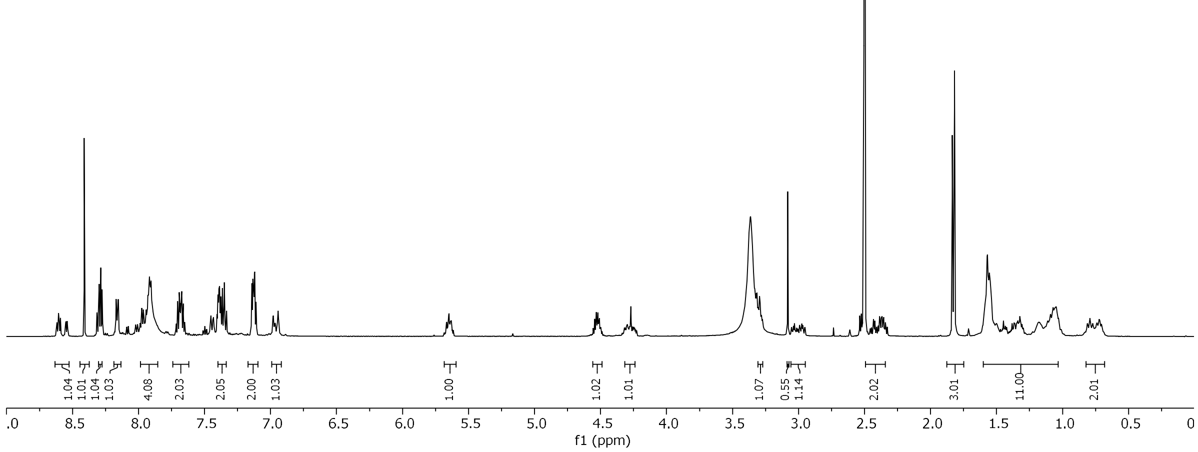

Figure S20a: ^{1}H NMR of **32**.

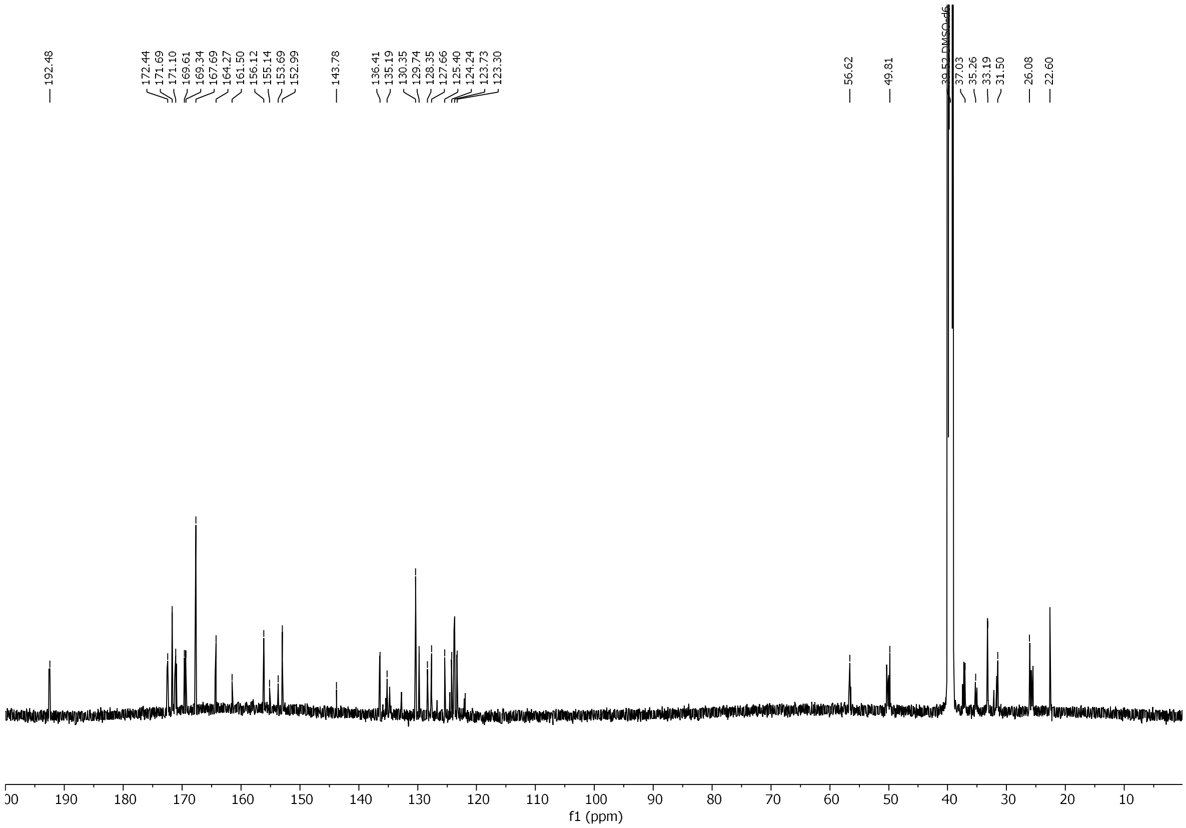

Figure S20b: ^{13}C NMR of **32**.

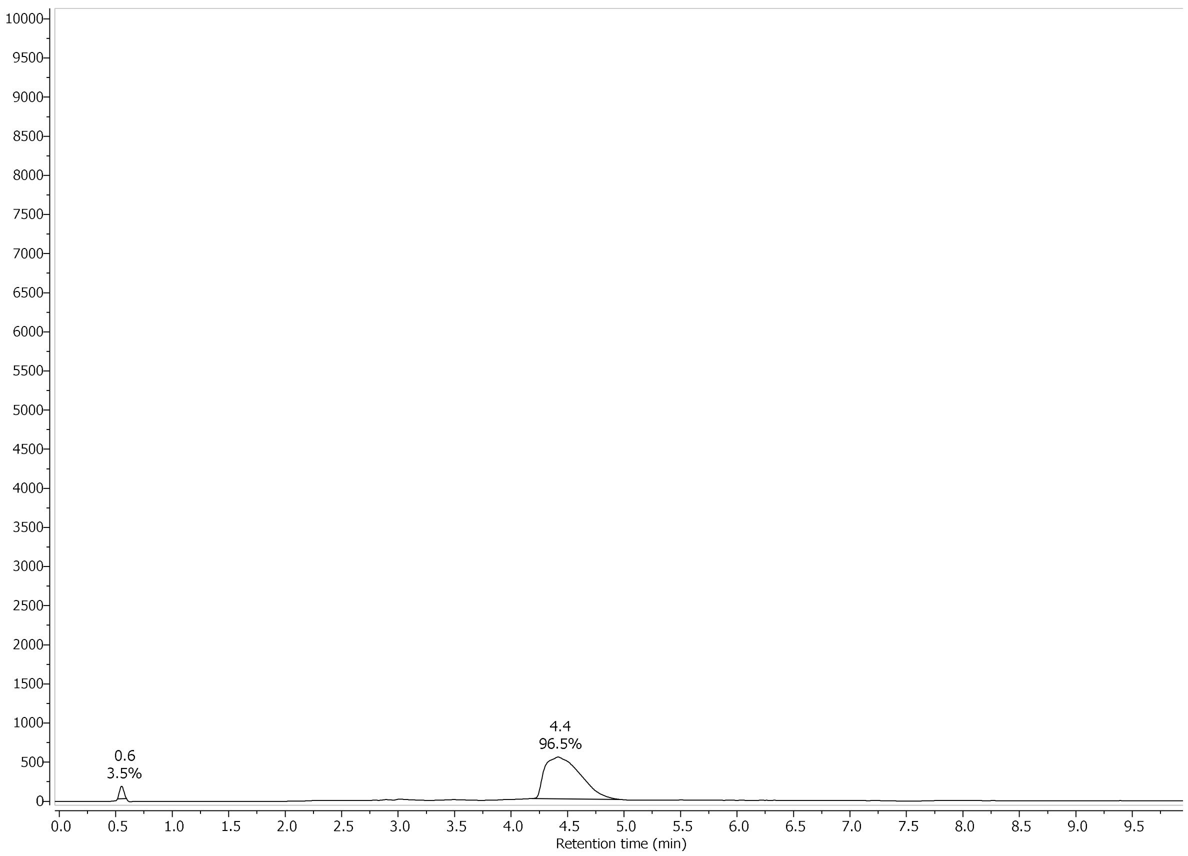

Figure S20c: HPLC Chromatogram of **32** at 210 nm.

33, (*S*)-2-Acetamido-*N*-((*S*)-1-(benzo[d]thiazol-2-yl)-3-(4-guanidinophenyl)-1-oxopropan-2-yl)-3-phenylpropanamide

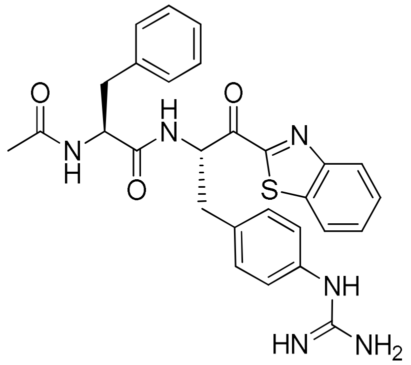

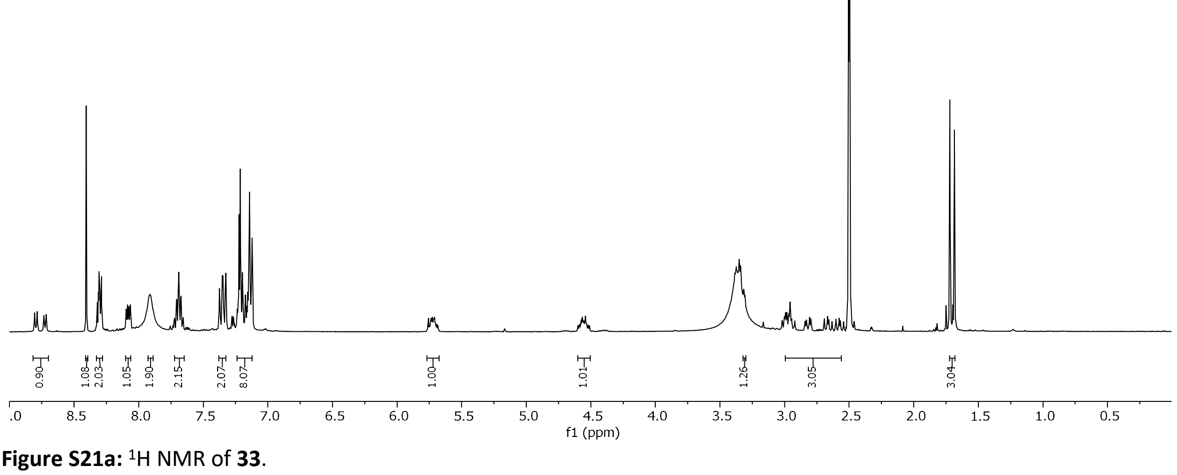

Figure S21a: ^{1}H NMR of **33**.

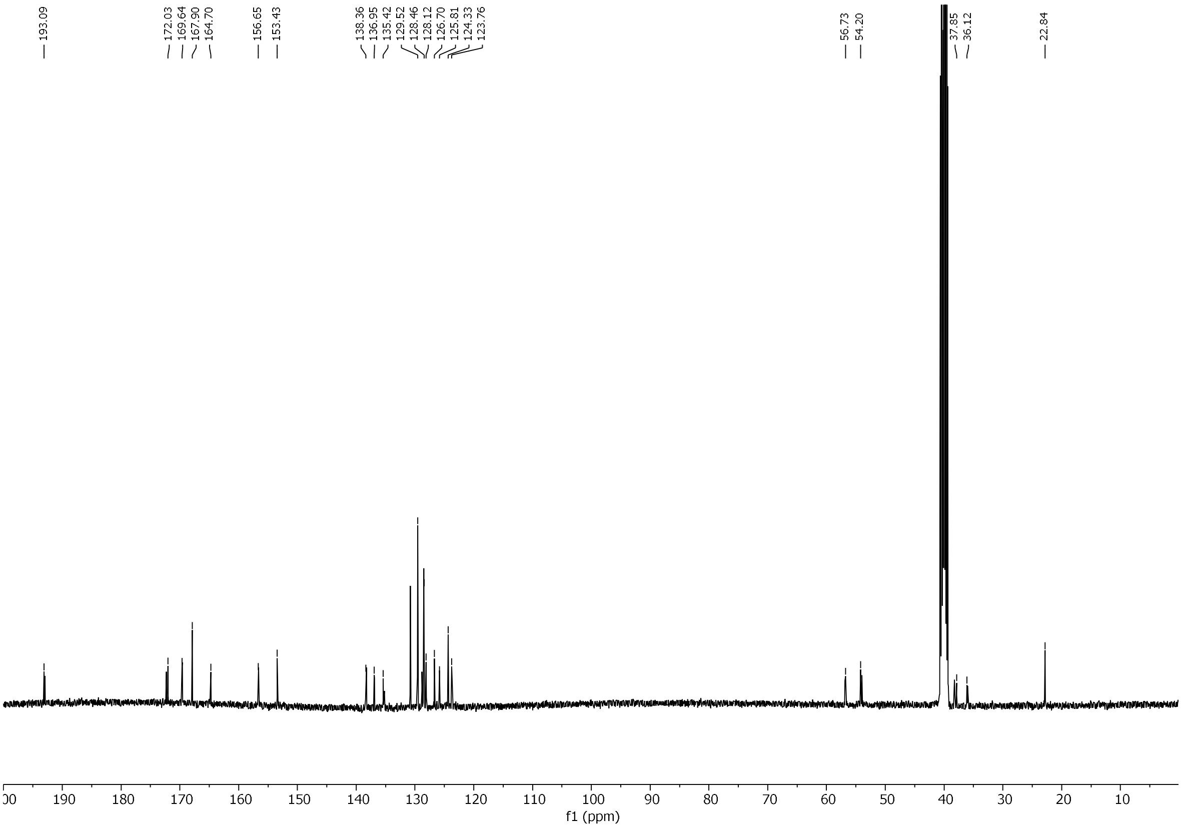

Figure S21b: ^{13}C NMR of **33**.

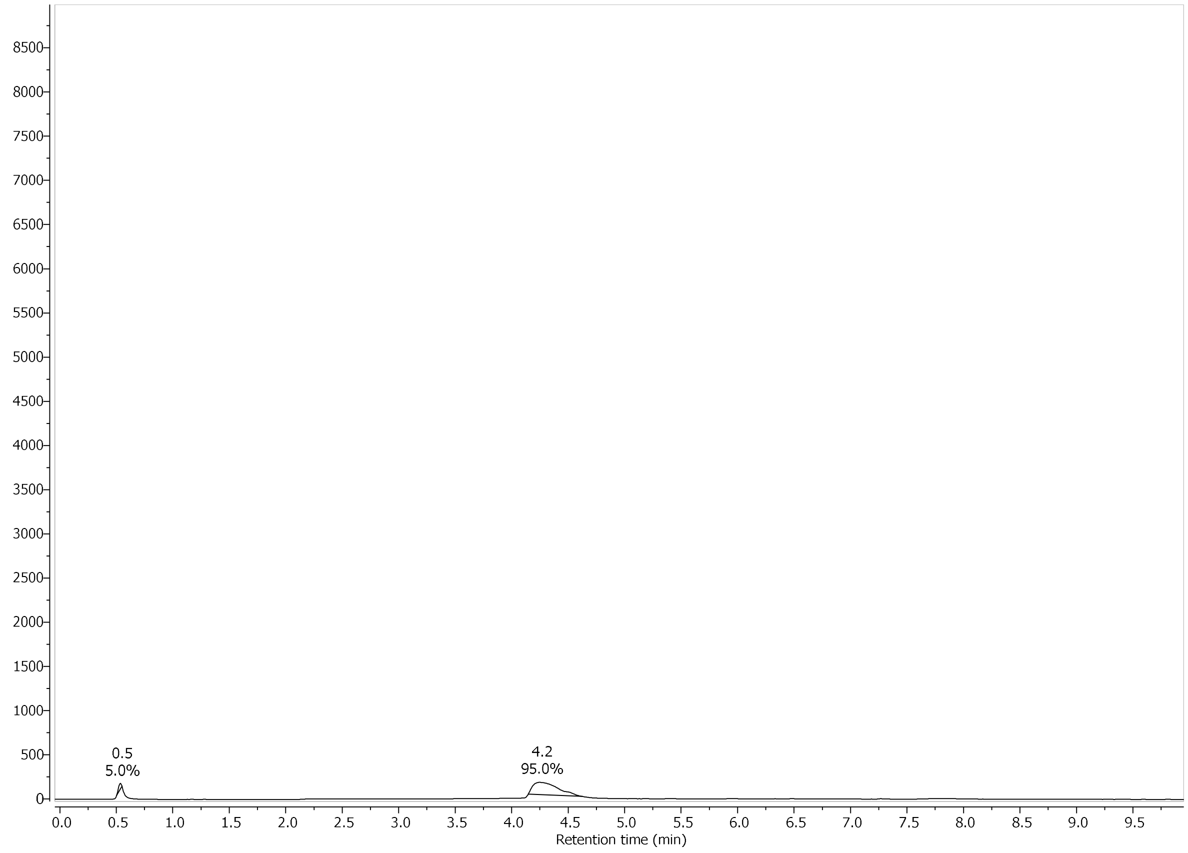

Figure S21c: HPLC Chromatogram of **33** at 210 nm.

References

1. Ludewig, S., Kossner, M., Schiller, M., Baumann, K. & Schirmeister, T. Enzyme Kinetics and Hit Validation in Fluorimetric Protease Assays. *Curr. Top. Med. Chem.* **2010** 10, 368–382.

2. Müller, P. *et al.* Investigation of the Compatibility between Warheads and Peptidomimetic Sequences of Protease Inhibitors—A Comprehensive Reactivity and Selectivity Study. *Int. J. Mol. Sci.* **2023** 24, 7226.

3. Wettstein, L. *et al.* Peptidomimetic inhibitors of TMPRSS2 block SARS-CoV-2 infection in cell culture. *Commun. Biol.* **2022** 5, 681.

4. Hammerschmidt, S. J., Maus, H., Weldert, A. C., Gütschow, M. & Kersten, C. Improving binding entropy by higher ligand symmetry? – A case study with human matriptase. *RSC Med. Chem.* **2023** 14, 969–982.

5. Ehnebom, J., Pusa, S., Björquist, P. & Deinum, J. Comparison of chromogenic substrates for tissue plasminogen activator and the effects on the stability of plasminogen activator inhibitor type-1. *Fibrinolysis and Proteolysis* **1997** 11, 287–293.

6. Tapp, H. J., Grundmann, C., Kusch, M. & Konig, H. Calibrating Thrombin Generation in Different Samples: Less Effort with a Less Efficient Substrate. *Open Atheroscler. Thromb. J.* **2009** 2, 6–11.

7. Edwards, S. T. *et al.* Differences between human and rabbit coagulation factor X—implications for in vivo models of thrombosis. *Thromb. Res.* **2002** 106, 71–79.

8. Wilkinson, D. J. *et al.* Matriptase Induction of Metalloproteinase-Dependent Aggrecanolysis In Vitro and In Vivo: Promotion of Osteoarthritic Cartilage Damage by Multiple Mechanisms. *Arthritis Rheumatol.* **2017** 69, 1601–1611.

9. Steinmetzer, T. *et al.* Secondary Amides of Sulfonylated 3-Amidinophenylalanine. New Potent and Selective Inhibitors of Matriptase. *J. Med. Chem.* **2006** 49, 4116–4126.

10. Kansy, M., Senner, F. & Gubernator, K. Physicochemical High Throughput Screening: Parallel Artificial Membrane Permeation Assay in the Description of Passive Absorption Processes. *J. Med. Chem.* **1998** 41, 1007–1010.

11. Sugano, K., Hamada, H., Machida, M. & Ushio, H. High Throughput Prediction of Oral Absorption: Improvement of the Composition of the Lipid Solution Used in Parallel Artificial Membrane Permeation Assay. *SLAS Discov.* **2001** 6, 189–196.

12. Avdeef, A. *Absorption and Drug Development*. (Wiley, 2003). doi:10.1002/047145026X.

13. Kerns, E. H. *et al.* Combined Application of Parallel Artificial Membrane Permeability Assay and Caco-2 Permeability Assays in Drug Discovery. *J. Pharm. Sci.* **2004** 93, 1440–1453.

14. St-Georges, C. *et al.* Modulating the selectivity of matriptase-2 inhibitors with unnatural amino acids. *Eur. J. Med. Chem.* **2017** 129, 110–123.

15. Costanzo, M. J. *et al.* Potent, Small-Molecule Inhibitors of Human Mast Cell Tryptase. Antiasthmatic Action of a Dipeptide-Based Transition-State Analogue Containing a Benzothiazole Ketone. *J. Med. Chem.* **2003** 46, 3865–3876.

16. Furlan, A. *et al.* Identification of new aminoacid amides containing the imidazo[2,1-b]benzothiazol-2-ylphenyl moiety as inhibitors of tumorigenesis by oncogenic Met signaling. *Eur. J. Med. Chem.* **2012** 47, 239–254.

17. Capaldo, L., Quadri, L. L., Merli, D. & Ravelli, D. Photoelectrochemical cross-dehydrogenative

coupling of benzothiazoles with strong aliphatic C–H bonds. *Chem. Commun.* **2021** 57, 4424–4427.

18. Kwon, H. *et al.* Structure-activity relationship studies of dipeptide-based hepsin inhibitors with Arg bioisosteres. *Bioorg. Chem.* **2021** 107, 104521.
